# Epithelial NF-κB shapes protective Treg cell responses to restrain intestinal inflammation

**DOI:** 10.1038/s41467-026-78796-z

**Published:** 2026-10-20

**Authors:** Luisa Barleben, Antony Julián Schneegans, Bora Uyar, Alix Bruneau, Giomar Vasileva, Franziska Flohr, Eleni Kabrani, Thomas Vasilakis, Linda Hammann, Pimrapat Gebert, Carola Dietrich, Bettina Bochow-Fitzner, Alexander Arnold, Somesh Sai, Florentine Gröne, Maike Sander, Thomas Conrad, Audrey Gérard, Ignazio Piseddu, David Anz, Christoph Jochum, Altuna Akalin, Linda Hammerich, Christoph S. N. Klose, Frank Tacke, Michela Di Virgilio, Marina Kolesnichenko

**Affiliations:** 1 Department of Hepatology and Gastroenterology, Campus Virchow-Klinikum and Campus Charité Mitte, Charité - Universitätsmedizin Berlin, Berlin, Germany; 2 Max Delbrück Center for Molecular Medicine in the Helmholtz Association, Berlin, Germany; 3 Bioinformatics and Omics Data Science Platform, Berlin Institute for Medical Systems Biology, Max Delbrück Center for Molecular Medicine, Berlin, Germany; 4 Laboratory of Genome Diversification and Integrity, Max Delbrück Center for Molecular Medicine in the Helmholtz Association, Berlin, Germany; 5 Institute of Clinical Pharmacology, Klinikum der Ludwig-Maximilians-Universität München, Munich, Germany; 6 Institute of Biometry and Clinical Epidemiology, Charité Universitätsmedizin Berlin, Berlin, Germany; 7 Genomics Technology Platform, Max Delbrück Center for Molecular Medicine in the Helmholtz Association, Berlin, Germany; 8 Core Facility Genomics, Berlin Institute of Health at Charité - Universitätsmedizin Berlin, Berlin, Germany; 9 Department of Pathology, Charité-Universitätsmedizin Berlin, Berlin, Germany; 10 Charité-Universitätsmedizin Berlin, Berlin, Germany; 11 The Kennedy Institute of Rheumatology, University of Oxford, Oxford, UK; 12 Department of Medicine II, Division of Gastroenterology and Hepatology, Klinikum der Ludwig-Maximilians-Universität München, Munich, Germany; 13 Charité - Universitätsmedizin Berlin, Corporate member of Freie Universität Berlin and Humboldt-Universität zu Berlin, Department of Microbiology, Infectious Diseases and Immunology, Hindenburgdamm 30, Berlin, Germany; 14 Cluster of Excellence ImmunoPreCept, Charité - Universitätsmedizin Berlin, Berlin, Germany

**Keywords:** Mucosal immunology, Stress signalling, NF-kappaB, Regulatory T cells, Chemokines

## Abstract

Inflammation resolution depends on coordinated communication between tissue compartments, yet how the intestinal epithelium shapes its immune microenvironment remains poorly understood. Here, using colonic biopsies from patients with ulcerative colitis (UC) and mouse models enabling tissue-wide visualization of NF-κB activity, we combine single-cell RNA sequencing and secretome analysis to define epithelial communication during colitis. We show that, paradoxically, the NF-κB-driven pro-inflammatory secretome of intestinal epithelial cells (IECs) is essential for recovery and prevents unrestrained inflammation. Mechanistically, epithelial NF-κB promotes CCL22-dependent regulatory T cell (Treg cell) recruitment and promotes a tolerogenic, pro-repair cell state, accompanied by induction of the lipid-metabolism-associated GDPD3 and CCR4. In UC, epithelial NF-κB is not uniformly activated but instead induces a selective pro-inflammatory transcriptional program while repressing targets regulating T cell recruitment. Thus, we identify dynamic epithelial–immune communication and demonstrate a protective IEC NF-κB–Treg cell axis important for resolution of intestinal inflammation.

## Abstract (web summary)

Inflammation and recovery from ulcerative colitis (UC) involves various immune cells and mediators. Here the authors examine the function of NFκB using patient samples and reporter mice to show that NFκB promotes T regulatory cell recruitment through CCL22 and a propensity towards repair.

## Introduction

Intestinal epithelium constitutes the primary barrier that segregates host tissue from the external environment. Defects in the epithelial barrier in inflammatory bowel diseases (IBD), both in ulcerative colitis (UC) and in Crohn’s disease (CD) precede clinical diagnosis by years^1,2^. Nevertheless, most studies have focused on deregulation of the immune response and communication from or between leukocytes in colitis^3^. Communication from intestinal epithelial cells (IEC) to their microenvironment and evolution of this “lexicon” as colitis progresses has been underexplored, even though epithelium is one of the largest organs in the body and can produce a diverse repertoire of cytokines, chemokines, and growth factors^4^. The majority of these secreted mediators are directly regulated by the transcription factor (TF) NF-κB^5^. NF-κB regulates the expression of over 400 genes, which control cellular processes ranging from proliferation to differentiation, apoptosis, and inflammatory response^5^. Yet which transcriptional programs NF-κB directs in IEC and which cell fate decisions it directs remains a hotly disputed topic. Older elegant studies, using knock-in and knockout mouse models of upstream kinases of NF-κB, demonstrated an anti-apoptotic role of the TF in IEC^6–8^. Specifically, knockouts of both the Ikappa B kinase gamma and beta (IKKγ and IKKβ) resulted in apoptosis in the IEC, a phenotype initially attributed solely to suppression of NF-κB^6,7^. However, more recent research points to pro-inflammatory and pro-apoptotic functions of NF-κB^9–11^. Significant obstacles to defining direct functions of NF-κB in IEC arise from the lack of unique and specific markers of NF-κB activity: there are no NF-κB-exclusive target genes that are not also regulated by other TFs. Post-translational modifications of NF-κB or nuclear localization of the subunits, for example, of the principal subunit RELA (p65), do not always equal transcriptional activity. Yet understanding how NF-κB directs epithelial communication in homeostasis and in colitis is of critical importance for the clinic: Genome-wide association studies and deep sequencing identified numerous members of the NF-κB signaling pathway, including its two inhibitors *NFKBIA* (IκBα) and *TNFAIP3* (A20), as risk loci for IBD^12,13^. Furthermore, the DNA-binding activity of NF-κB directly correlates with disease severity both in immune and epithelial cells^14^. Established IBD therapies, including corticosteroids and anti-TNF biologicals, directly inhibit NF-κB signaling^15,16^. Inhibition of immune NF-κB is associated with decreased proliferation of leukocytes and suppression of inflammation^5^ and is therefore likely a beneficial outcome. On the other hand, how suppression of NF-κB in epithelium disrupts communication from IEC to the microenvironment and affects mucosal healing in colitis remains unclear.

Here, we used UC patient samples and mouse models that allow us to visualize activated rather than merely present NF-κB at a single-cell resolution across all tissues. We thus characterize the transcriptomes and functions of IEC NF-κB in health, colitis, and recovery. Intestinal epithelium emerges as a potent immune organ that, through its changing NF-κB-driven transcriptomes and ensuing secretomes, shapes the mucosal immune compartment and promotes mucosal healing.

## Results

### Cell- and time-specific map of NF-κB in intestinal epithelium

**Fig. 1:**
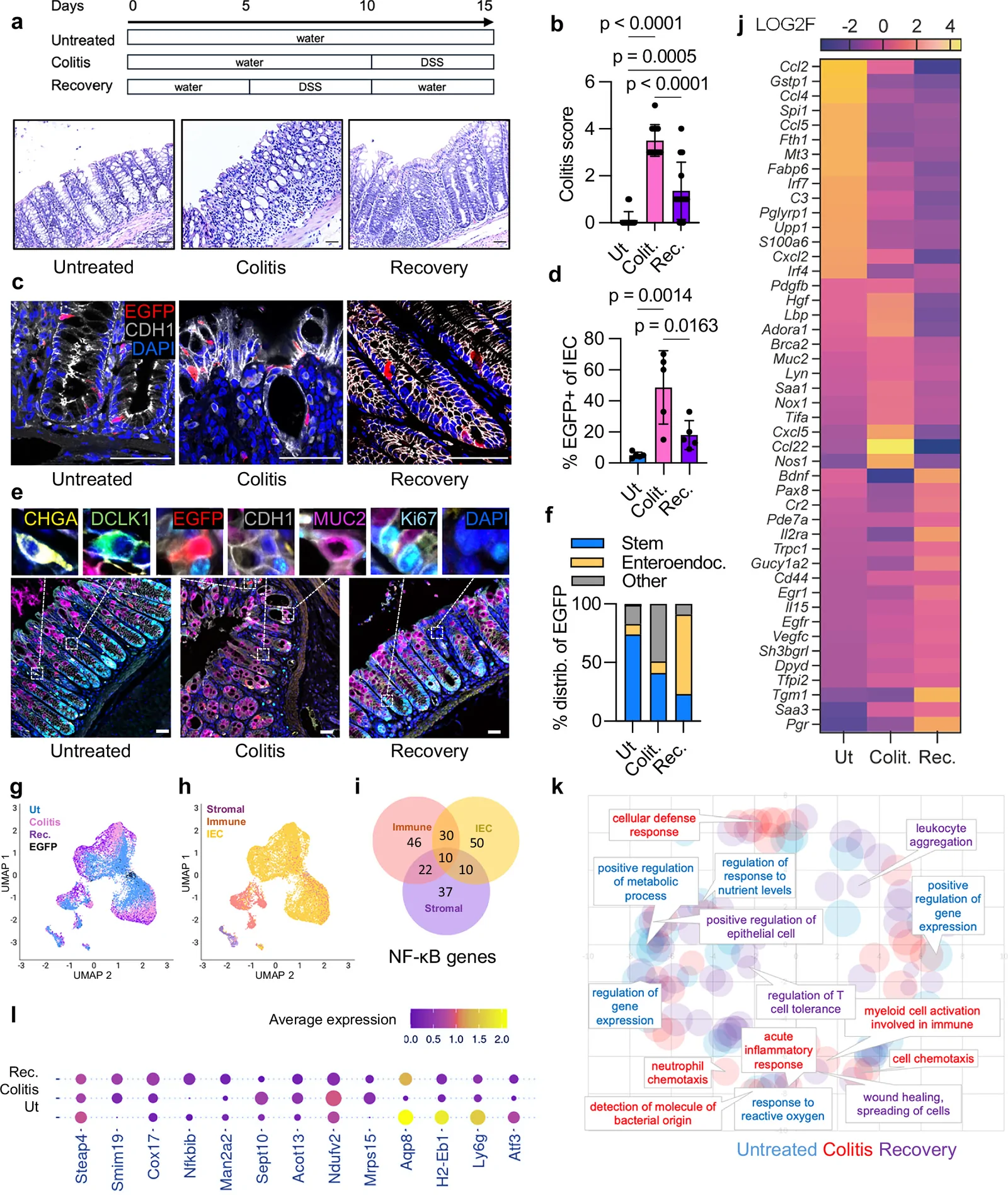
Cell- and time-specific map of NF-κB in intestinal epithelium. **a** Colon H&E from mice: Untreated - UT (water), Colitis (3% DSS for 5 days), or Recovery (3% DSS for 5 days, then water for 5 days). Representative sections shown from *n* = 10 mice. Scale: 50 µm. **b** Colitis score from (**a**). Analysis: one-way ANOVA with Tukey’s correction. n (mice) shown as individual points. Mean and SD shown. **c** Immunofluorescence from mice treated as in a. *n* = 4. Scale: 50 µm. **d** Quantitation of c. At least 100 IECs counted per mouse. n (mice) shown as individual points. Analysis: as in (**b**). Mean and SD shown. **e** Multiplex immunofluorescence (IF) staining on mice treated as in (**a**). Upper panels – zoom in on white squares below to visualize present IEC types. *n* = 3. Scale: 50 µm. **f** Quantitation of stem (Ki67+), enteroendocrine (CHGA + ), or other cell types as % of total gut epithelial (CDH1+) cells. *P* < 0.0001. One-way ANOVA with Tukey’s correction, *n* = 3, with at least 100 CDH1+ cells per mouse. **g** scRNAseq performed on nuclei from the whole gut of mice treated as in a. with *n* = 3 (Recovery), *n* = 4 (Ut), and *n* = 4 (Colitis), individual mice pooled per group. UMAP representation of treatment groups. EGFP+ cells shown in black. **h** UMAP as in (**g**). Stromal (all), Immune (all), and IEC (all) **i** Differential expression analysis for scRNA from h. of NF-κB target genes showing a significant increase in expression in IEC vs stromal vs immune cells across all three conditions. Adj. *P* value cut-off for genes: 0.05. **j** scRNA analysis of validated NF-κB target genes expressed in CDH1+ cells across 3 conditions. Only top hits shown. **k** Semantic space (x and y axis) representation of REVIGO analysis of GO terms of NF-κB target genes from (**i**). Top GO terms shown. *P* < 0.05 with Bonferroni correction. **l** Gene markers of activated NF-κB from scRNA: Dot plot of top differentially regulated genes in EGFP + IEC across the 3 conditions. Average expression shown. % expression shown across all EGFP+ cells. Source data are provided as a Source Data file.

To allow cell-specific identification of cells with activated rather than merely present NF-κB across different tissues, we took advantage of our reporter mouse model, where NF-κB binding sites are placed upstream of *EGFP*^10,17^ (Supplementary Fig. 1a–c). This strategy allowed us to track in space and time which cells exhibited activated NF-κB across different tissues in colitis (Supplementary Fig. 1a–c). To induce colitis, we treated one group of mice with 3% Dextran Sulfate Sodium (DSS) for five days (Fig. 1a, b), which mimics many key aspects of UC^18^. Another group of mice was allowed to recover for five days after DSS treatment to observe events in early recovery. The control group, from here on referred to as “untreated”, was used to monitor NF-κB under homeostasis. As expected, acute treatment with DSS led to colitis, evidenced by inflammatory cell infiltration in the mucosa and submucosa, and crypt loss (Fig. 1a, b). Mice experienced delayed weight loss from day 5, indicative of colitis, and shortening of the colon (Supplementary Fig. 1d, e). After 5 days of recovery, crypts mostly regenerated; however, immune infiltrates remained elevated (Fig. 1a, b). To characterize IEC with activated NF-κB, colon tissues from mice treated as above were stained against EGFP and a marker of IEC and adherens junctions, E-Cadherin, CDH1 (Fig. 1c). Whereas under homeostasis less than 5% of IEC were EGFP+, during acute colitis, nearly 50% of the remaining IEC displayed activated NF-κB (Fig. 1d). To further characterize in which cells NF-κB was activated in homeostasis, in colitis, and in recovery, we performed multiplex staining with intestinal cell-type specific markers (Fig. 1e). In striking contrast to the untreated mice, where activated NF-κB localized to Ki67+ stem cells, in recovery activated NF-κB was predominantly detected in CHGA+ enteroendocrine cells (Fig. 1e, f).

To confirm these findings, we isolated EGFP+ (activated NF-κB) IEC by fluorescence-activated cell sorting (FACS) (Supplementary Fig. 1f). We analysed the expression of stem (*Lgr5*) and enteroendocrine cell (*Chga*) markers, as well as bona-fide NF-κB target genes, including pro-inflammatory/ pro-apoptotic *Tnf, Bbc3, Tnfaip3*, and anti-apoptotic *Bcl2* and *Bcl2l1* (Supplementary Fig. 1g–j). Isolated EGFP+ but not bulk IEC were enriched for *EGFP* as expected (Supplementary Fig. 1g), as well as for markers for stem- and enteroendocrine cells, but not for other IEC cell types (Supplementary Fig. 1h). Interestingly, EGFP+ cells were enriched for pro-inflammatory/ pro-apoptotic (Supplementary Fig. 1i), but not anti-apoptotic NF-κB target genes (Supplementary Fig. 1j). These data indicate that activation of NF-κB depends both on cell type and on stage of colitis.

To further characterize the transcriptomes driven by NF-κB across different cell types in the intestinal mucosa, we performed single-cell sequencing (scRNA) on colonic tissues of *κEGFP* mice using the same conditions as above (Fig. 1g–k, Supplementary Fig. 1k and Supplementary Data 1). *EGFP* expressing cells were detected across all three conditions and in epithelial, immune and stromal cells (Fig. 1g, h). We first determined which of the already validated NF-κB target genes^19^ were expressed in a compartment-specific manner (Fig. 1i). Strikingly, differential expression analysis of scRNA revealed that the same stimulus (e.g., DSS) triggers expression of unique and divergent sets of NF-κB genes in the immune, stromal, and IEC compartments. Indeed, only ten genes were shared among the three compartments (Fig. 1i and Supplementary Data 2). In line with thorough prior studies of immune cells in murine colitis and in IBD^14,20^, our findings confirm the clear pro-inflammatory role of NF-κB in leukocytes (Supplementary Data 2). The role of epithelial NF-κB, however, remained controversial, and therefore here we aimed to decipher how IECs communicate with their environment. We focused on two aspects of the epithelial response: genes affecting IEC in a cell-intrinsic manner and genes coding for the epithelial secretome (extrinsic output), which affect other tissues (Fig. 1j, k). We detected a clear demarcation of genes that were selectively regulated under homeostasis only or only in colitis or in recovery by NF-κB. Among all genes analysed, *Ccl22* exhibited the strongest induction in the acute colitis setting (Fig. 1j). Importantly, we observed a marked shift from homeostatic processes, including “regulation of gene expression” and “regulation of metabolic processes”, toward clearly extrinsic programs in colitis, such as “acute inflammatory response” and “myeloid cell activation” (Fig. 1k). These data indicate that NF-κB prioritizes distinct transcriptional programs in homeostasis versus colitis, redirecting epithelial gene expression from intrinsic maintenance pathways toward cytokine- and chemokine-encoding genes that mediate communication with the surrounding microenvironment.

To identify NF-κB targets and characterize gene sets in cells with ongoing high NF-κB activity, we analysed gene expression in IEC with high versus low *EGFP*. We detected several genes that were enriched only in cells with high *EGFP*. Intriguingly, many of these were enriched only at a particular time point in colitis. For example, Aquaporin 8 (*Aqp8*), whose suppression was detected in epithelium of IBD patients^21,22^, was expressed under homeostasis and suppressed in colitis (Fig. 1l, Supplementary Fig. 1k and Supplementary Data 1). In IEC of DSS-treated mice, genes regulating mitochondrial dynamics, including *Ndufv2, Mrps15*, and *Lsm6,* were strongly upregulated. In addition, *H2-Eb1* (murine major histocompatibility complex II, MHC II) was significantly enriched in EGFP*+* cells. We did not detect significant expression of intrinsic anti-apoptotic mediators in any of the three conditions. Instead, NF-κB regulated expression of distinct and condition-dependent subsets of pro-inflammatory genes associated with chemotaxis of different types of leukocytes (Fig. 1j, k). In summary, we defined specific transcriptomes of NF-κB in IEC, in immune, and in stromal cells. Furthermore, we demonstrated that distinct NF-κB gene sets in IEC are activated during initiation, progression, and recovery of colitis.

### Activation of epithelial NF-κB is necessary for regeneration

**Fig. 2:**
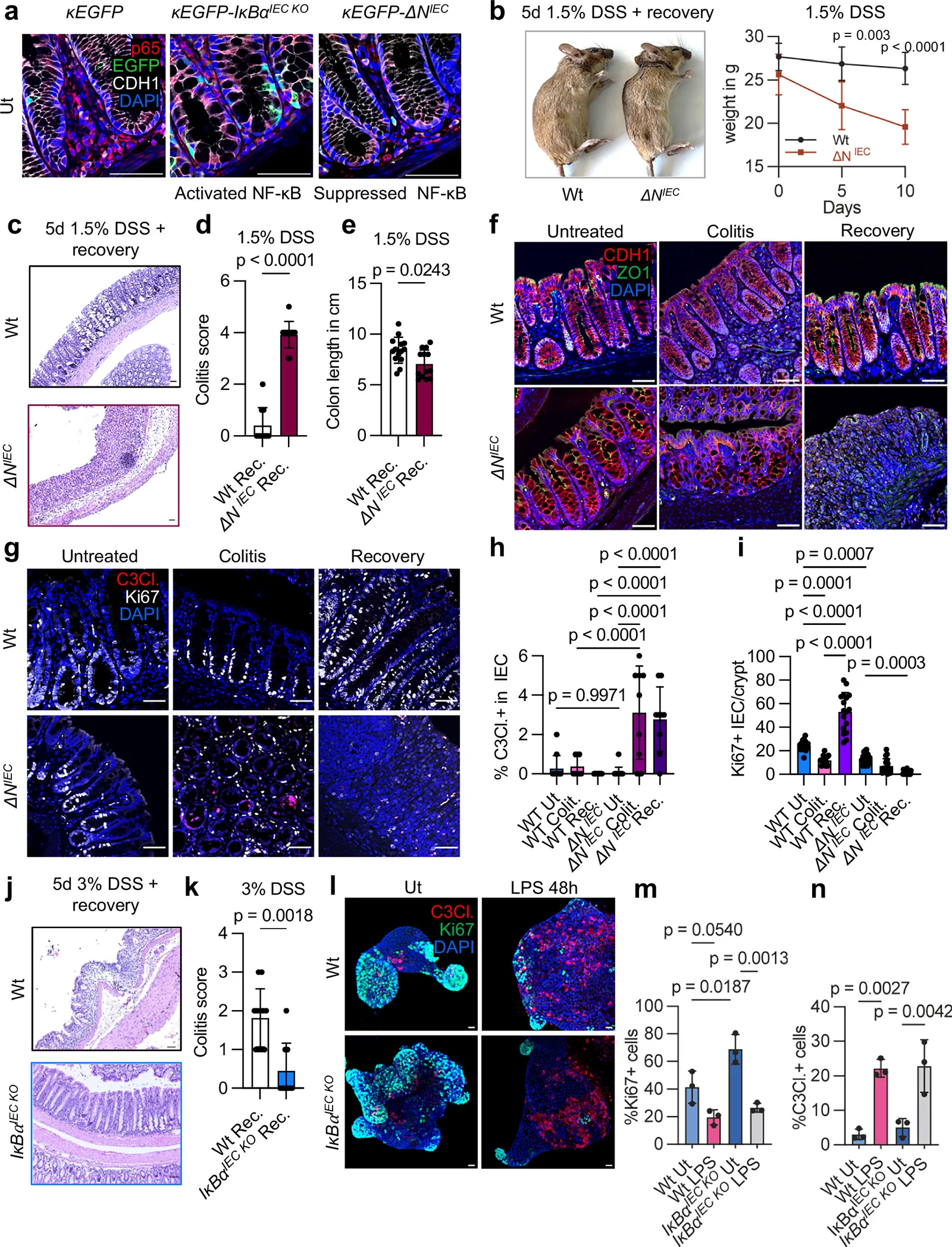
Pro-inflammatory epithelial NF-κB is necessary for recovery. **a** Immunofluorescence (IF) against p65 (NF-κB subunit) – EGFP (activated NF-κB), and CDH1 (epithelium) in colons of untreated mice, Wt with *κEGFP* and mice with either activated or suppressed NF-κB. Scale: 50 μm. **b** Mice with suppressed NF-κB (right) and Wt (LoxP-) littermates treated with 1.5% DSS over 5 d, followed by 5 d water. Representative image shown from *n* = 10 animals. Right graph: weight of mice during the duration of the experiment. Mean and SD shown. *n* = 10 for all groups apart from Wt Ut (where *n* = 11). Analysis: Welch's *t-*test, two-sided. **c** H&E from mice in (**b**). Scale: 50 μm. **d** Colitis score from mice treated as in (**b**). mouse number (n) shown in the graph. Analysis: Mann–Whitney U test (2-tailed). Mean and SD shown. **e** Colon length from mice treated as in b-c. Analysis as above. Mean and SD shown. **f** IF staining of adherence and tight junctions (CDH1 and ZO1) in mice treated as in (**b**–**e**). Scale: 50 μm. *n* = 3. **g** IF against cleaved caspase 3 (apoptosis) and Ki67 (proliferation). Mice as in b-e. Scale: 50 μm. *n* = 3. **h**,** i** Quantitation of cleaved caspase 3+ cells as % of IEC and % Ki67+ cells per crypt. Statistical analysis: one-way ANOVA with Tukey’s correction. n (representing mice = biological replicates) is shown as individual points. Mean and SD shown. **j** H&E of mice with activated NF-κB (*IκBα* ^*IEC KO*^) and Wt littermates treated with 3% DSS over 5 d and followed by 5 d water. Scale: 50 μm. **k** Colitis score from (**j**). Analysis: as in (**d**). *n* = 5. Mean and SD shown. **l** IF of organoids derived from Wt animals or mice with activated IEC NF-κB stained against cleaved caspase 3 and Ki67 treated with 50 μg/ml LPS over 48 h. Representative images. Colon organoids derived from *n* = 3 mice per condition. Scale: 50 μm. **m**,** n**. Quantitation of Ki67+ and cleaved caspase 3+ cells as % of total cells from l. Analysis as in d. *n* = 3. Mean and SD shown. Source data are provided as a Source Data file.

Our findings suggest that the specific suppression of epithelial NF-κB would ameliorate colitis by restraining leukocyte recruitment. To test this hypothesis, we employed our established mouse model, *ΔN*^*IEC*^, where the truncated gene of the direct inhibitor of NF-κB, *Nfkbia*, is knocked in and expressed only in the intestinal epithelium^17^. This allowed us to directly suppress NF-κB rather than its kinases, which we recently demonstrated to have also NF-κB-independent roles in the regulation of inflammatory cytokines^23^. In parallel, we used mice with knockout of the direct inhibitor *Nfkbia*, *IκBα* ^*IEC KO*^, which exhibit constitutive activation of NF-κB specifically in IEC without affecting the function of the upstream kinases^10,24^. We crossed *ΔN*^*IEC*^ and *IκBα* ^*IEC KO*^ mice to *κEGFP* animals to directly visualize suppression and activation of the IEC NF-κB (Fig. 2a). Importantly, as mentioned above, nuclear localization or post-translational modifications of the principal subunit, p65 (RelA), were not indicators of ongoing NF-κB *activity*, as evidenced here by unaffected amount, and/or phosphorylation or acetylation status of p65 (Fig. 2a and Supplementary Fig. 2a). Indeed, both acetylated and phosphorylated p65 were abundant in crypts, yet only a fraction of those cells was EGFP+ and therefore contained active NF-κB driving transcription.

*κEGFP IκBα* ^*IEC KO*^ and *κEGFP ΔN*^*IEC*^ mice were born according to the expected Mendelian ratios. Their colons appeared phenotypically inconspicuous on visual inspection with an increased number of isolated lymphoid follicles in mice with activated epithelial NF-κB (Supplementary Fig. 2b, c). Under homeostatic conditions, approximately 15% of IEC were EGFP⁺ in *IκBα* ^*IEC KO*^ mice, representing a threefold increase compared with wild-type controls (Supplementary Fig. 2d). To induce colitis, we initially treated *IκBα* ^*IEC KO*^ and *ΔN*^*IEC*^ mice and their respective wild-type (Wt) co-housed littermates with low-dose DSS (1.5%), using the same time points as above. Whereas Wt littermates were mostly unaffected, strikingly, mice with suppressed NF-κB in the epithelium continued losing weight and did not recover, even when the DSS treatment was ended (Fig. 2b–e). Severe colitis was evidenced by colon shortening and mucosal and submucosal inflammation 5 days after low-dose DSS treatment (Fig. 2c–e). In mice with suppressed epithelial NF-κB, over 50% of colonic epithelium was destroyed in acute colitis (Supplementary Fig. 2e), and loss progressed even after DSS removal (Fig. 2c–e and Supplementary Fig. 2e), indicative of hyperinflammation.

We hypothesized that several factors could account for the hypersensitivity of *ΔN*^*IEC*^ mice to colitis: compromised epithelial barrier integrity, increased susceptibility to apoptosis, or stunted proliferative capacity (intrinsically mediated by IEC or extrinsically mediated by other tissues). We firstly asked if these processes were already compromised even before the DSS treatment: we did not detect significant changes in the epithelial barrier under homeostasis in the *ΔN*^*IEC*^ mice (Fig. 2f). After DSS treatment, collapse of the epithelial barrier was reflected by a reduction in markers of both tight junctions and adherens junctions, namely zonula occludens-1 (ZO-1) and E-cadherin (CDH1). We also did not detect an increased rate of apoptosis due to suppression of NF-κB in IEC under homeostasis (Fig. 2g, h). Therefore, rather than representing the primary cause for the lack of recovery in mice with suppressed epithelial NF-κB, apoptosis and epithelial barrier collapse appeared to be a consequence of other IEC defects. In contrast, a significant decrease in Ki67+ stem cells in the mice (Fig. 2i, g) pointed to a stunted proliferative capacity that could compromise barrier regeneration and recovery.

To resolve whether constitutive epithelial NF-κB activation protects against DSS-induced colitis, we treated *IκBα* ^*IEC KO*^ mice and their co-housed Wt littermates with full-dose DSS (Fig. 2j, k and Supplementary Fig. 2f–h). Wt mice experienced moderate colitis, and upon recovery, their colons contained numerous hyperplastic crypts, as expected. Intriguingly, mice with activated epithelial NF-κB contained hyperplastic crypts already under homeostasis and were mostly resistant to colitis (Supplementary Fig. 2g, h). To determine whether epithelial NF-κB regulates epithelial regeneration after stress in an IEC-autonomous or cell-extrinsic manner (e.g., factors from stromal or immune cells contribute to the observed phenotype), we established organoids from crypts of *IκBα* ^*IEC KO*^ and *ΔN*^*IEC*^ mice. After passaging, organoids contained IEC exclusively. *IκBα* ^*IEC KO*^ organoids grew and differentiated at an increased rate (Supplementary Fig. 2i, j). Crypts from mice with suppressed NF-κB initially appeared normal but failed to form organoids (Supplementary Fig. 2k). To determine if resistance to barrier damage is intrinsic to IEC with activated NF-κB, we then treated organoids from *IκBα* ^*IEC KO*^ mice with bacterial endotoxin Lipopolysaccharide (LPS) (Fig. 2l–n). We detected a significant decrease in proliferating Ki67+ cells in both groups, but did not observe an increased resistance to LPS (Fig. 2l–n). This observation suggests that extrinsic factors are required to mediate protective functions associated with activated epithelial NF-κB. Stromal cells provide critical factors for stem cell renewal, including WNT ligands^25^. Supplementation of *ΔN*^*IEC*^ organoids with extra WNT or addition of medium derived from primary fibroblasts of Wt sibling mice did not restore proliferative capacity (Supplementary Fig. 2k), suggesting that the required extrinsic factors are not likely derived from stromal cells. Taken together, these data indicate that epithelial NF-κB and its pro-inflammatory transcriptomes protect against unrestrained inflammation and that downstream, extrinsic factors from other tissues are required for these protective functions.

### Epithelial NF-κB shapes the mucosal immune cell compartment

**Fig. 3:**
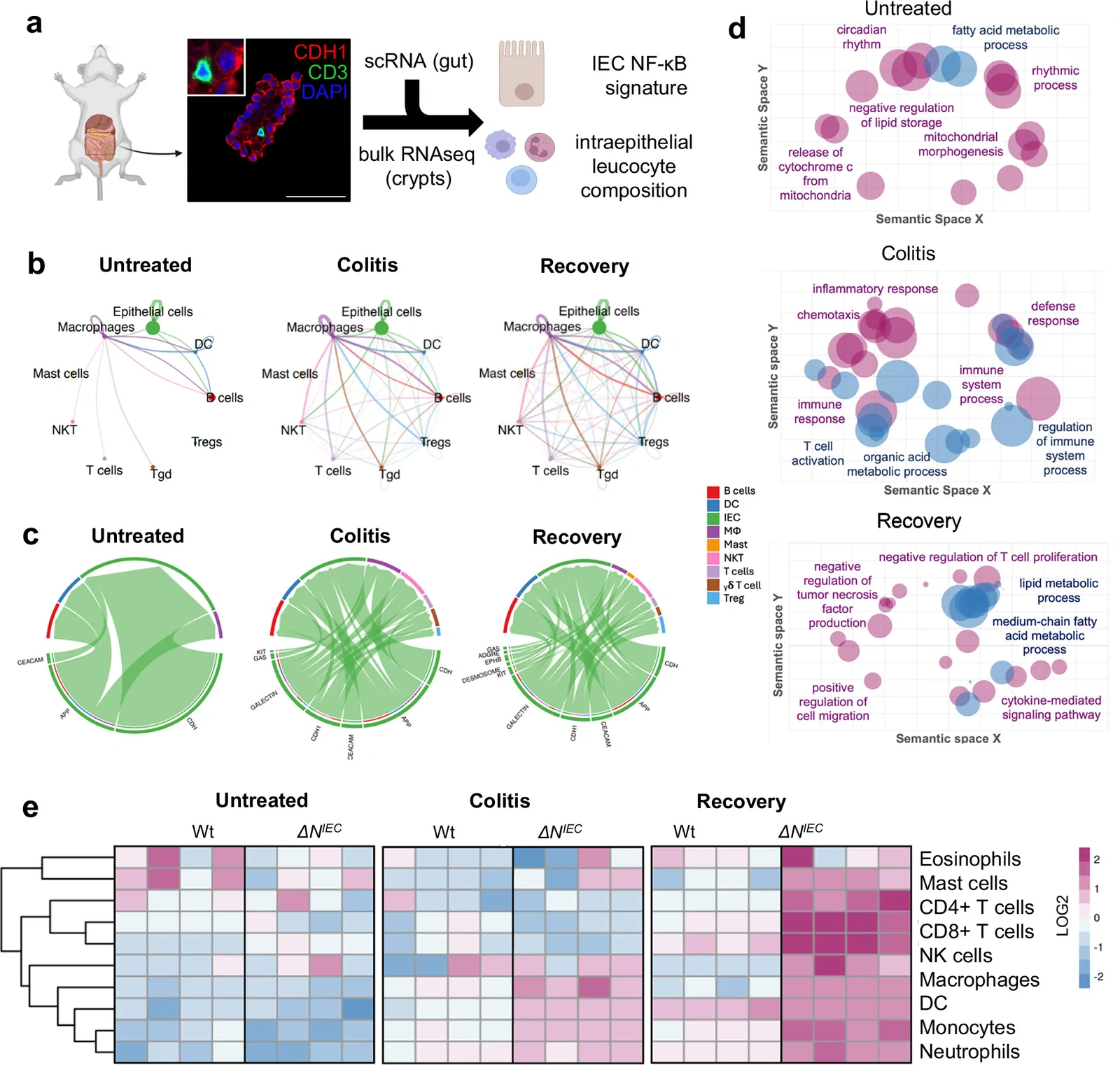
NF-κB balances expression of genes mediating innate vs adaptive immune cell recruitment. **a** Crypts were isolated from mice treated as before (untreated, DSS, or DSS and recovery). The immunofluorescence image shows the presence of intraepithelial CD3+ cells in CDH1+ crypts. RNAseq was performed on crypts of *ΔN*^*IEC*^ mice and Wt littermates. Deconvolution was performed using scRNA signatures (Fig. 1) of epithelial versus immune cells. Parts of the image were created in BioRender. Kolesnichenko, M. (2026) https://BioRender.com/7o60ccc. **b** CellChat analysis of epithelial-immune interactions. Cell-cell interaction strength is represented by arrows. **c** Chord diagram showing CellChat receptor-cell type and target interactions. **d** RNAseq GO terms reduced by REVIGO and visualized on semantic space. Magenta bubbles represent GO terms enriched in crypts of *ΔN*^*IEC*^ mice vs Wt littermates, blue bubbles represent GO terms depleted in crypts of *ΔN*^*IEC*^ mice. **e** Immune cell type signature scores in bulk RNA-seq data. Cell type signatures were obtained from xCell and mouse genes. The single-sample gene-set scoring was done using the singscore R package. Source data are provided as a Source Data file.

We next sought to dissect the NF-κB-driven transcriptional programs in IEC that enable recovery from colitis. We identified partners in the IEC NF-κB crosstalk using a combination of scRNA from the whole gut, combined with bulk RNAseq from crypts to differentiate between IEC versus intraepithelial leukocyte contribution (Fig. 3a). We firstly analysed IEC crosstalk to predict IEC-immune interaction in an unbiased manner. CellChat analysis^26^ of our scRNA data revealed few interaction partners during homeostasis and a dramatic increase in partners in colitis and recovery (Fig. 3b). Importantly, recipients of IEC communication extended to lymphocytes, including regulatory T (Treg) cells and γδ T cells. Predicted interaction with these was mediated via Galectin and Amyloid Precursor protein (APP) receptor-ligand interactions (Fig. 3c). Thus, the dominant IEC-IEC interactions in homeostasis were exchanged for the IEC-immune interactions in colitis and in recovery.

We next analysed the NF-κB transcriptomes directing these interactions in IEC. We isolated colonic crypts from *ΔN*^*IEC*^ mice and their Wt littermates treated with low-dose DSS and performed bulk RNAseq (Supplementary Fig. 3a, b). Isolation of crypts instead of epithelium allows for more rapid processing and therefore reduces stress associated with extraction. Importantly, it also allows for retention of intraepithelial infiltrating leukocytes (Fig. 3a), whose presence can be detected by deconvolution of bulk RNA data. Under homeostatic conditions, we observed repression of NF-κB and TNF signaling pathways, as expected, and of TGFβ, known to regulate immune cells (Supplementary Fig. 3c). Interestingly, in colitis, we detected aberrant NF-κB activity rather than uniform suppression in crypts of *ΔN*^*IEC*^ mice (Supplementary Fig. 3c), with downregulation of proliferative *Fos* and *Jun*, immunomodulatory *Il18* and *H2-Eb1* and of concomitant activation of NF-κB targets regulating inflammatory response, including of *Il1a*, *Il6*, and *Cxcl2* (Supplementary Fig. 3d and Supplementary Data 3). GO analysis revealed that under all three conditions (homeostasis, colitis, and early recovery) genes positively regulating metabolism constituting processes including “lipid metabolism”, “sterol biosynthesis” and “cholesterol biosynthesis” were suppressed in *ΔN*^*IEC*^ mice. Conversely, genes regulating immune processes and adaptive versus innate immune cell recruitment were positively regulated (Fig. 3d). Remarkably, “chemotaxis” genes were enriched in *ΔN*^*IEC*^ mice, whereas genes regulating T cell functions were suppressed (Fig. 3d middle and lower panels). These findings indicate that while genetic suppression of NF-κB effectively limits pro-inflammatory gene expression under homeostatic conditions, during colitis, additional pathways operate in parallel, driving sustained and unrestrained induction of inflammatory mediators.

**Fig. 4:**
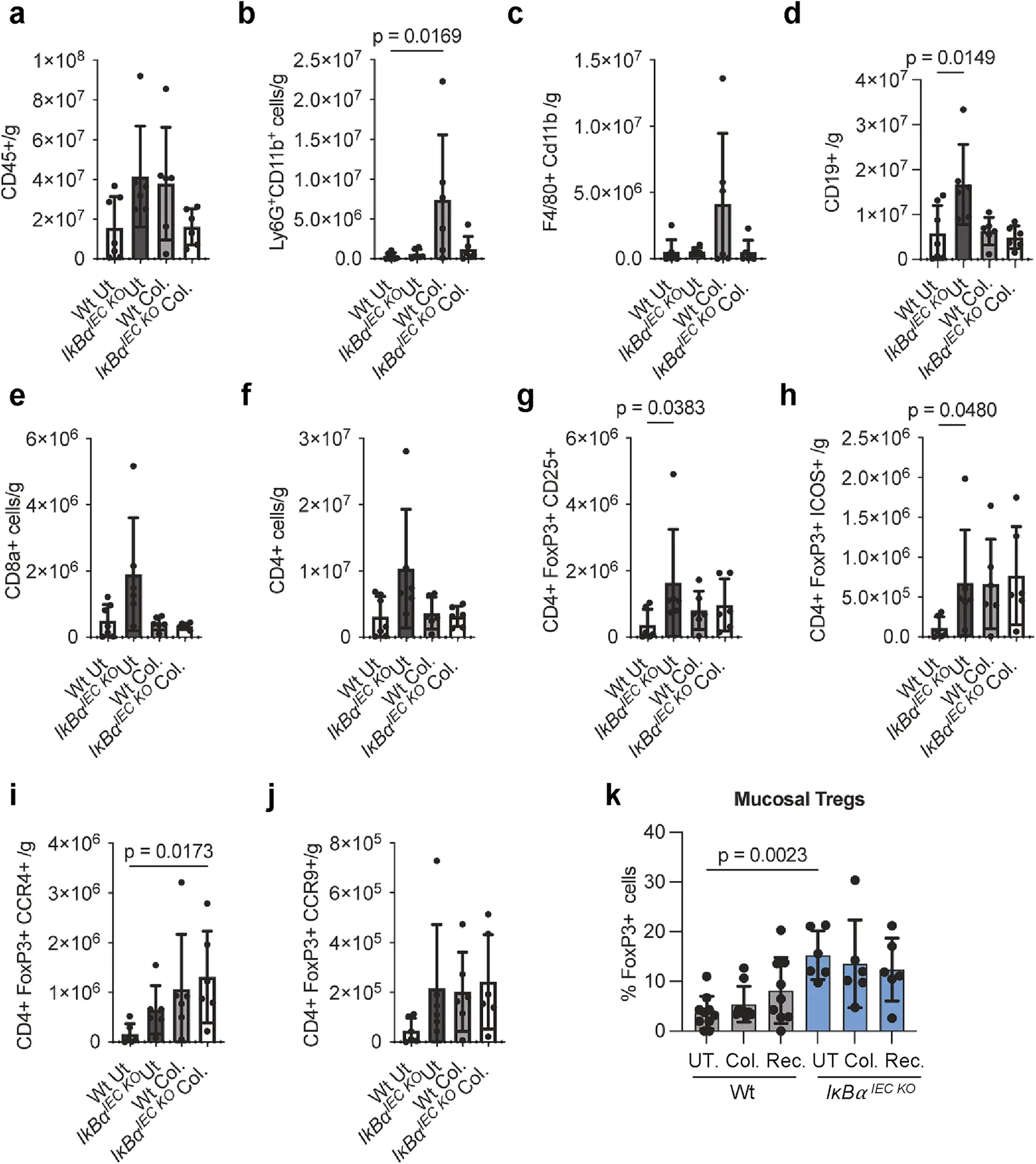
Epithelial secretome shapes the mucosal immune compartment. **a–j** Quantification of immune phenotyping by flow cytometry on leukocytes from isolated lamina propria immune cells from the distal colons from *IκBα* ^*IEC KO*^ and Wt mice (*n* = 6 per group). Analysis: Kruskal-Wallis ANOVA with Dunn’s multiple testing correction. Mean and SD shown. *n* = 6 mice. **k** Quantitation of FoxP3+ CD4+ as % of total mucosal leukocytes. Analysis: One-way ANOVA with Tukey’s correction. Mean and SD shown (*n* = 6). Source data are provided as a Source Data file.

To directly analyse leukocytes recruited to crypts in vivo, we took advantage of an isolation technique that allows for retention of selectively intraepithelial immune cells (Fig. 3a). To determine how epithelial NF-κB affects composition of infiltrating leukocytes across different conditions, we performed deconvolution of sequencing data, revealing that in colitis neutrophils, monocytes, and macrophages were enriched in crypts of mice with suppressed NF-κB, whereas CD4 + T cells were depleted (Fig. 3e and Supplementary Data 3). In recovery, expression of macrophage markers returned to the level observed in the crypts of untreated mice. However, this was not the case in crypts of the *ΔN*^*IEC*^ mice, where markers of all immune cell types analysed increased even further (Fig. 3e). To validate our findings, we performed immune phenotyping by flow cytometry on leukocytes from the spleen and distal colons from *ΔN*^*IEC*^, *IκBα* ^*IEC KO*^, and Wt mice under homeostasis and colitis (Fig. 4a–j, Supplementary Fig. 4a–j and Supplementary Fig. 5). We detected a significant increase in Ly6G + CD11b+ neutrophils in Wt mice in colitis as expected (Fig. 4b and Supplementary Fig. 2a). No equivalent increase was observed in Ly6G+ CD11b+ cells in *IκBα* ^*IEC KO*^ mice in colitis. Strikingly, CD4+ FoxP3+ CD25+ and CD4+ FoxP3+ ICOS+ Treg cells in the distal colon of the *IκBα* ^*IEC KO*^ mice showed a significant increase relative to Wt mice already under homeostasis (Fig. 4g, h, Supplementary Fig. 1 and 2f, g). Analysis of Treg cell distribution in the distal colon by immunofluorescence showed more than a sixfold increase in mucosal FoxP3⁺ CD4⁺ Treg cells under homeostasis in distal colons of *IκBα* ^*IEC KO*^ animals relative to that of Wt, but no further induction during colitis (Fig. 4k and Supplementary Fig. 4k), supporting our observations from immunophenotyping.

In summary, using flow cytometry, CellChat from sequencing data, and immunofluorescence, we demonstrated that epithelial NF-κB shapes the composition of intestinal mucosal leukocytes. Activated epithelial NF-κB promotes accumulation of Treg cells in the distal colon. We therefore hypothesized that the inability to induce IEC NF-κB prevents the accumulation of Treg cells and promotes unrestrained colitis.

### Epithelial NF-κB shapes a protective Treg cell phenotype that limits inflammation

It has been previously shown that transfer of CD25+ FoxP3+ Treg cells alleviates colitis^27–31^. Since we detected increased presence of FoxP3+ cells in the mucosa of *IκBα* ^*IEC KO*^ mice that were protected from colitis, we hypothesized that if epithelial NF-κB prevents runaway inflammation through recruitment of Treg cells, transfer of an equivalent number of Treg cells either from *IκBα* ^*IEC KO*^ or from Wt mice into DSS-treated animals would alleviate colitis to the same extent. However, Treg cells are functionally heterogeneous, and exposure to distinct tissue environments can profoundly alter their protective capacity^27,32^.

**Fig. 5:**
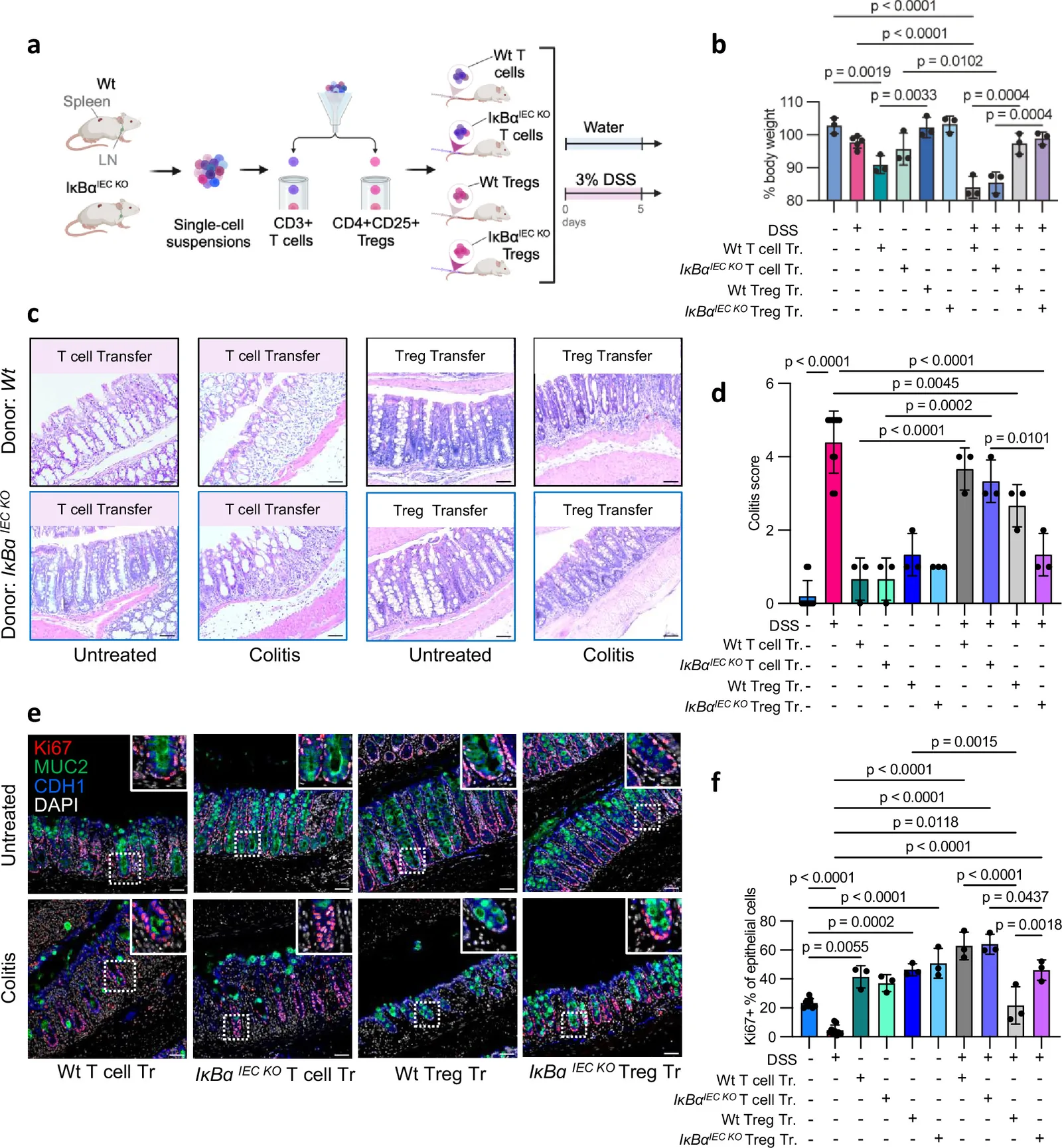
Epithelial NF-κB shapes a protective Treg phenotype that limits inflammation. **a** Diagram of adoptive transfer. Cells from donor mice (genotype shown) were derived from spleen and lymph nodes. Created in BioRender. Kolesnichenko, M. (2026) https://BioRender.com/97ildob. **b** Body weight quantitation. The initial weight of untreated mice was set at 100%. Final weight shown after 5 days. Analysis: one-way ANOVA with Tukey’s correction. n (biological replicates) shown as individual points on the graph. *n* = 3 for all apart from DSS treated, where *n* = 5. Mean and SD shown. **c** H&E after T cell (2 left columns) or Treg cells (2 right columns) transfer into Wt mice. Top row: Wt donor mice. Bottom row: *IκBα* ^*IEC KO*^ donor mice. Scale: 50 µm. **d** Colitis score on mice from (**c**). The mouse number used for the experiment is shown. Analysis as in (**b**). *n* = 3 mice per group as in d. and *n* = 5 for the DSS group. Mean and SD shown. **e** IF against CDH1, MUC2 (goblet cells), Ki67+ (proliferating stem cells) and DAPI. The upper right corner shows a zoom in of the outlined crypt. Scale: 50 μm. **f** Quantitation from e. *n* = 3 (mice) per group. And *n* = 5 for controls. Mean and SD shown. Analysis as in (**b**). Source data are provided as a Source Data file.

CD4+CD25+ Treg cells or CD3+ lymphocytes were isolated from the donor Wt or the *IκBα* ^*IEC KO*^ mice for adoptive transfer (Supplementary Fig. 6a, b). Wt recipients were either treated with DSS or left untreated and received a single infusion of either CD3+ or CD4+CD25+ cells (Fig. 5a). We verified that equivalent numbers of Treg cells migrated to the colon by flow cytometry (Supplementary Fig. 6c, d).

Mice that received only the CD3+ cells experienced short-term weight loss, which was mostly regained in mice that were not also treated with DSS (Supplementary Fig. 6e). Mice that additionally received DSS experienced a second DSS-colitis-mediated weight loss, occurring from day 4 onwards. Remarkably, up to day 4, transfer of CD3+ cells from *IκBα* ^*IEC KO*^ but not from Wt mice, led to significantly less weight loss, even in the presence of DSS. No significant difference was observed in colon lengths or in the colitis score between the two groups of DSS-treated recipients from day 4 onwards (Fig. 5b–d and Supplementary Fig. 6g, h). Previous studies demonstrated the contribution of T lymphocytes to the proliferation of intestinal stem cells^33^. In DSS-treated recipients, we detected significant expansion of proliferating Ki67+ cells in the crypt (Fig. 5e, f). Interestingly, T cell transfer alone also resulted in increased differentiation of stem cells to goblet cells (Fig. 5e and Supplementary Fig. 6i), indicating that, in addition to promoting proliferation, T lymphocyte-IEC crosstalk skews lineage commitment to secretory precursors.

Mice that received Treg cell transfer were protected from colitis (Fig. 5c–f and Supplementary Fig. 6f), evidenced by less weight loss, lower colitis score, and a significant increase in proliferating stem cells in crypts. Remarkably, mice with transferred Treg cells from *IκBα* ^*IEC KO*^ were even better protected from colitis than those receiving Treg cells from Wt donors (Fig. 5c–f and Supplementary Fig. 6g–i). This was reflected in retention of the normal weight after DSS treatment, lack of colon shortening, and, unlike for mice receiving Treg cells from Wt donors, by the absence of diarrhea (Supplementary Fig. 6e–i). We also detected a significant increase in proliferating stem cells in the crypt even after DSS treatment (Fig. 5e, f) and overall retention of a functional epithelial barrier (Fig. 5c–e). In summary, our data showed that IEC through NF-κB promotes recruitment of distinct types of tolerogenic T lymphocytes in the gut, including Treg cells, which in turn increase the proliferative capacity of crypt stem cells and protect mice from colitis. Since the same number of transferred Treg cells derived from *IκBα* ^*IEC KO*^ mice showed increased capacity to restrain colitis, we hypothesized that epithelial NF-κB shapes Treg cell phenotype, which is associated with enhanced protective capacity.

### Epithelial secretome promotes a metabolically distinct Treg cell phenotype

**Fig. 6:**
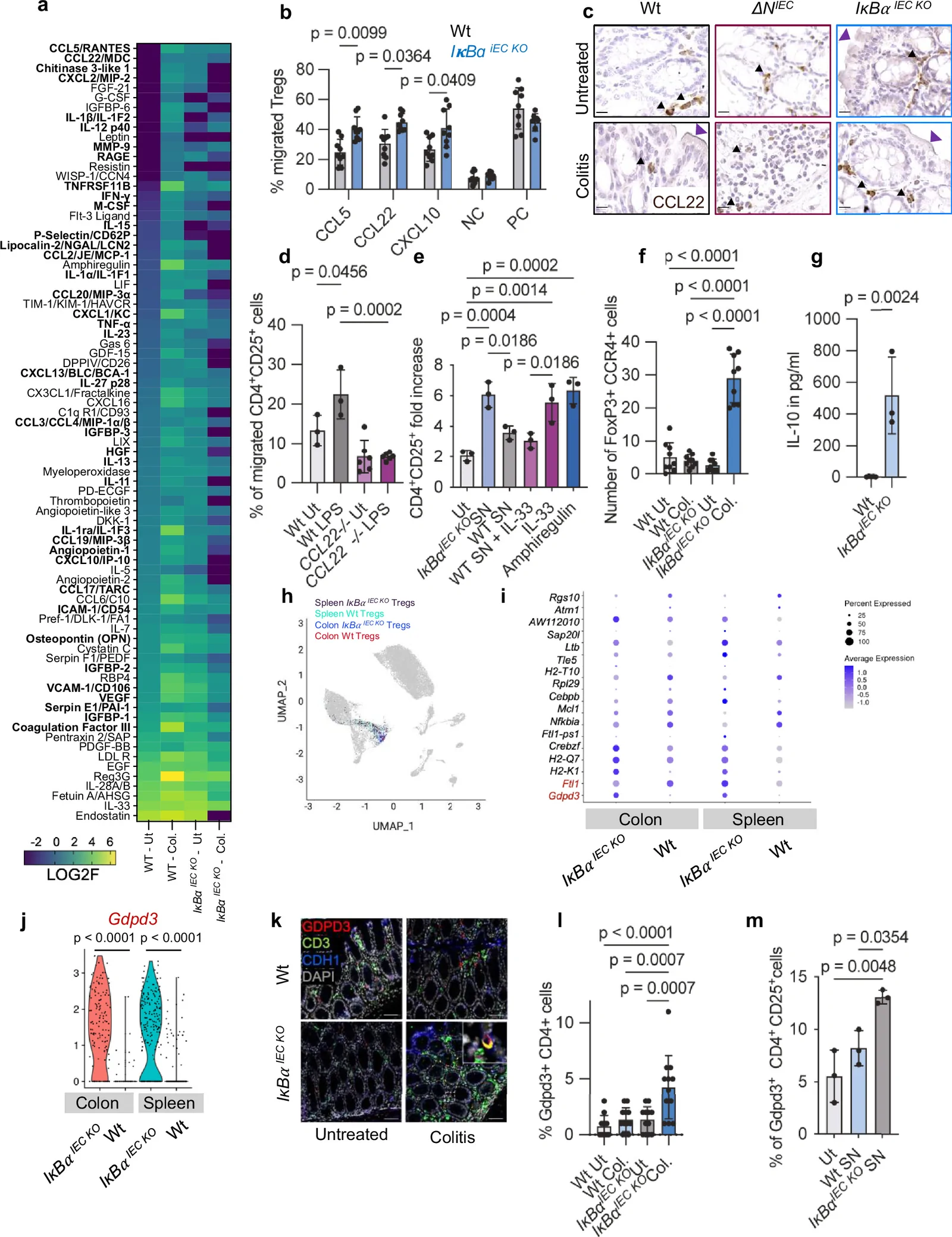
Epithelial secretome promotes metabolic rewiring of Tregs. **a** Medium from freshly isolated organoids analysed by proteome array. *n* = 4 mice per group, pooled. Analysis: one-way ANOVA with Tukey’s correction. In bold: known NF-κB target genes. **b** Treg cell migration transwell assay: isolated MLN/ spleen primary Treg cells from Wt mice incubated with factors shown. NC (negative control – medium alone) or PC (positive control – Fetal bovine serum). Analysis: one-way ANOVA; Bonferroni’s multiple comparison test. Mean and SD shown. *n* = 9. **c** IHC against CCL22 (*n* = 5 mice). Black triangles: CCL22+ immune cells. Purple triangles: CCL22+ epithelial cells. Scale: 10 μm. **d** Primary Treg cells from Wt mice cultured with organoid conditioned medium from Wt vs CCL22^−/−^ mice (*n* = 3 mice), treated with LPS (25 μg/ml LPS over 48 h) or left untreated. Treg cell migration quantified via Transwell migration assay. Analysis: one-way ANOVA. Mean and SD shown. **e** Treg cell co-culture with conditioned organoid medium (SN), 30 Vol.-% and/or + 30 ng/mL murine IL-33, CM + 30 ng/mL IL-33 or CM + 100 ng/mL Amphiregulin. The fold change increase of expanded Treg cells on day 3 was measured in relation to the initial cell count. *n* = 3 mice. Analysis: as in a. Mean and SD shown. **f** Quantitation of CCR4+ and FoxP3+ cells from IF per image. *n* = 9. Mean and SD shown. **g** Isolated Wt (*n* = 5) and *IκBα*^*IEC KO*^ (*n* = 3) CD4 + CD25+ Treg cells cultured for 6 h in medium supplemented with 50 ng/mL PMA and 1 µg/mL ionomycin. Supernatants analysed by ELISA. Analysis: unpaired t-test, two-sided. Mean and SD shown. **h** scRNA of colonic versus splenic immune cells from Wt and *IκBα*^*IEC KO*^ mice. **i** Genes with significant changes in expression between colonic or splenic Treg cells in Wt versus *IκBα*^*IEC KO*^ mice. **j** Differential expression of *Gdpd3* and *Ftl1* in spleen and colon Treg cells. Analysis: two-sided Wilcoxon rank-sum test **k** Colonic IF against GDPD3, CD4 and CDH1. Scale: 50 μm. *n* = 12. Zoom: double positive cells. **l** Quantitation of GDPD3+CD4+ cells per image. Analysis as in (**a**). Mean and SD shown. **m** Primary Treg cells from Wt mice cultured with organoid SN (supernatants). *n* = 3 mice. GDPD3+ cells visualized by IF. Analysis: as in (**a**). Mean and SD shown. Source data are provided as a Source Data file.

Analyses of the scRNA-seq data (Fig. 1j) together with bulk RNA-seq of isolated crypts (Fig. 3b, d) converged on a set of genes whose products facilitate paracrine interactions with T lymphocytes. Therefore, to explore which factors are secreted by epithelium with activated NF-κB and contribute to enhanced protection from colitis, we isolated crypts from *IκBα* ^*IEC KO*^ mice and from their Wt littermates (Fig. 6a). Crypts were cultured for two days to exclude immune and stromal cells, and the medium was collected for secretome analysis by a proteome array. As expected, DSS treatment of mice led to a sharp increase in secreted factors, many of which were coded by the NF-κB target genes, including interferon-gamma (IFNγ), CXCL1, and Coagulation factor III (F3) (Fig. 6a and Supplementary Data 4), consistent with injury response. Crypts from untreated *IκBα* ^*IEC KO*^ mice exhibited increased secretion of multiple factors encoded by NF-κB target genes, including CCL22. Many of these factors were already upregulated under homeostatic conditions in the IEC of *IκBα* ^*IEC KO*^ mice; however, upon DSS treatment, their expression was unexpectedly reduced, suggesting that in response to additional inflammatory stimuli, previously activated NF-κB could act as an inhibitor for a subset of genes.

We next analysed pathways that are regulated by NF-κB in colitis (Supplementary Fig. 7a and Supplementary Data 5). Inflammatory cytokine secretion, IL6/JAK-STAT, as well as IFNγ and IFNα pathways, were mediated by NF-κB (Supplementary Fig. 7a). Under “inflammatory response”, several cytokines known to recruit T lymphocytes and Treg cells were secreted by IEC in an NF-κB-dependent manner, including CCL22 and CCL5 (Fig. 6a). These findings suggest that protection is mediated not by a single factor, but by a coordinated NF-κB-dependent epithelial secretome induced before full-blown colitis.

To determine how individual chemokines regulated by NF-κB in IEC promote Treg cell recruitment, we selected those that showed the highest induction both in scRNA-seq analysis of NF-κB target genes (Fig. 1j) and in proteome analysis of secreted factors (Fig. 6a). We therefore selected chemokines CCL22 and CCL5 for further studies. Both CCL22 and CCL5 promoted robust recruitment of primary Treg cells using a transwell migration assay (Fig. 6b). Previous studies by David Anz and team showed that CCL22 derived from dendritic cells recruits Treg cells to alleviate DSS-induced colitis^34^. Immunohistochemistry (IHC) staining against CCL22 revealed the presence of the chemokine also in intestinal epithelium, especially in DSS-treated mice, but also in *IκBα* ^*IEC KO*^ mice in homeostasis (Fig. 6c), confirming our previous observations that IECs produce and secrete CCL22.

To functionally test the requirement for epithelial CCL22 for NF-κB-mediated recruitment of Treg cells, we cultured epithelial organoids from *Ccl22*^−/−^ mice and stimulated them with LPS, which we and others have previously shown to robustly activate NF-κB. We then performed transwell migration assays using primary Treg cells exposed to organoid– cultured medium from Wt or Ccl22^−/−^ organoids, which were either untreated or stimulated with LPS (Fig. 6d). While LPS stimulation of Wt organoids strongly promoted Treg cell migration, this effect was completely absent when Treg cells were exposed to supernatants from *Ccl22*^−/−^ organoids demonstrating that CCL22 is a key mediator of NF-κB–dependent Treg cells recruitment.

The proteome screen identified factors that are known to regulate recruitment, but also some that have been shown to boost Treg cell proliferation and activation. Treatment of primary Treg cells with organoid medium from *IκBα* ^*IEC KO*^ mice resulted in increased proliferation and boosted expression of the C–C chemokine Receptor type 4, CCR4 (Fig. 6e and Supplementary Fig. 7b), which is the only known receptor for CCL22, and is associated with effector but not naive Treg cells^35^. Consistent with this observation, colonic Treg cells from *IκBα* ^*IEC KO*^ mice displayed upregulated CCR4 (Fig. 6f).

Of the factors secreted in an NF-κB-dependent manner, IL-33 and amphiregulin were previously shown to regulate Treg cell proliferation or activation^36,37^, and we therefore selected these for further analyses. Treatment of primary Treg cells with either IL-33 or amphiregulin increased their proliferation, survival, and expression of CCR4 (Fig. 6e and Supplementary Fig. 7b). To determine whether Treg cells from *IκBα* ^*IEC KO*^ mice were also functionally more suppressive, we measured secretion of IL-10 - an essential cytokine for suppression of Th-17 mediated inflammation^38^. Isolated colonic Treg cells of *IκBα*^*IEC KO*^ mice and Wt littermates were briefly taken into culture without expansion, and the supernatant from the medium was analysed for IL-10 by ELISA. Treg cells from *IκBα*^*IEC KO*^ mice produced over 100-fold more IL-10 than Treg cells from the Wt littermates (Fig. 6g). Of note, the enhanced suppressive phenotype of Treg cells from *IκBα*^*IEC KO*^ mice was lost upon four-day removal from their tissue environment. Consequently, although these Treg cells retained the capacity to suppress the proliferation of recipient CD4⁺ T cells in vitro, the difference relative to Treg cells from Wt mice was no longer detectable (Supplementary Fig. 7c). Together, these findings indicate that epithelial NF-κB shapes colonic Treg cell responses through two parallel, yet mechanistically distinct, mechanisms. IEC NF-κB promotes Treg cells recruitment via CCL22 and is associated with a phenotypic adaptation that enhances suppressive function but requires continuous exposure to the intestinal epithelial microenvironment.

Finally, to shed light on the nature of the Treg cell functional state in *IκBα* ^*IEC KO*^ mice and to address which aspects of the phenotype are confined to the colon and which are systemic, we isolated immune cells from the colon and from the spleen for scRNA-seq (Fig. 6h). Genes whose expression was regulated in colonic Treg cells only but not systemically contained six factors (Supplementary Fig. 7d), including Cyclin dependent kinase 8 (*Cdk8*), whose pharmaceutical suppression was previously shown to generate Treg cells in a TGF-β- independent manner^39^ and also Basic Transcription Factor 3 (*Btf3*).

Splenic Treg cells from *IκBα*^*IEC KO*^ mice showed increased expression of MHC II factors (Fig. 6i), suggesting acquisition of an activated, antigen-presenting competent regulatory state that extends beyond the intestinal tissue. Expression of two genes regulating metabolic processes, Glycerophosphodiester Phosphodiesterase Domain Containing 3 (*Gdpd3*) and Ferritin Light Chain 1 (*Ftl1*), showed the strongest specificity for *IκBα*^*IEC KO*^ but not Wt Treg cells (Fig. 6j). Interestingly, a subset of splenic Treg cells was also enriched for *Gdpd3* (Fig. 6j), raising the possibility that these were programmed in the gut and subsequently recirculated systemically. Indeed, immunophenotyping of splenic Treg cells supported this observation: Treg cells in the spleen of *IκBα* ^*IEC KO*^ mice were enriched for CCR9 (Supplementary Fig. 4j), a key gut-homing marker^40,41^. This was further validated by analysing Treg cells with high versus low *Gdpd3* expression in the spleen, irrespective of the genotype (Supplementary Fig. 7e): *Ccr9* was enriched in *Gdpd3* high but not in *Gdpd3* low Treg cells. A similar enrichment could be observed for another gut-homing marker, *Gpr15* (Supplementary Fig. 7e). To determine whether CD11c+ cells contribute to shaping the Treg cell phenotype in *IκBα* ^*IEC KO*^ mice, we isolated primary CD11c+ cells and CD4+CD25+ Treg cells and co-cultured them in the presence or absence of organoid-conditioned medium (supernatant; SN) from WT or *IκBα* ^*IEC KO*^ organoids (Supplementary Fig. 7f, g). Previous studies and our own data suggest that strong in vitro TCR/CD3/CD28 stimulation can override tissue-imprinted Treg cell phenotypes during expansion^42,43^. Accordingly, these experiments were performed without CD3/CD28 stimulation. Under these conditions, Treg cells cultured alone did not proliferate, whereas co-culture with CD11c+ cells promoted Treg cell expansion and increased the frequencies of ICOS+ and CCR4+ Treg cells (Supplementary Fig. 7f, g). These effects were not further enhanced by the addition of organoid SN.

We next assessed whether the epithelial secretome could enhance an IL-10-associated suppressive function. Organoid SN contained no detectable IL-10 before its addition to the cultures (Fig. 6a). However, SN from *IκBα* ^*IEC KO*^ organoids induced the greatest IL-10 production in Treg cell cultures, and this response was not further increased by the addition of CD11c+ cells (Supplementary Fig. 7h). Together, these findings indicate that CD11c+ cells promote Treg cell expansion and activation, whereas the epithelial NF-κB-dependent secretome can directly enhance IL-10 production by Treg cells independently of CD11c+ cells.

We next sought to validate GDPD3 as a marker of reprogrammed Treg cells in *IκBα* ^*IEC KO*^ mice by immunofluorescence (Fig. 6k, l). GDPD3 was enriched in Treg cells of *IκBα* ^*IEC KO*^ but not Wt mice. To determine if exposure to epithelium with activated NF-κB was sufficient to induce GDPD3 production, we cultured primary Treg cells from Wt mice with organoid-conditioned medium from either the Wt or the *IκBα* ^*IEC KO*^ mice (Fig. 6m). Only the latter promoted an increase in GDPD3 expression in Treg cells. In summary, IEC NF-κB is associated with tissue-specific functional state in Treg cells, coupling local epithelial cues to systemic regulatory adaptation.

### Deregulated rather than activated epithelial NF-κB in ulcerative colitis is associated with impaired acute stress responses

**Fig. 7:**
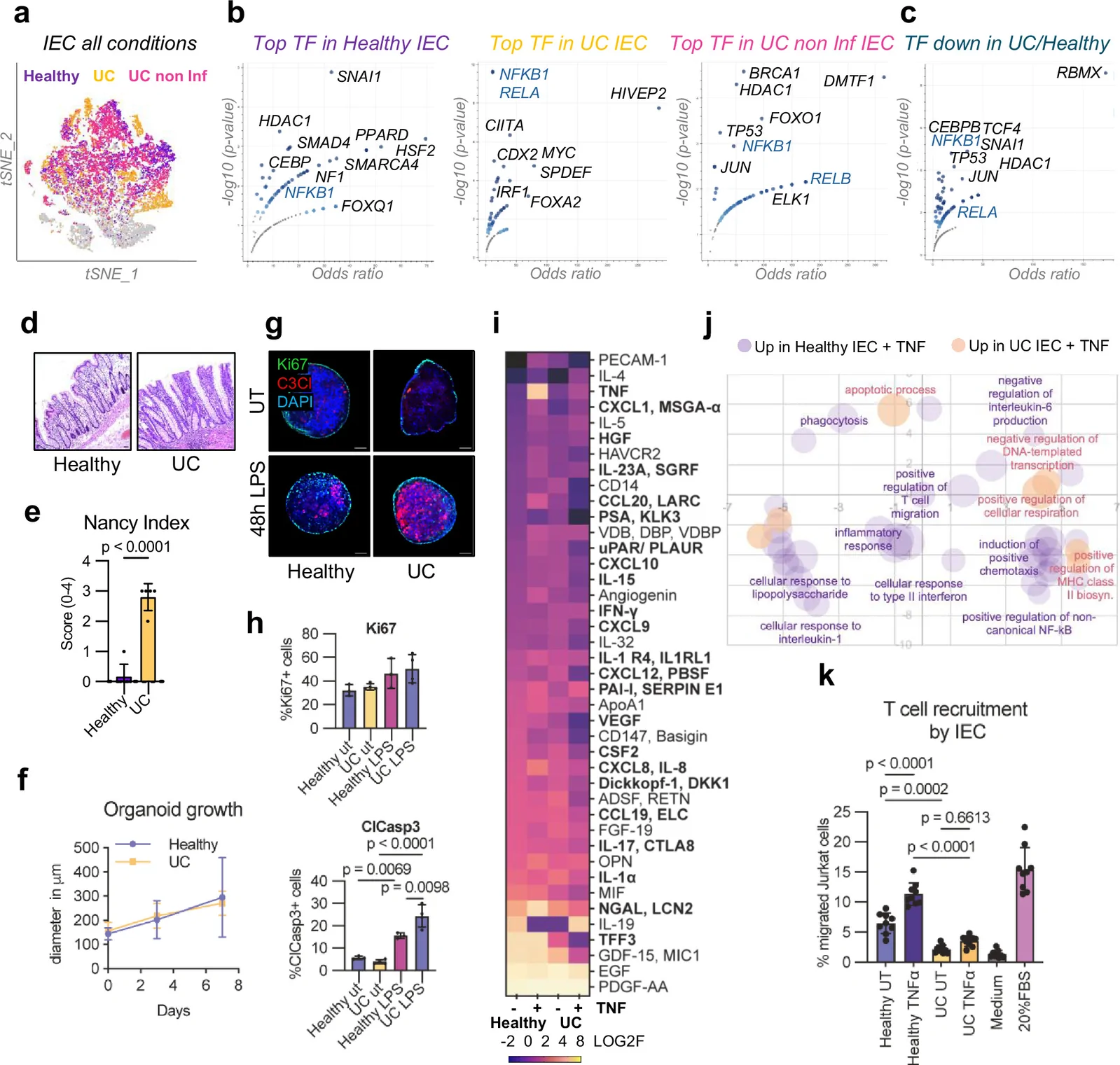
Deregulated NF-κB in ulcerative colitis patients prevents protective acute epithelial response. **a** scRNA dataset (GSE116222)^18^ is based on samples from inflamed and non-inflamed colon regions of UC patients, as well as those from healthy controls. Visualization of IEC cells across the three conditions. **b** TRRUST analysis of top upregulated TF networks across the three conditions (Healthy, UC and UC NI (non-inflamed)) in IEC presented as a volcano plot. Text in blue: factors belonging to the NF-κB axis. A Gene-set enrichment was performed using Enrichr. Enrichment significance was assessed using Fisher’s exact test, and enrichment magnitude was represented by the odds ratio. Volcano plots generated with the Enrichr Appyter display odds ratio versus −log_10_(*P* value). **c** TRRUST analysis of downregulated TF networks in UC-inflamed colon IEC versus healthy IEC. Text in blue: factors belonging to the NF-κB axis. Analysis as in (**b**). **d** H&E of colon sections from healthy and UC patients. Scale: 50 μm. **e** Colitis score (Nancy index) from (**d**). The patient number is shown in the graph. Analysis: Two-tailed *t*-test. Mean and SD shown. **f** Colon-derived organoids were derived from colon biopsies of UC patients or non-IBD controls, with at least *n* = 4 patients per group. Growth of colon organoids was measured over several days. Mean and SD shown. Analysis: *t-*test, two-tailed, unpaired. **g** At least 10 colon organoids per patient from healthy (*n* = 3 persons) and UC colon-derived organoids (*n* = 4 patients) treated with LPS over 48 h and stained with antibodies as indicated. Scale: 50 μm. **h** Quantification from g. Top panel: Ki67 (not significant). Lower panel: cleaved caspase 3. Analysis: One-way ANOVA with Tukey’s correction. Mean and SD shown. **i** Colon organoids were treated with TNF (TNF was removed, and the medium was changed) or left untreated. Medium from colon-derived organoids was analysed by proteome array, and the presence of secreted factors quantitated from bulk (*n* = 4) samples per group and *n* = 2 technical replicates. Significance determined by one-way ANOVA with Tukey’s correction. Only significant results are shown. **j** Analysis of proteome data by DAVID v2023q4 followed by REVIGO for visualization of GO terms on semantic space. Bubble size indicates prevalence. Color: orange—terms enriched in medium of UC colon-derived organoids treated with TNF, purple—terms enriched in medium of healthy colon-derived organoids treated with TNF. SD and mean shown. Analysis: Welch’s *t*-test, two-sided – no significance. **k** Transwell recruitment with Jurkat cells incubated in medium from colon-derived organoids from UC patients or healthy controls. Analysis: one-way ANOVA with Tukey’s correction. *n* = 4 for healthy samples and *n* = 6 for UC samples (pooled). Individual points indicate technical replicates. Mean and SD shown. Source data are provided as a Source Data file.

Previous studies showed that in UC and CD, NF-κB is activated both in immune and in epithelial cells^14^; however, it is not clear which NF-κB transcriptomes are expressed and whether these are protective. We analysed previously published scRNA dataset (GSE116222)^18^ based on samples from inflamed and non-inflamed colon regions of UC patients, as well as those of healthy controls, to visualize IEC transcriptomes across the three conditions (Fig. 7a). Characterization of dominant TF networks revealed a dramatic shift from programs balancing epithelial-mesenchymal transition in healthy IEC to immunomodulatory programs mediated by canonical RELA/NFKB1 (p65/p50) and the Class II, major histocompatibility complex, Trans-Activator (CIITA) in UC (Fig. 7b). Importantly, in inflamed colon of UC patients, we detected *deregulated*, rather than activated NF-κB: whereas many NF-κB target genes showed increased expression, others, including a subset regulating metabolic processes, Toll-like Receptor activation, and stem-cell maintenance showed significant decrease in expression (Fig. 7c). Accordingly, TRRUST analysis showed RELA and NFKB1 as some of the top repressed TF networks (Fig. 7c). Together, these findings indicate that NF-κB remains central to UC pathology, yet disease is marked not by global NF-κB activation but by a deregulated and imbalanced NF-κB transcriptional output.

To assess whether IEC retain the capacity to engage the protective NF-κB signaling observed in *IκBα* ^*IEC KO*^ mice when removed from the inflammatory milieu, we established organoids from colon biopsies from UC patients and healthy controls (Fig. 7d, e). Organoids from both groups exhibited comparable growth kinetics during passaging (Fig. 7f). To test for IEC-intrinsic functional defects, organoids were stimulated with LPS, a known activator of NF-κB. While no differences in proliferation were observed, UC-derived organoids displayed increased susceptibility to apoptosis (Fig. 7g, h). These findings indicate that, despite evidence of NF-κB activation in UC, epithelial NF-κB signaling fails to engage protective programs in the disease context.

To determine if the epithelium from UC patients is capable of appropriately reacting to additional acute stress and activating protective NF-κB response to promote recruitment of CD3+ lymphocytes, we stimulated organoids with TNF and collected medium for secretome analysis after TNF removal and additional culture time. In organoids derived from healthy colons, TNF stimulation led to a sharp increase in production of acute pro-inflammatory mediators, including TNF, Lipocalin-2 (LCN2), and CXCL1 (Fig. 7i). Although many of these were already secreted by organoids from UC patients, additional stimulation did not further increase their production and, in some cases, resulted instead in inhibition. We next analysed which TF networks are activated by TNF in healthy versus UC colon (Fig. 7c). Strikingly, TNF treatment of UC organoids reduced the number of activated TF networks, and most notably of RELA, indicating that at least for some genes, constitutive NF-κB in IEC functions as a repressor, preventing protective acute response.

Analysis of GO terms from TNF-stimulated organoids from healthy colon revealed enrichment for processes mediating T cell recruitment, positive activation of NF-κB, and response to IL1, as well as type II interferon signaling (Fig. 7j and Supplementary Data 6). On the contrary, the few processes upregulated in TNF-stimulated UC organoids were enriched for genes regulating MHC class II, repression of transcription, and intrinsic apoptosis. To determine if IEC secretome from UC organoids treated with TNF would induce T lymphocyte recruitment, we cultured Jurkat cells in medium from organoids treated as above. Robust induction of T cell recruitment was only observed when organoids from healthy colon were exposed to exogenous TNF (Fig. 7k), confirming our findings from murine models (Figs. 2 and 4) that acute and transient NF-κB initiated by epithelium is necessary for the correct sequence of leukocyte recruitment and protection from persistent inflammation.

**Fig. 8:**
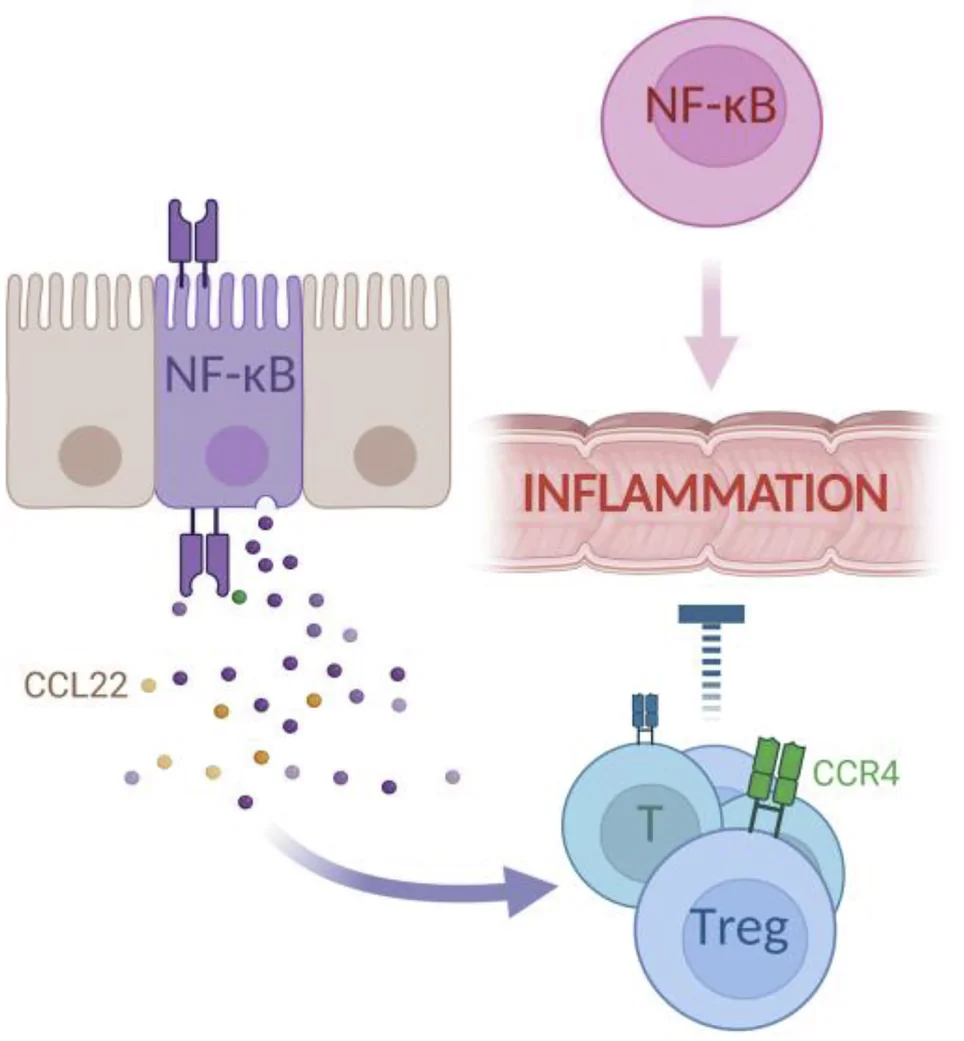
Early pro-inflammatory IEC secretome mediated by NF-κB recruits tolerogenic T lymphocytes and preventsuncontained colitis. Proposed model: Immune cells with activated NF-κB (magenta) are pro-inflammatory and fuel inflammation. IEC with activated NF-κB (purple) display MHC II. Secretion of CCL22 (yellow) and other factors from IEC (purple circles) recruits and programs T cells including Treg cells (blue) that promote IEC proliferation and thwart runaway colitis. Created in BioRender. Kolesnichenko, M. (2026) https://BioRender.com/24z22ck.

Taken together, our data support a model in which IECs act as early responders to acute stress and require transient NF-κB activation to initiate recruitment of T lymphocytes that promote epithelial regeneration and limit persistent inflammation (Fig. 8).

## Discussion

Beyond nutrient absorption, intestinal epithelial cells (IEC) act as both a physical barrier and a sensor of microbial and inflammatory cues. Here, we show that IECs act as active immune sentinels and, through NF-κB activation, promote recruitment of Treg cells. Moreover, IEC NF-κB shapes the mucosal immune compartment, resulting in the accumulation of highly suppressive Treg cells that prevent progression from acute injury to chronic colitis. Using genetic models that directly modulate NF-κB transcriptional activity in IEC and a reporter that visualizes active NF-κB at single-cell resolution, we provide mechanistic insight into the long-standing paradox of epithelial NF-κB signaling by showing that the timing of epithelial-intrinsic NF-κB activation determines its protective function.

We show that the NF-κB–driven epithelial secretome is required for recruitment of T lymphocytes and induction of protective Treg cells. Elegant early studies demonstrated that Treg cells promote resolution and recovery^29,30,44^. Nonetheless, the role of IEC in recruiting Treg cells was largely unknown^45^. Our data demonstrate that epithelial NF-κB induces a coordinated array of chemokines and cytokines—including CCL22, CCL5, IL-33 and amphiregulin. This multifactorial secretome acts prior to full-blown inflammation and restrains excessive innate immune responses.

Beyond recruitment, epithelial NF-κB signals also shape the functional state of Treg cells. Treg cells in vitro exposed to the epithelial secretome from *IκBα* ^*IEC KO*^ mice displayed increased CCR4 expression, elevated IL-10 production, and in vivo conferred stronger protection in adoptive transfer experiments than Wt Treg cells. Importantly, these data indicate that effective resolution of colitis depends not merely on Treg cell abundance, but critically on their functional state.

Treg cells exposed to epithelial NF-κB-dependent signals showed induction of metabolic regulators, including GDPD3. GDPD3 has been proposed as a marker of memory T cells^46^ and shown to be required for survival of myelogenous leukemia stem cells^47^, suggesting that epithelial NF-κB cues are associated with acquisition of a tissue-adapted Treg cell state. GDPD3 was also detected in a subset of splenic Treg cells, suggesting that aspects of epithelial-induced Treg cell conditioning may extend beyond the intestinal tissue. Indeed, we detected a significant increase in CCR9+ Treg cells in the spleen of *IκBα* ^*IEC KO*^ mice, suggesting that Treg cell trafficking could contribute to extraintestinal expansion of the suppressive phenotype. Nonetheless, direct lineage-tracing studies will be required to determine whether this reflects recirculation of gut-conditioned Treg cells or systemic signals downstream of epithelial NF-κB activation.

Our results also refine the understanding of epithelial NF-κB signaling in human UC. Rather than uniform activation, IEC in UC displayed a deregulated NF-κB transcriptome and secretome. UC-derived organoids were more susceptible to apoptosis and failed to mount an appropriate acute secretory response to inflammatory stimulation compared with organoids from healthy tissue. These observations suggest that chronic disease is associated not simply with increased NF-κB activity but with defective engagement of protective NF-κB-dependent epithelial programs. This framework provides a mechanistic explanation for the paradoxical observation that Treg cell numbers can be increased in active UC while inflammation persists. In the absence of properly timed epithelial NF-κB–dependent cues, Treg cells may accumulate but remain functionally insufficient to support epithelial repair and barrier restoration.

Several limitations should be noted. Some in vitro experiments relied on spleen- or lymph node-derived Treg cells to obtain sufficient numbers of primary cells, and direct tracking of gut-conditioned Treg cells was not performed. Future studies will be needed to define how secreted factors and metabolic cues cooperate to shape epithelial - immune crosstalk during intestinal inflammation.

Together, our findings identify epithelial NF-κB as a central initiator of a protective mucosal program that recruits Treg cells and contributes to their protective functional state to restrain inflammation and support tissue repair (Fig. 8). Although many epithelial NF-κB–regulated factors are traditionally considered pro-inflammatory, our data show that in the appropriate context, they form a protective, immune-instructive secretome required for barrier defense and recovery. Preserving or selectively inducing this epithelial NF-κB program may therefore represent an important strategy for promoting durable resolution of intestinal inflammation.

## Methods

### Human samples and data

Written informed consent was obtained from all study participants. The study was approved by the Charité - Universitätsmedizin Berlin Research Ethics Committee (EA2_046_23). Colonic biopsies were collected from participants with and without UC who had a prearranged colonoscopy. Baseline patient and disease characteristics and medication history were collected (details in Supplementary Data 7). UC patient biopsies were taken during colonoscopy from the macroscopically inflamed colon. Healthy control samples were taken from patients without gastrointestinal symptoms undergoing endoscopic investigation for polyps or other non-IBD conditions. Patient biopsies were washed in PBS and immediately processed to generate organoids. Additional biopsies were fixed in 10% neutral buffered formalin (Sigma-Aldrich Cat. HT501128) at 20 °C for 24 h for histology processing. For immunofluorescence analysis, samples were obtained from an independent cohort of patients with and without UC who underwent resection at the Charité Universitätsmedizin Berlin. Paraffin samples were obtained from the Pathology Department patient sample collections. Equal representation of male and female patients over 18 and under 80 years of age was considered for all experiments on patient samples.

### Mice

*Ola 129S villinCRE x iκB alpha fl/fl igk-EGFP-3/OLA* (text: *IκBα* ^*IEC KO*^ and *IκBα* ^*IEC KO*^ *-EGFP*), *villinCRE x loxP (loxPdeltaN) igk-EGFP −3/OLA* (text: *ΔN*^*IEC*^ and *ΔN*^*IEC*^ *EGFP*) Ccl22tm1(KOMP)Vlcg, and *B6-Tg(k-EGFP) 3Pt/Rsu* (text: *κEGFP*) were bred in the lab, from original strains^17,48^, genotyped and phenotyped to confirm specific insertions and deletions. All mice used in DSS experiments were between 8–26 weeks of age. All mice were bred and housed under specific pathogen-free conditions in a standard 12-h light/12-h dark cycle at 20 °C with a humidity range of 30–70%, at the Max Delbrück Center for Molecular Medicine and Charité FEM-Buch in Berlin-Buch. Mice were fed standard rodent chow and water ad libitum. Age-matched male siblings were used for all experiments, apart from adoptive transfer, in vitro organoid-based assays and immune phenotyping, where both males and females were used. DSS treatment: Animals were treated with DSS (MP Biomedicals, Colitis Grade MFCD00081551) in drinking water at concentrations indicated in the manuscript. Weight and drinking amount were controlled to ensure equivalent consumption of water vs. DSS in water. Mice were observed daily, and their weight was recorded during the experiment. Colitis score was determined as discussed elsewhere^49^. Mice were sacrificed by cervical dislocation.

All animal work was approved by the Landesamt für Gesundheit und Soziales Berlin (LaGeSo), ethics approval: G0092-18 and G-0175-23.

### Mouse tissue processing and cell isolation

Mice were sacrificed, and tissues were immediately collected and processed as described previously^10,17^. In brief, the colon and small intestine were cut longitudinally and washed with ice-cold PBS. The distal colon and terminal ileum were processed into a “swiss roll” and fixed overnight in formalin. After overnight incubation in 70% EtOH, tissue was sequentially dehydrated using alcohol series: 30 min 80% in EtOH, 30 min 90% in EtOH, 1 h 96% in EtOH and 30 min 96% in EtOH, followed by 1 h 100% EtOH, 30 min 100% EtOH, 15 min 100% EtOH/Toluol or Roti Histol (Carl Roth Cat. 6640) (1:1) and again incubated for 1 h in Toluol or Roti Histol and for 30 min in fresh Toluol/ Roti Histol prior to fixation in paraffin (Paraplast, Leica) at 60 °C overnight. For paraffin embedding, tissue was transferred to the embedding mold, arranged planar to the bottom and left to solidify at room temperature. Tissue was stored at 4 °C. A microtome (Thermo Scientific, HM 355S) was used to achieve 4 μm-thick sections of the paraffin blocks. Sections were transferred to a microscope slide (SuperFrost) and dried at 37 °C for at least overnight. The proximal colon was snap-frozen in liquid nitrogen.

Tissue isolation for bulk RNA sequencing: proximal colon was washed as above and processed according to a previously described protocol for crypt preparation https://www.stemcell.com/technical-resources/educational-materials/how-to-isolate-mouse-intestinal-crypts.html and according to the organoid preparation protocol^50^, up until Matrigel embedding. Instead, crypts were collected and immediately lysed in TRIzol (ThermoFischer Scientific, Cat. 15596026). Extraction of RNA was performed according to the manufacturer’s instructions. RNA was precipitated and dissolved in water prior to further analysis (qPCR and RNAseq).

### Mouse organoid culture

Intestinal organoids were prepared as described: https://cdn.stemcell.com/media/files/pis/10000003509-PIS_05.pdf. Organoids were grown in Mouse Intesticult Medium (STEMCELL Technologies Cat. 06005) embedded in Corning Matrigel Matrix (Corning Cat. 356255). Organoids were treated for 48 h with 50 μg/ml LPS from *Salmonella enterica* serotype typhimurium (Sigma-Aldrich Cat. L6143). Immunofluorescence staining was performed as previously described^10^.

### Histology, immunofluorescence and immunohistochemistry staining, including Multiplex IF

H&E, immunofluorescence, and multiplex stainings were performed as described previously^10,24,51^. Colonic tissues for multiplex staining were incubated in 10% donkey serum (Sigma-Aldrich Cat. D9663 or S30-M or Abcam Cat. ab7475). They were stained with DAPI and secondary antibodies (Supplementary Data 8). For immunohistochemistry of CCL22, the formalin-fixed, paraffin-embedded murine tissues were deparaffinized and rehydrated. Antigen-demasking was performed by heating the sections in 10 mM Citrate-EDTA, pH 6.0, with 0.5% Tween-20, followed by endogenous peroxidase blocking in 3% H2O2, permeabilization with 1% Triton X-100 in PBS and blocking in 1% BSA with 0.1% Triton X-100. CCL22 was detected using a rabbit monoclonal anti-CCL22 antibody (Abcam, Cat. ab124768) at a dilution of 1:750, and the secondary antibody Peroxidase-conjugated AffiniPure Goat Anti-Rabbit IgG (H + L) (Jackson Immuno Research, Cat. 111-035-003) was added at a dilution of 1:200. The tissues were stained using 3,3-diaminobenzidine substrate solution and counterstained with hematoxylin. The sections were imaged with the 20x objective of the Pannoramic MIDI II (3DHISTECH).

### Colitis scoring

The mouse distal colon was processed as described above. H&E staining was performed as described previously^10^, and stained paraffin sections were scored according to the published guidelines for DSS colitis^49^ and the Nancy histological index for UC, respectively.

### Human organoid culture

Biopsies were obtained from UC and healthy colon as indicated above, and immediately processed to generate organoids in Human Intesticult medium (STEMCELL Technologies Cat. 06010) as previously described^52^ and according to the manufacturer’s protocol: https://cdn.stemcell.com/media/files/pis/10000003510-PIS_08.pdf. Organoids were cultured in Human Intesticult and embedded in Corning Matrigel Matrix (Corning Cat. 356255). Crypts were seeded in Matrigel at a ratio of 1:2. For 3 days, medium was substituted with 50 µg/mL Gentamicin (Thermo Fisher Scientific, Cat. 15710064), 100 µg/mL Primocin (InvivoGen, Cat. Ant-pm-2), 100 µg/mL Penicillin-Streptomycin (Fisher Scientific, Cat. 11548876), and 10 µM ROCK inhibitor (BD Biosciences, Cat. 562822). Medium without Primocin and ROCK inhibitor was changed every 2–3 days. Growth of organoids was quantified by bright-field microscopy. Organoids were treated for 24 h with 30 ng/mL human TNF (Peprotech Cat. 300-01 A), then washed 3 times with PBS. Fresh medium was added, followed by additional culture time for medium collection and secretome analysis.

### Flow Cytometry and EGFP+ cell isolation

The entire colon from EGFP+ mice was processed as described above, and epithelium was isolated as described elsewhere^53^. EGFP- mice were used as controls. Doublet exclusion was performed in both forward and side scatter, and EGFP+ cells were bulk sorted using a BD FACS Aria III Cell Sorter.

### Immune cell isolation for scRNA sequencing

Colon was removed, opened longitudinally and washed in ice-cold PBS. Dissociation of epithelium was performed by incubation on a shaker at 37 °C in HBSS solution (Ca^2+^ and Mg^2+^ free, Sigma-Aldrich) supplemented with 10 mM HEPES (Gibco) and 5 mM EDTA (Roboklon) twice for 15 min. After each step, samples were vortexed, and the epithelial fraction was discarded. Afterward, the tissue was minced and digested in HBSS (Sigma-Aldrich) supplemented with 4% FCS, Dispase (0,5 U/ml; Corning), Collagenase D (0,5 mg/ml; Roche) and DNase I (100 μg/ml; Sigma-Aldrich) at 37 °C twice for 20 min. Leukocytes were further enriched by Percoll gradient centrifugation (Sigma-Aldrich). After each step, samples were vortexed, filtered through a 70 µm cell strainer and kept on ice. Spleens were chopped, incubated in HBSS (Sigma-Aldrich) supplemented with 4% FCS, collagenase D (0,5 mg/ml; Roche) and DNase I (100 μg/ml; Sigma-Aldrich) for 30 min on a shaker at 37 °C and meshed through a 70 µm cell strainer. Red cell lysis was performed with Pharm Lyse (BD Biosciences).

Single-cell suspensions were stained with anti-CD45-APC-Cy7 (clone 30-F11, BD Biosciences) and DAPI (Biolegend) in ice-cold PBS. Before single-cell sequencing, immune cells from the colon and spleen were sort-purified as DAPI- CD45+ cells of Wt and *IκBα* ^*IEC KO*^ littermate controls by using a FACSAria cell sorter (BD Biosciences).

### Bulk RNAseq and data processing

Bulk RNAseq of mouse colon crypts under six different conditions was carried out using Illumina TruSeq strand-specific protocol in paired-end mode. The sample conditions consisted of a combination of two distinct NF-κB states (*ΔN*^*IEC*^ or Wt) and three different DSS treatments (untreated, colitis, recovery). Each condition consisted of 4 replicates; thus, a total of 24 samples were obtained. The raw sequences were processed and analysed using the PiGx RNA-seq pipeline^54^. The DNA (genome assembly version GRCm38), cDNA, and gene annotation (GTF) files for *Mus musculus* were downloaded from the ENSEMBL database (version 91)^55^. The raw reads were mapped to the genome using HISAT2^56^. Alignment-based expression at both gene/transcript level was quantified using the GenomicAlignments R package^57^ with “mode = Union; strandedness = Reverse”, where reads aligning to “exons” were counted and grouped by “gene_id”. Raw read counts were further normalized and converted to log-scale using the RUVseq R package^58^, where we used the “RUVs” method (*k* = 5), taking advantage of the replicate groupings based on the six sample conditions.

To measure the immune cell type activities in the samples, the Singscore R package^59^ was used to carry out single-sample gene set activity analysis based on the human immune cell type signatures annotated in the R package^59^, where gene signatures were transferred to mouse genes based on human orthologs. Heatmaps were visualized using the pheatmap R package [10.32614/CRAN.package.pheatmap].

Pathway perturbation analysis was carried out using the PROGENy R package^60^. All-vs-all differential expression analysis of each pair of conditions was carried out using DESeq2^61^. The Wald statistic values for each differential analysis were used as input to PROGENy to estimate the pathway perturbation scores.

### scRNA sequencing and data processing

For nuclei isolation, the tissue from whole gut was disrupted with 10-15 strokes of a plastic pestle in 500 µL of NP-40 lysis buffer (10 mM Tris-HCl (pH 7,4), 10 mM NaCl, 5.6 mM MgCl_2_, 0.1% NP-40, 1 mM DTT, Complete EDTA-free protease inhibitor, 2% BSA, 1 U/µl Protector RNase inhibitor) and incubated on ice for 5 min, with gentle pipette mixing after 2.5 min. The nuclei suspension was then filtered through a 70 µm strainer into a fresh 1.5 mL Eppendorf tube, and nuclei were sedimented by centrifugation at 500 x *g* for 5 min at 4 °C. After removing the supernatant, 1 mL of cold wash buffer (PBS, 1% BSA, 0.4 U/µl Takara RNAse inhibitor) was added without disturbing the pellet. Nuclei were incubated on ice for 5 min and then gently resuspended and sedimented again at 500 g for 5 min at 4 °C. Nuclei were then resuspended in 300–500 µL of cold wash buffer and filtered through a 40 µm Flowmi cell strainer. After adding DAPI (final concentration 0,1 µg/mL), nuclei were sorted on an Aria III instrument with a 100 µm nozzle in an Eppendorf tube containing 100 µL sort buffer (PBS, 2% BSA, 2.5 U/µl Protector RNAse inhibitor). Single-nucleus RNA sequencing libraries were prepared using the Chromium Single Cell 3’ GEX v3.1 assay (10X Genomics). In short, cell suspensions were encapsulated into Gel Beads in Emulsion (GEMs) using the Chromium Controller. Within each GEM, cell lysis and barcoded reverse transcription of RNA occurred, followed by cDNA amplification. The amplified cDNA underwent library construction via fragmentation, end-repair, A-tailing, adapter ligation, and index PCR. Final libraries were sequenced on an Illumina NovaSeq 6000 system to a minimum depth of 40.000 reads per cell.

Single-cell gene expression count tables were obtained using the CellRanger tool from 10x Genomics. To annotate EGFP+ cells, we searched for sequence fragments originating from the EGFP plasmid construct within the sequence reads. We used bbmap to align the raw sequence reads against the EGFP plasmid sequence with the parameters “k = 8 minid = 0.2 local = t”. Reads successfully aligned to the EGFP plasmid sequence were further analysed to pinpoint which cells they originated from by inspecting the cell barcodes. Those cells that matched the barcode were annotated as EGFP+ cells. If the cells were initially discarded by CellRanger, they were recruited back for further analysis.

Raw read counts were processed using the standard Seurat v5^62^ workflow, where the read counts were filtered by setting “min.cells = 3” and “min.features = 50”, normalized, and the top 2000 features were selected and transformed using the VST method and scaled.

The cell types were annotated using the SingleR and celldex R packages^63^ based on the reference cell type signatures “ImmGenData” from the mouse bulk expression data from the Immunologic Genome Project. Cells annotated as “Epithelial Cells” were further restricted to those positive for the Cdh1 expression. Marker detection in T-reg cells: cells annotated using the SingleR tool (using ImmGenData as reference) were labeled as Treg cells if they had positive FoxP3 expression. Differential gene expression between KO Treg cells and Wt Treg cells in both spleen and colon was carried out using Seurat’s built-in FindMarkers function with “min.pct = 1” and “logFoldChange = 0.25”.

### Cell-cell communication analysis

The scRNA dataset from the mouse colon (Fig. 1) was analysed by CellChat^26^ to assess the interactions between the epithelial and the immune cells. For this analysis, we focused on IEC positive for the *Cdh1* gene and negative for the *Ptprc* gene, and a subset of immune cell types which were frequently found in our dataset, namely: ‘B cells’, ‘DC’, ‘Macrophages’, ‘Mast cells’, ‘NKT’, ‘T cells’, and ‘Tgd’. We further annotated any cell that is positive for either *Il2ra* or *Foxp3* to be “Tregs”. A CellChat analysis was carried out for each sample separately.

### Analyses of published single-cell studies in UC cohorts

scRNA dataset (GSE116222)^18^ is based on samples from inflamed and non-inflamed colon regions of UC patients, as well as those from healthy controls. The raw data were downloaded using the NCBI SRA Toolkit from GEO. The Cell Ranger analysis pipeline (v7.1.0), was used for: (i) aligning the reads from the FASTQ files to the h38 (version: 2020-A) genome build; (ii) filtering and unmapped reads and non-cell droplets; (iii) generating cell x gene expression matrices. Data was clustered according to cell type (IEC) and analysed by TRRUST - a reference database of human transcriptional regulatory interactions^64^ for transcription factor activity, Database for Annotation, Visualization and Integrated Discovery (DAVID) https://david.ncifcrf.gov^65^ and significant GO terms were further reduced by Reduce and Visualize Gene Ontology (REVIGO).

### Gene Ontology analyses

Data were analysed using DAVID for enrichment of GO terms and annotations. NF-κB target genes were obtained from http://www.bu.edu/NF-κB/gene-resources/target-genes/ and https://www.grnpedia.org/trrust. Targets showing significant upregulation (*P* < 0.05) were used for Gene Ontology Analysis (GO Term_ BP_ ALL); Thresholds: count 2; EASE 0.1. *P* Value: Benjamini. Obtained GO terms were further reduced by REVIGO using parameters: SimRel, medium. Data was plotted using a bubble graph on semantic space, where proximity indicates closely related GO terms and bubble size indicates prevalence.

Where indicated, GO terms were obtained from the MSigDB_Hallmark_2020 gene set and visualized using Volcano plots, where each point represents a single gene set; the x-axis measures the odds ratio (0, inf) calculated for the gene set, while the y-axis gives the -log(*p*-value) of the gene set. TF contribution was obtained using established databases^19,64^ and was visualized using Volcano plots.

### qPCR

qPCR was performed as described previously^24^. At least two reference genes were used for normalization. Primers used are listed in Supplementary Data 8.

### Cell line transwell recruitment assay (mouse and human)

5×10^4^ cells in RPMI medium without additives were seeded in inserts with 8 µm pore size (Greiner Bio-One Cat. 662638) in a 24-well plate. Murine assay used EL4 cells in the upper chamber as well as supernatant from pooled Wt, *ΔN*^*IEC*^ or *IκBα* ^*IEC KO*^ organoids that were grown for 2 days or Mouse Intesticult ± 20% FBS (controls) in the bottom chamber. Organoids were derived from mice treated with ± 3% DSS for 5 days. Human assay used Jurkat cells in the upper chamber and supernatant of pooled healthy or UC organoids or Human Intesticult ± 20% FBS (controls) in the bottom chamber. Organoids were treated with 30 ng/mL TNF for 24 h or left untreated. All assays were performed in triplicate. After 1.5 h incubation at 37 °C, cells were fixed with 4% PFA in PBS for 15 min, stained with 1 μg/mL DAPI for 10 min and washed with PBS. Total and migrated cells were visualized with Zeiss LSM800 (EL4) or Zeiss Axio Observer 7 (Jurkat) and quantified with ImageJ 2.1 (Fiji). For the murine chemokine recruitment assays, EL4 cells were added to the upper chamber and either 100 ng/mL CCL22, CCL5 or CXCL10 (Fisher Scientific Cat. 17820843, 17850033, 17831113) to the bottom chamber.

### Proteome Profiler Array XXL (secretome analysis)

Mouse multiplex cytokine array assay kit (R&D systems Cat. ARY028) and human (R&D systems Cat. ARY022B) were used according to the manufacturer’s instructions on supernatant of cultured organoids established from isolated crypts from colon (mice, https://resources.rndsystems.com/pdfs/datasheets/ary028.pdf) or human biopsies (UC and control patients, https://resources.rndsystems.com/pdfs/datasheets/ary022b.pdf). The signal was quantitated as described previously^24^, and the medium alone signal and blanks were subtracted to obtain the normalized value.

### Immune phenotyping using flow cytometry

LP leukocytes were isolated from proximal colon, distal colon, and spleen using the STAR protocol as previously described elsewhere^66^ with Liberase TL (Roche Cat. 05401020001). Flow-cytometric staining included an unstained control and a fluorescence-minus-one (FMO) set without intracellular FOXP3 for each organ. Samples were acquired under uniform instrument settings with a Cytek Aurora and FCS Express 7. For antibodies used for flow cytometry, see Supplementary Data 8.

### Adoptive transfer

Colonic migration of injected cells was tested with CD4 + CD25+ Treg cells isolated by magnet cell separation (Miltenyi Biotech, see below). Cells were stained either with CFDA-SE (Stemcell Technologies) or Vybrant DiD (Invitrogen) according to the manufacturer. One WT mouse each received an intravenous adoptive transfer of 1 × 10^6^ WT and 1 × 10^6^ *IκBα* ^*IEC KO*^ Treg cells, differentially labeled with CMAC and DiD (switched in each mouse) using the tail vein. Another WT mouse received a NaCl injection as a control. After 3 days of 3% DSS treatment, LP leukocytes were isolated from colonic tissue as described above and analysed with Cytek Aurora.

For adoptive transfer, Wt mice received either 1 × 10^6^ T cells or CD4+CD25+ Treg cells of Wt or *IκBα* ^*IEC KO*^ donor mice ± 3% DSS treatment for 5 days (*n* = 3 per recipient group) via the tail vein. Magnetic cell separation was used to isolate T cells from splenocytes (Milteny Biotech Cat. 130-095-130) and Treg cells from pooled splenocytes and inguinal, mesenteric, and axillary lymph nodes (Milteny Biotech Cat. 130-091-041) according to the manufacturer’s protocols: https://static.miltenyibiotec.com/asset/150655405641/document_tkunq353rp5lb52e62k4pp2o0b and https://static.miltenyibiotec.com/asset/150655405641/document_vmsfagvpqp23l5kabv2nrctc4e. Purity of isolated cells was analysed with flow cytometry as described previously^67^ with Cytek Aurora, and FCS express 7.22. For antibodies/dyes used for flow cytometry, see Supplementary Data 8.

### IL-10 secretion assay without cell expansion

CD4 + CD25+ Treg cells and matched CD4 + CD25- control T cells were isolated from lymphoid organs from Wt or *IκBα* ^*IEC KO*^ mice under sterile conditions as above. 1 × 10^5^ cells were cultured in 200 µL RPMI 1640 supplemented with 10% FBS at 37 °C. 50 ng/mL PMA and 1 µg/mL ionomycin (Sigma-Aldrich) working solutions were freshly diluted and added to cultures to induce IL-10 production. Supernatants were harvested after six hours from each biological replicate, aliquoted, and stored at −80 °C for subsequent IL-10 quantification using an ELISA kit according to the manufacturer (R&D Systems Cat. M1000B) on an Infinite200Pro absorbance plate reader.

### IL-10 secretion assay after co-culture

Murine WT CD4 + CD25+ primary cells were isolated via magnetic cell separation (Miltenyi Biotech, Cat. 130-095-130) from the spleen. 2.9 × 10^4^ cells/well were seeded out in Treg cell Baseline medium, or in Treg cell Baseline medium supplemented with either Vol.−30% supernatant (SN) from mice as indicated. SN used here was collected from Wt or *IκBα*^*IEC KO*^ organoids that were cultured for 3 d in Mouse IntestiCult. CD4+CD25+ cells were cultured for 3 d and the supernatant was harvested for subsequent IL-10 ELISA quantification using an ELISA kit according to the manufacturer (R&D Systems, Cat. M1000B) on an Infinite200Pro absorbance plate reader.

### Primary Treg cell GDPD3 and CCR4 staining

Murine WT CD4^+^CD25^+^ primary cells were isolated via magnetic cell separation (Milteny Biotech, Cat. 130-095-130) from the spleen. 2.9 × 10^4^ cells/well were seeded out in Treg cell Baseline medium (RPMI supplemented with 2 mM GlutaMax, 10% FCS, 50 µM β-mercaptoethanol, 25 mM HEPES, 0.1 mM sodium pyruvate, 10 U/ml Pen/Strep, 6 ng/ml mIL-6, Dynabeads Mouse T-Activator CD3/CD28 in 2:1 bead-cell ratio), or in Treg cell Baseline medium supplemented with either 30% v/v Wt or *IκBα*^IEC KO^ supernatant (SN). SN used here was collected from Wt or *IκBα*^IEC KO^ organoids that were cultured for 3 d in Mouse IntestiCult. CD4^+^CD25^+^ cells were cultured for 3 d and afterward fixed in 4% PFA and immunofluorescently stained for GDPD3 or CCR4 (primary antibody dilution: 1:1000). Stainings were visualized using the Zeiss Axio Observer 7, and expression was quantified with ImageJ 2.1 (Fiji).

### Transwell recruitment assay (mouse)

The transwell recruitment assay used murine Wt CD4^+^CD25^+^ primary cells isolated via magnetic cell separation (Miltenyi Biotech, Cat. 130-095-130) from the spleen. 4.5 × 10^4^ cells/well were seeded in RPMI medium without additives on a transwell insert (8μm pore size, Greiner Bio-One Cat. 662638) in a 24- well plate. The bottom chamber was filled with supernatant of murine Wt or CCL22^−/−^ organoids. Organoids were treated with LPS (25 µg/ml, Sigma-Aldrich, Cat. L4269) for 24 h or left untreated. All assays were performed in triplicate. After 1.5 h incubation at 37 °C, cells were fixed with 4% PFA in PBS for 15 min, stained with 1 μg/mL DAPI for 10 min and washed with PBS. Total and migrated cells were visualized with Zeiss Axio Observer 7 and quantified with ImageJ 2.1 (Fiji).

### Suppression assay

Murine ActDsRed CD4+ T-cells, Wt and *IκBα* ^*IEC KO*^ CD4+CD25+ Treg cells were isolated using the above-mentioned Regulatory T Cell Isolation Kit (Miltenyi Biotec, 130-091-041). Wt and *IκBα* ^*IEC KO*^ Treg cells were seeded out at 7 × 10^4^ cells/well (48-well format) respectively, with ActDsRed marked CD4+ cells being added in ratios of 1:1 (Treg:CD4+ cell), 1:2, 1:4 and 1:8. Dynabeads Mouse T-Activator CD3/CD28 were added in a 1:1 ratio for CD4+ cells and in a 1:3 ratio for Treg cells (1 Treg: 3 CD3+/CD28+ T-cell activator dynabeads). Cells were cultured in Treg cell baseline medium (composition see above) for 4 d, after which the relative number of ActDsRed marked cells was assessed via FACS staining with only Zombie NIR (Biolegend, 423106) as viability marker.

### Primary CD11c and Treg cells co-culture assay

Primary CD11c^+^ cells were isolated from murine spleen using CD11c Microbeads UltraPure (Miltenyi Biotech, Cat. 130-125-835). Flow-through depleted of CD11c^+^ was subsequently used for CD4^+^CD25^+^ Treg cell isolation following the previously described magnetic cell separation protocol (Miltenyi Biotech, Cat. 130-095-130). Isolation of CD11c+ cells was confirmed by flow cytometry. CD11c+ cells were seeded and co-cultured with CD4^+^CD25^+^ cells for 3 d in a 1:5 ratio (1 × 10^4^:5 × 10^4^) in a 96-well format in technical triplicates. Previously described Treg cell Baseline medium was added, missing the Dynabeads Mouse T-Activator CD3/CD28 component that was only present in the control sample (1:2 cell to bead ratio). On day 3, co-culture supernatant was collected and used for IL-10 ELISA quantification (R&D Systems, Cat M1000B). Cell expansion was analysed via manual counting, whereas Treg cell activation marker expression was assessed via flow-cytometric staining using the Cytek Aurora system and FCS Express 7. For antibodies/dyes, see Supplementary Data 8.

### Quantification and statistical analyses

Prior to the start of the experiments, the number of animals needed or the planned patient sample numbers were calculated in consultation with the Institute of Biometry and Clinical Epidemiology using standard guidelines. Animal numbers and patient sample numbers were further approved by the animal ethics committee LaGeSo (ethics approval: G0092-18 and G-0175-23 and for human samples, by the Charité Ethics Committee EA2_046_23). Patients were not compensated for participation. Details regarding the number of samples for human biopsies or sections, or mice per group, replicates in independent experiments, and statistical tests can be found in the figure legends. The sample sizes are indicated in the figure legends. For statistical analysis, all experiments, unless stated in figure legends, were performed at least two times independently. Statistical analyses were performed using GraphPad Prism 9. Normality tests were performed to determine the proper statistical methods. Data collection and analysis were performed blind to the conditions of the experiments.

### Reporting summary

Further information on research design is available in the Nature Portfolio Reporting Summary linked to this article.

## Supplementary information

Supplementary Information

Description of Additional Supplementary Files

Supplementary Data 1

Supplementary Data 2

Supplementary Data 3

Supplementary Data 4

Supplementary Data 5

Supplementary Data 6

Supplementary Data 7

Supplementary Data 8

Reporting Summary

Transparent Peer Review file

## Source data

Source Data

## Data Availability

Single-cell sequencing data generated in this study have been deposited in SRA with the primary accession number PRJNA1282104 https://www.ncbi.nlm.nih.gov/bioproject/1282104. Processed data are available at https://zenodo.org/records/18230657. Data: https://www.ncbi.nlm.nih.gov/bioproject/1282104. Previously published dataset: GSE116222 is available here: https://www.ncbi.nlm.nih.gov/geo/query/acc.cgi?acc=GSE116222. All other data are available in the article and its Supplementary files or from the corresponding author upon request. Source data are provided with this paper.

## Code availability

The project source code can be cited via https://doi.org/10.5281/zenodo.18230657. RNA-seq analysis and reproducibility: https://github.com/BIMSBbioinfo/collab_barleben_kolesnichenko_colitis.
