# Supplementary material for: Epithelial NF-κB shapes protective Treg cell responses to restrain intestinal inflammation
Source: Nat Commun. 2026 Oct 20;17:10956. doi: 10.1038/s41467-026-78796-z (PMC13683377; doi:10.1038/s41467-026-78796-z)
Supplement: Supplementary file 2 [file 41467_2026_78796_MOESM12_ESM.pdf]

nature portfolio

Peer Review File

Epithelial NF-κB shapes protective Treg cell responses to restrain intestinal inflammation

Corresponding Author: Dr Marina Kolesnichenko

This file contains all reviewer reports in order by version, followed by all author rebuttals in order by version.

Version 0:

Reviewer comments:

Reviewer #1

(Remarks to the Author)
NF-kB is a central molecule in inflammation and is the starting point for many cascades, including the secretion of pro-inflammatory cytokines. It is a therapeutic target in several diseases, including autoimmune diseases and inflammatory bowel disease (IBD). There are many reports on the function of NF-kB in immune cells, but much remains unknown about the role of this molecule in intestinal epithelial cells (IEC).In this study, the authors used reporter mice, IEC-specific NF-kB-suppressed mice, IEC-specific NF-kB-activated mice, DSS colitis models, organoids, and human samples to show that NF-kB in the IEC prevents intestinal inflammation by repairing the epithelium and recruiting regulatory T cells (Tregs).Although they have generated compelling data using comprehensive analyses such as scRNAseq, the specific downstream signals of epithelial NF-kB that regulate epithelial repair and Treg recruitment have not been elucidated. In addition, DSS colitis, which forms the bulk of this research, is an acute chemically induced colitis model and it is difficult to say that it is a model that perfectly mimics IBD. In fact, in IBD, there is usually more than a small number of T cells infiltrating the inflamed colonic mucosa, and a decrease in Tregs has not been reported, both of which strongly contradict this study.

Major
1. In Figure 5, the authors showed that Tregs derived from IEC-specific NF-kB-activated mice had a greater suppressive effect on colitis in recipient WT mice than those derived from WT mice. Therefore, the authors suggest that the suppressive function of Tregs 'programmed' by epithelial NF-kB is enhanced, but the specific functional differences in Tregs between each donor were not investigated. IL-10 secretion ability, in vitro suppression ability and differences in gene expression should be clarified. Additionally, could the authors demonstrate that a comparable number of donor Tregs have definitively migrated to the colon in each experimental group?
2. In IEC-specific-NF-kB-suppressed mice, T-cell recruitment is impaired, while other immune cells such as macrophages are recruited. Which specific chemokines are thought to preferentially recruit T cells?
3. The organoid experiment shown in Figure 2 suggests that NF-kB in epithelial cells regulates epithelial proliferation in T cell-independent manner. Wnt has been suggested as a candidate factor for this, but this is contradicted by Extended data 2k. What factors are thought to promote epithelial proliferation downstream of NF-kB?
4. Although the recruitment of T cells is the important point of this paper, cell count is made using immunohistochemistry (IHC), which is not quantitative. As the severity of DSS colitis differs between the oral and anal sides, the number of immune cells may differ depending on which part of the tissue was taken. We recommend counting by FACS, which is more quantitative.
5. Similarly, the site of collection for IHC should be clearly stated.
6. In organoid analysis of human samples, the number of Ki67 cells and the addition of LPS should also be performed.
7. In figure 6j, the authors suggest that T cell recruitment is impaired in UC. In IEC-specific NF-kB-suppressed mice, there is less T cell infiltration during the course of DSS colitis. On the other hand, many T cells are known to infiltrate the inflamed mucosa in ulcerative colitis, and there have been no reports of a decrease in Tregs. The authors need to explain this discrepancy.
8. Also, regarding the data presented in Fig 6, the authors should at least provide a discussion on the underlying mechanisms that regulate the reduced or impaired TNF-α mediated response of UC epithelium in recruiting Tregs. Do the authors believe that the impaired response is driven by a gene-intrinsic mechanism, or is it influenced by other environmental factors ?

Minor

1. Figure 2l-n. The IHC and the graph appear to be contradictory. There seems to be a lot of Ki67 in the LPS cKO.
2. Each experiment should clearly state the number of samples and the number of experiments.

Reviewer #2

(Remarks to the Author)
I co-reviewed this manuscript with one of the reviewers who provided the listed reports. This is part of the Nature Communications initiative to facilitate training in peer review and to provide appropriate recognition for Early Career Researchers who co-review manuscripts.

Reviewer #3

(Remarks to the Author)
The manuscript uses neat scRNA-seq, biochemical, and genetic approaches to comprehensively test the function of inhibiting or constitutively activating a major TF NF-KB in IECs in colitis. The genetic approach of using the novel cell-specific NF-KB activating or repressing models with NF-kB reporter is particularly impressive. The main finding is that IEC-NFkB induces early infiltration, and perhaps reprogramming of Tregs to protect from experimental colitis. Therefore, active NF-kB in IECs is protective while repressed is worse in colitis. However, as described below there are some discrepancies, and more experimental evidence would be required to confirm this central conclusion. The manuscript also attempts to corroborate findings in human UC, but as most human IBD patients have an increased Treg and CD4 recruitment in inflamed tissue, a direct parallel is lacking.

Major Comments:

1. Abstract “While the role of immune cells in colitis is well established” and “Most secreted factors that comprise cellular communication are directly regulated by the transcription factor NF-κB” are overstatement.

2. Quantification of immune cells, like IE-T cells, PMNs and macrophages (Fig 4) should be presented as total #s. Percentages can be misleading as they can be due to a significant change in other cells, not just the numerator.

3. It is unclear how authors conclude that “epithelial suppression of NF-kB…. prevented an increase of CD4+ lymphocytes in acute colitis” when Fig 3e and 4b show a persistent increase in CD4+ T cells in acute colitis and recovery stages in N-IEC mice (that have NF-KB in IECs repressed). Is that a labeling error?

4. Authors show that Tregs are increased in IEC-IkBa KO mice. Are the Treg-inducing chemokines, and Tregs themselves reduced in the N-IEC mice?

5. Does depletion of the Treg-inducing chemokines in IkBa-IEC KO mice and supplementation in N-IEC mice restore colitis to WT level? This experiment is necessary to directly test if constitutive IEC-NFkB indeed protects from colitis by inducing Tregs.

6. The authors show that Tregs from IEC-IkBa KO are better than WT T regs in suppressing colitis. I think the manuscript will benefit from studying how constitutive NF-kB signaling reprograms Tregs to make them more suppressive.

7. Given that there is no clear “NF-kB specific” expression signature, and most human UC patients have increased Tregs in the inflamed tissue, it is unclear how the comparison between normal and UC human biopsies is relevant to the rest of the story.

Minor Points

8. Labeling NF-kB-regulated transcriptome as “pro-inflammatory” is an oversimplification, as authors also show in the course of this study.

9. What percentage of cells in IEC-IkBa KO mice are EGFP+? Is the lack of an overt phenotype in these mice due to incomplete deletion?

Reviewer #4

(Remarks to the Author)
This manuscript by Barleben et al. presents a compelling, yet potentially controversial, perspective on the role of NF-κB in colitis. While the pro-inflammatory role of NF-κB in immune cells during colitis development is well-established, the authors provide evidence for a protective function of NF-κB within intestinal epithelial cells (IECs) during the recovery phase, utilizing a combination of mouse models, single-cell RNA sequencing, and patient data. The data showing that IEC-specific NF-κB activation promotes Treg recruitment and "reprogramming" to resolve inflammation is particularly intriguing.

Although the study offers valuable insights, the presentation is hindered by several issues that compromise its clarity and rigor, just an example, The authors listed many experiments and results but did not adequately address them.
Major revision

- the authors highlight that “in contrast to the untreated mice, where activated NF-κB localised to Ki67+ stem cells, in recovery activated NF-κB was predominantly detected in CHGA+ enteroendocrine cells”; however, the function of this subset of epithelial cells was completely overlooked in the manuscript. Does Chromogranin-A involve in the recruitment of Tregs? Or others hormones?
- The study identifies different IEC subpopulations with distinct NF-κB activity. What are the specific functions of these subpopulations in colitis and recovery, and how do they coordinate their responses?
- The authors state that IEC NF-kB “reprogram” Tregs. However, the molecular mechanisms underlying this reprogramming are not fully elucidated. What specific signaling pathways or molecular interactions mediate this reprogramming of Tregs by IECs?
- The authors emphasize the critical role on epithelial cells of activated NF-κB during the recovery phase; however, they only analysed NF-κB-driven genes during the active phase of UC, where NF-κB was deregulated. What happens to these genes during remission in patients? Is there an intrinsic or extrinsic deregulation of these genes also during the mucosal healing that impact the regeneration of tissue in these patients?
- The authors claim that treatments such as corticosteroids could negatively suppress epithelial NF-κB, thereby impacting the regeneration of the epithelial compartment. As future prospective is it possible to differentiate the suppression of nFkB only on leucocytes where the authors found activation of pro-inflammatory genes? How do the authors reconcile the pro-inflammatory nature of NF-κB with its observed protective role in IECs?
- While the authors acknowledge previous discrepancies regarding NF-κB's role in IEC apoptosis, a more in-depth discussion of the factors influencing NF-κB's context-dependent functions in IECs would be beneficial.
- The authors in the figure Fig 3d report the semantic space analysis reporting in the text: GO analysis revealed that under all three conditions (homeostasis, colitis, and early recovery) genes positively regulating metabolism constituting processes including “lipid metabolism”, “sterol biosynthesis” and “cholesterol biosynthesis” that were suppressed in ΔNIEC mice; Are these pathways involved in the regeneration of the mucosa? What was their expression in IκBa IEC KO mice?
- The long list of downstream genes driven by NF-κB confirms its critical role in several cell compartments, making it quite difficult to identify a unique set responsible of its beneficial and pro-inflammatory effects. It should be relevant to better address this aspect in the manuscript.
- The authors identify a pro-inflammatory secretome, but the functional significance of individual components within this secretome remains unclear. Which specific cytokines, chemokines, or other secreted factors are most critical for Treg recruitment, activation, or reprogramming?
- Although the authors focused on epithelial-immune interactions. What is the role of mesenchymal cells in modulating IEC NF-κB signaling and its functions in colitis?

Minor revisions
Generally, The study presents interesting findings, but the manuscript could benefit from greater focus and conciseness. The extensive list of genes, while informative, appears to divert attention from the main theme of the research. A more streamlined approach would enhance the clarity and impact of the paper, ensuring that the core message is not lost amidst the details.
- The results related to Extended figure1 needs to be more integrated into the text as well as the EGFP construct more explained. The authors showed the activation of NF-κB in different tissues, but finally they focused on the colon. Is it a control to demonstrate that system works or what?
- The effects of NF-κB suppression and activation in colitis should be reported in comparison in the same graph to better appreciate its role on epithelial cells.
- How was calculated the “colitis score”? Is it based exclusively on the histology?
- In Extended figure1 d there is presented the body weight loss as % starting weight, whereas in figure 2 as weight in grams. Why this different representation? Additionally, looking at DSS impact on colits, DSS at 3% appears less strong that 1.5 % in WT, why? This variability could impact on the gene expression profiling.
- Why for quantification of isolate lymphoid follicles reported in Extended data 2b-c was used DAPI staining of colon samples and not Haematoxylin & Eosin staining or a CD45 staining in immunohistochemistry?
- Figure 1a: To improve the clearness of scheme which is confounding.
- Figure 1e: it is suggested to link with line or reported in the low magnification which one the correspondent ROI
- Figure 1j: To reduce the font size because there is a partial overlapping the row labels (gene names).
- Figure 1k: the author should consider the idea to change the type of graph for the semantic space analysis because may be confusing.
- Extended data 1d: To check the correctness of days reported in text and which reported in the graph. In the text is reported that there is a delay in the body weight loss from day 4 but in the graph is reported the day 5.
- Extended data 1g-h-i: The authors report the p value no clearly indicating which is the comparison considered. Lack a proper description in the text and in the footnote of the figure.
- Extended data 1k: To add the label of the scale bar that is missing.
- Figure 2m-n: To reduce the size font of X axis label.
- Extended data 2c-e: To reduce the size font of X axis label.
- Figure 3d: To modify the graph size to avoid the overlapping.
- Figure 4(a-d): To reduce the size font of X axis label.
- Figure 4g: To reduce the row labels to improve the readiness
- Extended data 4: The authors may better report the correspondence between cellular type and marker used to immunodetected.
- Figure 5b: The author may add the untreated DSS and healthy mice to the graph, as already reported for the colitis score (Fig. 5d) and ki67% of epithelial cells (Fig. 5f).
- Fig 6d-h: To reduce the row labels to improve the readiness

The author should be consistent in the use of American English or UK English. For example:
- the use of “leukocyte” and “leucocyte” across the paper
- the use of “visualization” and “visualisation”.
- The use of signaling and signalling
Other comments:
- Line 151: "Furthermore, we demonstrated that in IEC distinct NF-KB gene sets are activated during initiation and progression of colitis and recovery." The sentence would read better as: "Furthermore, we demonstrated that distinct NF-κB gene sets in IEC are activated during initiation, progression, and recovery of colitis."
- Line 186: "After DSS treatment, the epithelial barrier collapse was evidenced by a decrease in markers of both tight junctions and adherens junctions, Zonula occludens (ZO1) and of CDH1 respectively." The sentence is a bit convoluted
- Line 288: "Strikingly, in IkB IEC KO mice under homeostasis, we observed over six-fold increase in mucosal FoxP3+ Tregs, however no further induction occurred in colitis (Fig. 4e and f), suggesting these are likely recruited via the same NF-KB pathway." the sentence structure can be improved for clarity.
- Line 452: "Histology and immunofluorescent staining, including Multiplex IF: H&E, immunofluorescence, and multiplex stainings were performed as described previously 10, 24, 41 Colonic tissues for multiplex staining were incubated in 10% donkey serum (Sigma-Aldrich Cat. D9663 or S30-M or Abcam Cat. ab7475) and stained with DAPI (Sigma-Aldrich Cat. D9542) together with secondary antibodies (Supplemental Table 8)." This is a run-on sentence and should be broken down for clarity. Also, "immunofluorescent" should be "immunofluorescence"

Version 1:

Reviewer comments:

Reviewer #1

(Remarks to the Author)
The authors are greatly appreciated for undertaking an extensive series of additional experiments and for making a genuine effort to address all of the concerns. It is well recognized that analyzing NF-κB signaling—whose activity is highly dynamic—as well as complex intercellular interactions and the multifactorial functions of the secretome presents substantial technical and conceptual challenges.

Nevertheless, it remains difficult to conclude that all concerns have been fully and satisfactorily addressed. The remaining critical issues can be distilled into the following three major categories, excluding those that have been resolved through the additional experiments.

1. Treg reprogramming by the epithelial NFκB–dependent secretome
Major 1: The central premise of this manuscript is that chemokines contained within the epithelial NFκB–dependent secretome recruit Tregs to the epithelial niche, where they are subsequently reprogrammed. However, there is insufficient direct evidence to support the notion that downstream factors of NFκB are responsible for reprogramming Tregs. In the experiment shown in Figure 6d, does the culture supernatant from IκBαKO IEC cells increase the suppressive capacity, IL-10 production capacity and Gdpd3 expression, in addition to the expression of CCR4 and the proliferative capacity of Tregs?

Major 2: Although the authors emphasize the importance of Treg reprogramming within the epithelial niche, there appears to be a significant discrepancy between this and the observation that Tregs from the spleen and mesenteric lymph nodes (MLNs) exhibit strong suppressive capacity and elevated Gdpd3 expression. A clear, mechanistic explanation of this apparent contradiction is needed.

Major 3: Functional assays continue to rely on MLN-derived Tregs. Although the recovery of T cells from the colonic lamina propria of non-colitic mice is recognized as technically challenging, there are precedents for the use of whole $CD4^+$ T cells in this context (Makita S et al., J Immunol. 2007;178(8):4937–4946. doi:10.4049/jimmunol.178.8.4937). At a minimum, the limitations of the current experimental system should be explicitly discussed in the manuscript.

2. T-cell trafficking

Major 4: The absence of detectable differences in trafficking, as determined by FACS analysis, is a major concern. While the authors attribute this to contamination from isolated lymphoid follicles (ILFs), if the number of ILF-associated Tregs is comparable between IkBaIEC KO and WT mice, a difference should still be observable in the presence of such contamination. Are ILF Tregs increased in WT mice? If so, what evidence supports this claim?

Major 5: Although the importance of CCL22 among the chemokines in the secretome is emphasized, Figure 6A does not show significant differences between WT-col, IkBaIEC KO-ut and IkBaIEC KO-col. Furthermore, Figure 6C suggests that CCL22 is also expressed in WT-ut samples. More rigorous and quantitative analyses are therefore required.

3. Relevance of the mouse model to ulcerative colitis

Major 6: With regard to the number and function of Tregs, the claim of 'exhaustion' appears to be inconsistent with the findings in the mouse model. What is the status of Gdpd3 and CCR4 expression, which were observed in the mouse studies? Alternatively, do Tregs from ΔNIEC mice exhibit an exhaustion-like phenotype? Clarification on this point is

essential.

(Remarks on code availability)

Reviewer #2

(Remarks to the Author)
I co-reviewed this manuscript with one of the reviewers who provided the listed reports. This is part of the Nature Communications initiative to facilitate training in peer review and to provide appropriate recognition for Early Career Researchers who co-review manuscripts.

(Remarks on code availability)

Reviewer #3

(Remarks to the Author)
The manuscript is improved after revisions. It presents interesting findings but still comes across as unfocussed, with limited mechanistic insights.

Do UC-associated Tregs have reduced GDPD3? This would help draw a parallel with mouse models of perturbed epithelial NF-κB.
2. Abstract "While the role of immune cells in colitis is well established" is an overstatement that has not been edited.
3. Cellular populations such as IE-T cells, PMNs and macrophages (Fig 4) should be presented as total #s. Percentages can be misleading as they can be due to a significant change in other cells, not just the numerator. For example, Fig 4A, there seems to be no change in neutrophils between WT Ut and Col groups.
4. While of interest, the comparison between normal and UC human biopsies seems irrelevant to the rest of the story

(Remarks on code availability)

Reviewer #4

(Remarks to the Author)
The revision has improved the clarity and highlighted the strengths of the manuscript, I have no further comments.

(Remarks on code availability)

Version 2:

Reviewer comments:

Reviewer #1

(Remarks to the Author)
Although the authors have made efforts to revise the manuscript, several of the major concerns raised in the previous round of review remain inadequately addressed. As a result, the current data are still insufficient to fully support the main conclusions of the study.

1. While the manuscript emphasizes the importance of niche-specific reprogramming, it does not sufficiently account for the observed systemic changes in Treg function. The authors propose several possible mechanisms, including humoral factor–mediated effects, indirect modulation via antigen-presenting cells (APCs), and Treg recirculation. However, these remain speculative and are not experimentally validated in the present study. Such mechanisms could be addressed through approaches including parabiosis, serum transfer, ex vivo antigen presentation assays using APCs, or trafficking analyses.

2. The observed reduction of Gdpd3 expression in Tregs from UC patients is potentially an important finding and should be presented in the figures. In addition, the quality of the immunostaining requires improvement, as the co-localization of CD4 and Gdpd3 is not clearly demonstrated.

3. The presentation and interpretation of cell numbers and statistical analyses require greater rigor and clarity. It appears that the p-value on line 281 may be incorrect. A sample size of n = 3 appears insufficient. Moreover, terms such as "decreased"

should not be used in the absence of statistical significance.

(Remarks on code availability)

Reviewer #2

(Remarks to the Author)
I co-reviewed this manuscript with one of the reviewers who provided the listed reports. This is part of the Nature Communications initiative to facilitate training in peer review and to provide appropriate recognition for Early Career Researchers who co-review manuscripts.

(Remarks on code availability)

Reviewer #3

(Remarks to the Author)
Authors have provided additional data in response to previous comments. They have also better acknowledged limitations of their experiments and associated analysis.

However, as also stated by R1, the central claim that epithelial NF-kB promotes Treg recruitment and differentiation is still largely unsupported by the data:

1. There is no discernible difference in overall #s of Tregs in IEC-IkBa KO colons by FACS, and sample size in these experiments is quite small.
2. There is no in vivo support for the claim that epithelial NF-kB induced Ccl22, or any other factor, drives increased Treg infiltration or differentiation.
3. Functional analysis rely on MLN-derived Tregs

In my opinion, manuscript can be accepted if authors limit the conclusion to epithelial NFkB signaling being protective in colitis, and this protection being associated with Tregs, and leave the exact mechanisms of how epithelial NFkB protect from colitis as yet-undetermined.

(Remarks on code availability)

Version 3:

Reviewer comments:

Reviewer #1

(Remarks to the Author)
The new Extended data provide meaningful functional support for the authors' central model. In particular, conditioned medium from NF-κB-activated epithelial organoids enhanced IL-10 production by primary Tregs independently of CD11c+ cells, supporting a direct effect of one or more epithelial secreted factors on Treg function.

However, the molecular basis of this effect remains unresolved. Although the secretome analysis identified plausible candidates, including IL-33, the responsible factor or combination of factors was not experimentally defined. Thus, the revised study establishes a functional epithelial secretome–Treg link in vitro, but does not yet provide a molecular mechanism for this interaction or demonstrate its causal contribution in vivo.

The human GDPD3 analysis was less informative. GDPD3+ Tregs were too rare in the UC datasets to permit a robust conclusion, and the enrichment observed in colorectal cancer does not directly establish their relevance to UC. This reduces the translational impact of this aspect of the study, although it does not invalidate the principal findings.

(Remarks on code availability)

Reviewer #2

(Remarks to the Author)
I co-reviewed this manuscript with one of the reviewers who provided the listed reports. This is part of the Nature Communications initiative to facilitate training in peer review and to provide appropriate recognition for Early Career

Researchers who co-review manuscripts.

(Remarks on code availability)

Reviewer #3

(Remarks to the Author)
Authors have provided new data or modified their conclusions to sufficiently assuage my concerns.

(Remarks on code availability)

Open Access This Peer Review File is licensed under a Creative Commons Attribution 4.0 International License, which permits use, sharing, adaptation, distribution and reproduction in any medium or format, as long as you give appropriate credit to the original author(s) and the source, provide a link to the Creative Commons license, and indicate if changes were made.
In cases where reviewers are anonymous, credit should be given to 'Anonymous Referee' and the source.
The images or other third party material in this Peer Review File are included in the article's Creative Commons license, unless indicated otherwise in a credit line to the material. If material is not included in the article's Creative Commons license and your intended use is not permitted by statutory regulation or exceeds the permitted use, you will need to obtain permission directly from the copyright holder.
To view a copy of this license, visit https://creativecommons.org/licenses/by/4.0/

Reviewer's Comments:

Reviewer #1 (Remarks to the Author)

NF-kB is a central molecule in inflammation and is the starting point for many cascades, including the secretion of pro-inflammatory cytokines. It is a therapeutic target in several diseases, including autoimmune diseases and inflammatory bowel disease (IBD). There are many reports on the function of NF-kB in immune cells, but much remains unknown about the role of this molecule in intestinal epithelial cells (IEC).In this study, the authors used reporter mice, IEC-specific NF-kB-suppressed mice, IEC-specific NF-kB-activated mice, DSS colitis models, organoids, and human samples to show that NF-kB in the IEC prevents intestinal inflammation by repairing the epithelium and recruiting regulatory T cells (Tregs).Although they have generated compelling data using comprehensive analyses such as scRNAseq, the specific downstream signals of epithelial NF-kB that regulate epithelial repair and Treg recruitment have not been elucidated. In addition, DSS colitis, which forms the bulk of this research, is an acute chemically induced colitis model and it is difficult to say that it is a model that perfectly mimics IBD. In fact, in IBD, there is usually more than a small number of T cells infiltrating the inflamed colonic mucosa, and a decrease in Tregs has not been reported, both of which strongly contradict this study.

We thank the reviewer for this insightful summary and agree that defining the downstream mediators of epithelial NF-κB is essential. In response, we performed additional experiments, including scRNA-seq of colonic and splenic T cells, *in vitro* migration assays, and validation in complementary mouse models, which allowed us to identify discrete chemokines and epithelial-derived factors that govern both Treg recruitment and their functional reprogramming. While DSS colitis is indeed an acute injury model, it robustly captures epithelial barrier failure, a central pathogenic feature shared with human IBD, and is widely used to dissect epithelial-immune crosstalk *in vivo*. To address disease relevance directly, we incorporated new analyses of human UC biopsies and found that epithelial NF-κB activity in UC is not appropriately activated but instead dysregulated, which likely impairs directed recruitment and functional programming of leukocytes, including Tregs. These additional data help reconcile differences between mouse and human settings and clarify the epithelial NF-κB–dependent signals that shape Treg behavior. Together, they strengthen the mechanistic and translational significance of our conclusions. Below, we address each comment in a detailed point-by-point response.

Major

1. In Figure 5, the authors showed that Tregs derived from IEC-specific NF-kB-activated mice had a greater suppressive effect on colitis in recipient WT mice than those derived from WT mice. Therefore, the authors suggest that the suppressive function of Tregs 'programmed' by epithelial NF-kB is enhanced, but the specific functional differences in Tregs between each donor were not investigated. IL-10 secretion ability, in vitro suppression ability and differences in gene expression should be clarified. Additionally, could the authors demonstrate that a comparable number of donor Tregs have definitively migrated to the colon in each experimental group?

We thank the reviewer for recommending specific experiments which addressed an important point raised by the findings. We performed experiments to address IL-10 secretion, suppression assay and in addition scRNA-seq of immune cells from colons and spleens of $I\kappa B\alpha^{IEC\ KO}$ mice and Wt littermates to determine if the phenotype is local or systemic. Below are our findings.

IL-10 secretion: We have isolated Tregs from colons of *IκBα* $^{IEC\ KO}$ mice and Wt littermates. The supernatant from the medium was analysed for IL-10 by ELISA. Tregs from *IκBα* $^{IEC\ KO}$ mice produced over 100-fold more IL-10 that Tregs from the Wt littermates. Since IL-10 is required for suppression of Th-17 mediated inflammation (*7*), increased secretion of the cytokine indicates better suppressive capabilities (New Figure 6f).

Suppression assay: Functionally accessing colonic Tregs in a suppression assay is inherently challenging because only very small numbers can be isolated directly from the colon. Obtaining sufficient cell numbers therefore requires in vitro expansion; however, prolonged stimulation with αCD3/αCD28 and high-dose IL-2 is well known to override tissue-imprinted programs and to normalize the phenotype of gut-derived Tregs, thereby obscuring epithelial-driven reprogramming effects. To minimize manipulation, we isolated splenic Tregs (which according to the scRNA profile maintained at least some reprogramming phenotype) from *IκBα* $^{IEC\ KO}$ and Wt littermates and initiated suppression assays immediately using primary ActdsRED $CD4^{+}$ responder cells. Although we observed normal dose-dependent suppression, the *IκBα* $^{IEC\ KO}$ –specific phenotype was no longer detectable after four days of co-culture, consistent with phenotype erosion *in vitro* (New Extended data 6d). Because suppression assays require extended culture outside the epithelial niche that drives Treg reprogramming, this assay is not suitable for reliably capturing the phenotype in this context.

Activation marker expression: CCR4 is expressed mainly on effector Treg cells (*8*) and therefore marks a functionally more suppressive subset. We therefore analysed expression of CCR4 on freshly isolated Tregs from *IκBα* $^{IEC\ KO}$ mice and Wt littermates by flow cytometry and confirmed our findings by immunofluorescence staining using CD4 and CCR4 antibodies. FACS analysis revealed significantly increased expression of CCR4 on isolated Tregs from *IκBα* $^{IEC\ KO}$ mice (New Figure 6f and Supplemental Figure 2). This indicates that *IκBα* $^{IEC\ KO}$ mice contain more effector Tregs.

Differential gene expression: To determine how the Tregs differ between the *IκBα* $^{IEC\ KO}$ mice and Wt mice we performed scRNA-seq on isolated immune cells from whole colon and from the spleen (New Figure 6g). Remarkably, epithelial NF-κB activation reprogrammed Tregs not only locally in the colon but also systemically (New Figure 6h and Extended data 6e). Among differentially expressed genes, *Gdpd3* (Glycerophosphodiester phosphodiesterase domain–containing 3) was almost exclusively detected in Tregs from *IκBα* $^{IEC\ KO}$ mice (New Fig. 6h and i). Although *Gdpd3* has been mentioned rarely in the literature, previous work suggests its association with memory T-cell features (*9*) and is required for leukaemia stem cell survival (*10*). Based on this, we hypothesized that epithelial NF-κB confers enhanced activation and memory-like properties to Tregs, thereby supporting their regenerative functions. We confirmed the presence of Gdpd3 also on protein level by immunofluorescence (New Figure 6j and k). Here we detected a significant increase in Gdpd3+ CD4+ cells in *IκBα* $^{IEC\ KO}$ mice, confirming metabolic rewiring in Tregs driven through NF-κB.

In summary, epithelial NF-κB activation generates a more potent effector Treg population, marked by increased CCR4 expression and over 100-fold higher IL-10 secretion, indicating substantially enhanced suppressive function. scRNA-seq further revealed systemic Treg reprogramming with selective upregulation of Gdpd3, a memory-associated gene, supporting a model in which epithelial NF-κB enhances Treg activation, stability, and regenerative capacity. Further work is needed to determine how GDPD3 shapes the Treg phenotype, however this is beyond the scope of the current project.

2. In IEC-specific-NF-kB-suppressed mice, T-cell recruitment is impaired, while other immune cells such as macrophages are recruited. Which specific chemokines are thought to preferentially recruit T cells?

We thank the reviewer for this very important question. Addressing this question has led us to the identification of the chemokine we believe is one of the key downstream mediators of the protective NF-κB-mediated phenotype we observed.
We have previously shown that whereas in ΔN^{IEC} mice T cell recruitment is impaired, in the $I\kappa B\alpha^{IEC\ KO}$ mice more CD4+, and specifically CD4+ FoxP3+ cells are present in mucosa already in homeostasis (Figure 4f and g).
In the original manuscript we have identified secreted and epithelium-specific factors regulated by NF-κB by a protein array on the medium produced by non-passaged epithelial organoids. These were derived from crypts of Wt mice or $I\kappa B\alpha^{IEC\ KO}$ littermates that were treated with DSS or untreated controls. ΔN^{IEC} crypts failed to proliferate past day 1 and therefore could not be used for this experiment (Extended data 2k). Of the secreted factors, chemokines Ccl22, Ccl5, and Cxcl10 are known as direct targets of NF-κB (bold in the heatmap) and showed a significant increase (Figure 6a). Although all these could potentially recruit macrophages, they, and especially Ccl22, are recognized as central in the context of T cell recruitment (*11-13*).

Of these, Ccl22 showed the strongest induction in an NF-κB and in a colitis-dependent manner (Figure 6a). The only known receptor (so far) for Ccl22 is the C-C Chemokine Receptor 4 (CCR4), which is present on regulatory T cells (Tregs) under homeostasis(*12*) and can be induced on other cells types under stress. Ccl22 is one of the key chemokines for Treg trafficking(*12, 14*). It was recently shown to mediate protective functions via Treg-dendritic cell interactions in DSS-induced colitis by the landmark paper from the David Anz group(*12*). Ccl22 however is also secreted by intestinal epithelial cells (*15*). We observed Ccl22 in epithelial cells, predominantly in DSS treated mice and also in $I\kappa B\alpha^{IEC\ KO}$ littermates already in homeostasis (New Figure 6c). Together, these findings identify the intestinal epithelium as a previously underappreciated, physiologically relevant source of CCL22 in the colon.
To determine if the chemokines we identified in the protein array, including Ccl22, can directly recruit Tregs, we performed a transwell assay with isolated primary Tregs from Wt mice (New Figure 6b). Tregs were stimulated with Ccl22 or Ccl5 (chemokine showing highest induction in DSS Wt and in $I\kappa B\alpha^{IEC\ KO}$ untreated mice) and Cxcl10 (control for recruitment) . In comparison to the negative control (medium only) all three potently led to Treg migration.
Ccl22 knockout mice *Ccl22tm1(KOMP)Vlcg* are viable without an overt phenotype under basal conditions. It was previously shown by David Anz and colleagues that Ccl22-/- mice have excessive T cell immunity and are hypersensitive to DSS-induced colitis (*12*). These mice fail to recruit Tregs.
Although generating Ccl22 IEC KO mice is beyond the scope of the current work, we stained epithelium under conditions as before (colitis and recovery, and also from $I\kappa B\alpha^{IEC\ KO}$ colons) with Ccl22 antibody. IHC confirmed presence of Ccl22 under homeostasis in $I\kappa B\alpha^{IEC\ KO}$ colons, and only after DSS treatment in Wt mice (New Figure 6c). Obtaining an ethics permit in Berlin to generate a double knockout mice with tissue specific suppression of Ccl22 and *Nfkbia* for DSS treatment would require several months. We hypothesize that if Ccl22 is the key mediator for Treg recruitment, downstream of NF-κB in epithelium, such mice would lose protection observed in $I\kappa B\alpha^{IEC\ KO}$ animals.

3. The organoid experiment shown in Figure 2 suggests that NF-kB in epithelial cells regulates epithelial proliferation in T cell-independent manner. Wnt has been suggested as a candidate factor for this, but this is contradicted by Extended data 2k. What factors are thought to promote epithelial proliferation downstream of NF-kB?

This is an important point and despite our efforts to identify such factors, which would be downregulated in *ΔN*^{IEC} mice but are up *IκBα* $^{IEC\ KO}$ animals, we were only able to find those that regulate leucocyte recruitment - such as *Cxcl1*, *Cxcl2* and *Lcn2.*

We did not exclude Wnt signalling and other Wnt ligands derived from epithelial cells themselves as factors responsible for lack of growth of *ΔN*^{IEC} organoids. Our RNAseq analysis of crypts from the *ΔN*^{IEC} mice shows that indeed *Wnt1* and *Wnt7b* are top downregulated genes (Supplemental Table 3). No other Wnt ligands showed changes in expression. *Wnt1* expression dropped to almost non-detectable (log2fold = -44.5 ($p < 0.0001$). Similarly, *Wnt7b* expression dropped to near 0 (log2fold = -16.3 ($p< 0.0001$). The role of Wnt7b in the gut is not well established, although reports using intestinal organoids show that it promotes regeneration upon injury *in vitro(16)*. Wnt1 is more associated with signalling in the stomach. Although changes in Wnt can account for lack of growth of organoids from *ΔN*^{IEC} mice, since we did not see the reverse phenotype (increase in expression of *Wnt1* and *Wnt7b* in *IκBα* $^{IEC\ KO}$ epithelium), we did not pursue this as the protective mechanism responsible for increased resistance against colitis in *IκBα* $^{IEC\ KO}$ mice.

Finally, because untreated *ΔN*^{IEC} mice do not exhibit impaired growth *in vivo*, we cannot exclude the possibility that the reduced growth of *ΔN*^{IEC} - derived organoids reflects an *in vitro* artifact specific to the organoid system. For these reasons, we focused our efforts on epithelial factors with clear, mirrored regulation between the two models, namely chemokines involved in leukocyte recruitment.

4. Although the recruitment of T cells is the important point of this paper, cell count is made using immunohistochemistry (IHC), which is not quantitative. As the severity of DSS colitis differs between the oral and anal sides, the number of immune cells may differ depending on which part of the tissue was taken. We recommend counting by FACS, which is more quantitative.

We followed the recommendation and quantitated immune cells both in the colon (location indicated in legends) and in the spleen. Our new data, obtained by immune phenotyping through flow cytometry, confirmed the initial observation that CD11b+ Ly6G+ cells decrease in distal colon, with a concomitant increase in CD4+ FoxP3+ cells in *IκBα* $^{IEC\ KO}$ mice. In addition, we also analysed proximal colon – although we detected a twofold increase in Tregs, this difference was not significant (New main figure 4a-c, Extended data 4a-d, and gating strategy in Supplemental Figure 1). The differences however were not as pronounced as detected by immunofluorescence, likely because flow cytometry of colonic immune cells does not differentiate between cells derived from ILF versus intraepithelial cells.

5. Similarly, the site of collection for IHC should be clearly stated.

We have now included this information in figure legends for new figures and updated the figures in the original manuscript.

6. In organoid analysis of human samples, the number of Ki67 cells and the addition of LPS should also be performed.

We thank the reviewer for this excellent suggestion for an experiment. We have now performed these experiments using organoids from non-IBD “healthy” crypts and organoids established from crypts from UC patients. We detected a no increase in Ki67 cells but an increase in caspase 3 cleaved + cells UC organoids treated with LPS. This is consistent with our observation that UC IEC contains intrinsic defects which can be propagated even when IEC is removed from the inflammatory milieu (New Figure 7f-h). A likely explanation is that chronic barrier dysfunction in UC leaves epithelial cells in a persistently stress-sensitized

state, making them more prone to apoptosis and less capable of mounting a compensatory proliferative response upon LPS stimulation.

7. In figure 6j, the authors suggest that T cell recruitment is impaired in UC. In IEC-specific NF-kB-suppressed mice, there is less T cell infiltration during the course of DSS colitis. On the other hand, many T cells are known to infiltrate the inflamed mucosa in ulcerative colitis, and there have been no reports of a decrease in Tregs. The authors need to explain this discrepancy.

This is a very important point. Although several reports describe increased Treg numbers in UC, more recent studies have also documented a reduction in mucosal Tregs (*3, 17*). Our findings help reconcile these discrepancies by demonstrating that it is not the absolute number of Tregs, but rather their appropriate programming and functional state, that determines effective resolution of colitis. Consistent with prior work showing impaired effector-Treg function and features of exhaustion in UC (*18, 19*), our analysis of $CD4^{+}CD25^{+}$ Tregs from healthy controls and from inflamed UC tissue revealed clear phenotypic differences, with UC-derived Tregs exhibiting markers of T-cell exhaustion (Figure for Reviewers only A).

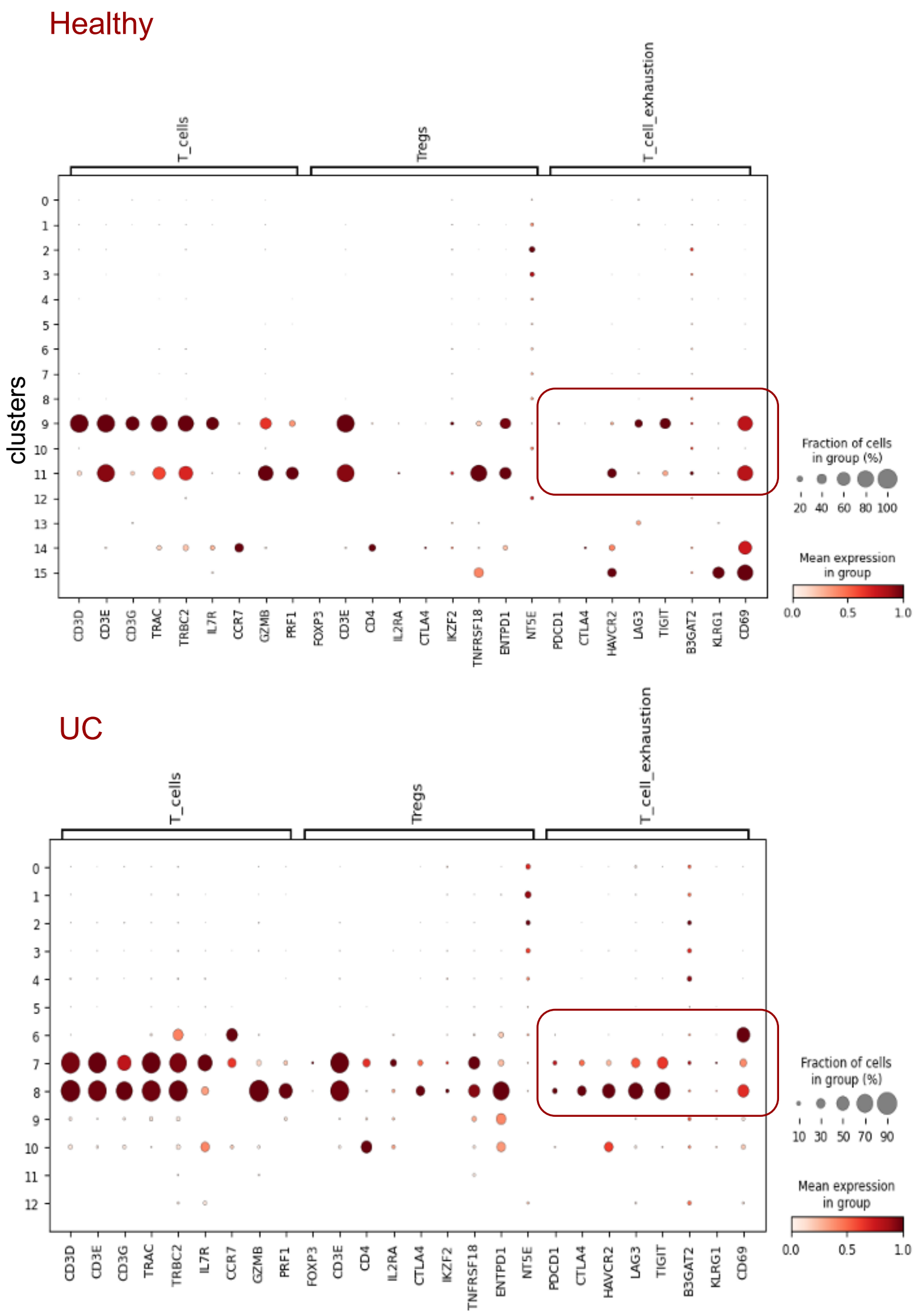

Figure for Reviewers only A: Differential analysis of scRNA dataset (*GSE116222)* showing changes in expression of genes associated with T cell exhaustion in healthy versus UC patient colons.

Our Figure 7g specifically measures IEC-driven chemotaxis and highlights that epithelial NF-κB normally helps maintain a tolerogenic niche by producing Treg-attracting chemokines (e.g., CCL22, CCL28, CCL20), which engage CCR4/CCR10/CCR6 on effector Tregs. When epithelial NF-κB is suppressed in our mouse model, or dysregulated in UC epithelium, these niche-forming cues diminish, reducing the directed recruitment and retention of Tregs at the epithelial interface, even though global inflammation continues to drive high overall $CD3^+$ infiltration. This distinction aligns with the emerging view that immune tolerance is sustained within specialized micro-niches in the lamina propria rather than uniformly across the inflamed mucosa. As shown by Powrie and colleagues (*20*), effector Tregs require spatially organized interactions with $CD206^+$ macrophages and $CD103^+SIRP\alpha^+$ dendritic cells to maintain suppressive function; inflammation disrupts these neighbourhoods and compromises compartmentalized tolerance. Our findings therefore support the conclusion that loss of IEC-derived, NF-κB–dependent signals destabilize these Treg-supportive microniches. By defining the epithelial contribution to Treg positioning and programming, our results complement clinical observations that adoptive transfer of autologous Tregs can benefit refractory UC (*3*). We have now incorporated this clarification into the manuscript and the discussion.

8. Also, regarding the data presented in Fig 6, the authors should at least provide a discussion on the underlying mechanisms that regulate the reduced or impaired TNF-α mediated response of UC epithelium in recruiting Tregs. Do the authors believe that the impaired response is driven by a gene-intrinsic mechanism, or is it influenced by other environmental factors ?

Our data show that UC-derived intestinal organoids fail to mount a TNF-α–induced secretome capable of efficiently recruiting T cells, suggesting that epithelial NF-κB–dependent responses are compromised. The mechanisms underlying this defect are likely multifactorial. However, our organoid experiments point to a substantial cell-intrinsic epithelial component: even in a standardized culture environment devoid of immune cells and inflammatory cues, UC epithelium exhibits blunted induction of NF-κB target genes (New Figure 7 h and i) and sensitization to LPS (New Figure 7e and f).
We hypothesize that stable epithelial alterations, such as epigenetic modifications previously reported in UC (*21*), impair the amplitude, timing, or target specificity of NF-κB signalling, thereby limiting the epithelium's capacity to initiate appropriate Treg-recruiting programmes. At the same time, we do not exclude a contribution of extrinsic factors in vivo, including chronic cytokine exposure, microbiota-derived signals, or stromal–immune interactions, which may further reinforce this deregulated epithelial state.
Within this framework, the paradoxical increase in Treg numbers observed in some UC patients can be reconciled: in the absence of proper IEC-mediated instruction, Tregs may accumulate but remain functionally insufficient to suppress inflammation or promote epithelial repair. We have incorporated this point into the Discussion.

Minor
1. Figure 2l-n. The IHC and the graph appear to be contradictory. There seems to be a lot of Ki67 in the LPS cKO.

We have replaced the image with the more representative one.

2. Each experiment should clearly state the number of samples and the number of experiments.

We have updated this information.

Reviewer #2 (Remarks to the Author)

I co-reviewed this manuscript with one of the reviewers who provided the listed reports. This is part of the Nature Communications initiative to facilitate training in peer review and to provide appropriate recognition for Early Career Researchers who co-review manuscripts.

We thank the reviewer for participating in the peer review and contributing to improvement of our manuscript.

Reviewer #3 (Remarks to the Author)

The manuscript uses neat scRNA-seq, biochemical, and genetic approaches to comprehensively test the function of inhibiting or constitutively activating a major TF NF-KB in IECs in colitis. The genetic approach of using the novel cell-specific NF-KB activating or repressing models with NF-kB reporter is particularly impressive. The main finding is that IEC-NFkB induces early infiltration, and perhaps reprogramming of Tregs to protect from experimental colitis. Therefore, active NF-kB in IECs is protective while repressed is worse in colitis. However, as described below there are some discrepancies, and more experimental evidence would be required to confirm this central conclusion. The manuscript also attempts to corroborate findings in human UC, but as most human IBD patients have an increased Treg and CD4 recruitment in inflamed tissue, a direct parallel is lacking.

We thank the reviewer for the positive assessment of our genetic models and multi-modal experimental approach. We fully agree that defining the mechanistic basis of IEC-driven Treg recruitment and reprogramming is central to the study, and in response to the reviewer's concerns we have performed additional experiments, including scRNA-seq of colonic and splenic T cells, *in vitro* migration assays, and validation in complementary mouse model, which strengthen and confirm this conclusion. Regarding the human UC data, we appreciate the reviewer's point and now clarify in the revised manuscript that although some reports show that inflamed UC mucosa contains increased total $CD4^+$ and Treg infiltration, these Tregs frequently exhibit exhaustion and impaired effector-Treg function. Our new analyses of UC biopsies and organoids further support this distinction by showing dysregulated epithelial NF-κB activity. Together, these additions reconcile the apparent discrepancy between human UC and our mouse models and reinforce the translational relevance of our findings.

Major Comments:

1. Abstract "While the role of immune cells in colitis is well established" and "Most secreted factors that comprise cellular communication are directly regulated by the transcription factor NF-κB" are overstatement.

We have changed this sentence in the abstract.

2. Quantification of immune cells, like IE-T cells, PMNs and macrophages (Fig 4) should be presented as total #s. Percentages can be misleading as they can be due to a significant change in other cells, not just the numerator.

Thank you for this useful suggestion. Another reviewer likewise asked for quantification by flow cytometry, and also specification of the site analysed. We now analysed Cd11b+ cells, including F4/80+ and Ly6G+ cells from proximal and distal colon and spleen, and also T cells, including FoxP3+ and CD25+ Tregs from the same locations. (New Figure 4a-c and Extended Data 4a-c, and supplemental Figure 1).

We show that $CD4^+FoxP3^+$ Tregs accumulate in the distal colon of $I\kappa B\alpha^{IEC\ KO}$ mice. In the proximal colon we observed a similar trend, with approximately twofold increase in Tregs, although this difference did not reach statistical significance. The differences however were not as pronounced as detected by immunofluorescence, likely because flow cytometry of colonic immune cells does not differentiate between cells derived from ILF versus intraepithelial cells.

3. It is unclear how authors conclude that "epithelial suppression of NF-kB…. prevented an increase of CD4+ lymphocytes in acute colitis" when Fig 3e and 4b show a persistent increase in CD4+ T cells in acute colitis and recovery stages in N-IEC mice (that have NF-KB in IECs repressed). Is that a labeling error?

We have now rephrased this in the manuscript and quantitated T cell recruitment, as recommended by flow cytometry to provide more accurate immune cell numbers (New Figures 4a-c and Extended data 4a-c and Supplemental Figure 1).

4. Authors show that Tregs are increased in IEC-IkBa KO mice. Are the Treg-inducing chemokines, and Tregs themselves reduced in the N-IEC mice?

The following factors are known to induce Treg recruitment: Ccl22, Ccl5, Ccl20, Ccl28, and Cxcl12. We analysed their expression using RNA-seq on crypts from ΔN^{IEC} mice or Wt littermates treated with DSS. All mentioned factors showed a reduction in ΔN^{IEC} mice, however since we relied on bulk sequencing and four mice per group, not all changes reached significance (Supplemental Table 3).

Ccl5 shows over two-fold decrease in ΔN^{IEC} mice after DSS treatment (p value = 0.007, adjusted p value = 0.09) and *Ccl22* shows a reduction, however the adjusted p value did not reach significance. *Ccl20* shows over a two-fold reduction (adjusted p value = 0.1). In summary, these coordinated downward trends across multiple Treg-attracting chemokines support the conclusion that suppression of epithelial NF-κB diminishes the chemotactic cues required for effective Treg recruitment, consistent with the reduced Treg presence observed in ΔN^{IEC} mice.

5. Does depletion of the Treg-inducing chemokines in IkBa-IEC KO mice and supplementation in N-IEC mice restore colitis to WT level? This experiment is necessary to directly test if constitutive IEC-NFkB indeed protects from colitis by inducing Tregs.

Analysis of the transcriptome and secretome identified chemokines that recruit T cells, specifically Tregs, as induced by epithelial NF-κB. Some of these showed robust reduction in expression in the ΔN^{IEC} mice that are highly susceptible to colitis.
Ccl22 showed the strongest induction both in Wt mice treated with DSS (Figure 6a), and also in the $I\kappa B\alpha^{IEC\ KO}$ mice already in homeostasis. We performed *in vitro* recruitment assay to show that Ccl22, Ccl5 and Cxcl10 can robustly recruit Tregs (Figure 6b).

The reviewer recommended supplementation of mice with chemokines ΔN^{IEC} mice as a rescue *in vivo* experiment. Since we detected several and not a single factor regulated by NF-κB to achieve resolution, it might not be possible to identify the dosage to recapitulate the *in vivo* setting. However, experiments showing that some of the key factor we identified as induced in an epithelial NF-κB-dependent manner were already performed*:* we provide a summary below, followed by experiments we performed to validate our findings.

Ccl22 -/- mice do not have an overt phenotype, however, are sensitized to DSS-induced colitis due to defective Treg recruitment (*12*). Supplementation of mice with Ccl22

ameliorates DSS-induced colitis (*22*). Previous report has demonstrated that Ccl22 can be produced by intestinal epithelial cells (*15*).
Creating transgenic Ccl22 IEC KO *IκBα* $^{IEC\ KO}$ mice would require embryo transfer and approval from the local ethics authorities – therefore due to time constraints were not pursued. We confirmed however that Ccl22 can be produced by intestinal epithelium and indeed *IκBα* $^{IEC\ KO}$ mice produce more already under homeostasis (New Figure 6c).
We hypothesize that if Ccl22 is a key mediator of Treg recruitment to epithelium, then the Ccl22 IEC KO *IκBα* $^{IEC\ KO}$ mice would lose the protection from constitutively activated NF-κB. Our secretome analysis however uncovered numerous mediator both of recruitment and reprogramming, therefore a more likely scenario is a reduce recruitment potential with epithelium-specific knockout of each individual factor (e.g., Ccl22 or Ccl5).
We therefore hope that the reviewer finds the new in vitro assays shown in the new Fig 6 and the scRNA analysis sufficient as a response.

6. The authors show that Tregs from IEC-IkBa KO are better than WT T regs in suppressing colitis. I think the manuscript will benefit from studying how constitutive NF-kB signaling reprograms Tregs to make them more suppressive.

We approached this by analysing Treg phenotypes, functions, and transcriptomes in Wt versus in *IκBα* $^{IEC\ KO}$ mice. Since reviewer 1 asked similar question (question 1) we summarize the findings below.

Phenotype: CCR4 is expressed mainly on effector Treg cells (*8*) and therefore marks a functionally more suppressive subset. We therefore analysed expression of CCR4 on freshly isolated Tregs from *IκBα* $^{IEC\ KO}$ mice and Wt littermates by flow cytometry and confirmed our findings by immunofluorescence staining using CD4 and CCR4 antibodies. FACS analysis revealed significantly increased expression of CCR4 on isolated Tregs from *IκBα* $^{IEC\ KO}$ mice (New Figure 6f). This indicates that *IκBα* $^{IEC\ KO}$ mice contain more effector Tregs.

Functions: We have isolated Tregs from colons of *IκBα* $^{IEC\ KO}$ mice and Wt littermates. The supernatant from the medium was analysed for IL-10 by ELISA. Tregs from *IκBα* $^{IEC\ KO}$ mice produced over 100-fold more IL-10 that Tregs from the Wt littermates. Since IL-10 is required for suppression of Th-17 mediated inflammation (*7*), increased secretion of the cytokine indicates better suppressive capabilities (New Figure 6f).

Suppression assay: Functionally assessing colonic Tregs in a suppression assay is inherently challenging because only very small numbers can be isolated directly from the colon. Obtaining sufficient cell numbers therefore requires in vitro expansion; however, prolonged stimulation with αCD3/αCD28 and high-dose IL-2 is well known to override tissue-imprinted programs and to normalize the phenotype of gut-derived Tregs, thereby obscuring epithelial-driven reprogramming effects. To minimize manipulation, we isolated splenic Tregs from *IκBα* $^{IEC\ KO}$ and Wt littermates and initiated suppression assays immediately using primary ActdsRED $CD4^+$ responder cells. Although we observed normal dose-dependent suppression, the *IκBα* $^{IEC\ KO}$ –specific phenotype was no longer detectable after four days of co-culture, consistent with phenotype erosion *in vitro* (Extended data 6d). Because suppression assays require extended culture outside the epithelial niche that drives Treg reprogramming, this assay is not suitable for reliably capturing the phenotype in this context.

Characterization of Treg reprogramming: Since we have established that Tregs derived from colon of *IκBα* $^{IEC\ KO}$ mice have an enhanced suppressive phenotype, we performed scRNA-seq on colonic versus splenic leucocytes to determine whether how Tregs are reprogrammed and also whether the reprogramming remains local or is systemic (New

Figure 6g). Remarkably, epithelial NF-κB activation reprogrammed Tregs not only locally in the colon but also systemically. Among differentially expressed genes, *Gdpd3* (Glycerophosphodiester phosphodiesterase domain–containing 3) was almost exclusively detected in Tregs from *IκBα* $^{IEC\ KO}$ mice (New Fig. 6h and Extended data 6e). Although *Gdpd3* has been mentioned only rarely in the literature, previous work suggests it is associated with memory T-cell features (*9*) and is required for leukaemia stem cell survival (*10*). Based on this, we hypothesized that epithelial NF-κB confers enhanced activation and memory-like properties to Tregs, thereby supporting their regenerative functions. We confirmed presence of Gdpd3 also on protein level by immunofluorescence (New Figure 6j and k). Here we detected a significant increase in Gdpd3+ CD4+ cells in *IκBα* $^{IEC\ KO}$ mice, confirming metabolic rewiring in Tregs driven through NF-κB.

In summary, we show clear reprogramming of Tregs via epithelial NF-κB using the three methods: Tregs from $I\kappa B\alpha^{IEC\ KO}$ mice show increased activation, evidenced by increased CCR4 levels and increased IL-10 secretion. We furthermore demonstrated that they possess a distinct transcriptional signature with increased expression and production of Gdpd3+, previously proposed a marker of memory T cells.

7. Given that there is no clear "NF-kB specific" expression signature, and most human UC patients have increased Tregs in the inflamed tissue, it is unclear how the comparison between normal and UC human biopsies is relevant to the rest of the story.

Our data in mice shows that activated epithelial NF-κB is protective. We show that in UC patients epithelial NF-κB is not activated, but is rather deregulated, therefore the protective mechanisms responsible for recruitment of immune cells to resolve colitis are absent. Although several reports describe increased Treg numbers in UC, more recent studies have also documented a reduction in mucosal Tregs(*3, 17*). Our findings help reconcile these discrepancies by demonstrating that it is not the absolute number of Tregs, but rather their appropriate programming and functional state, that determines effective resolution of colitis. Consistent with prior work showing impaired effector-Treg function and features of exhaustion in UC (*18, 19*), our analysis of $CD4^+CD25^+$ Tregs from healthy controls and from inflamed UC tissue revealed clear phenotypic differences, with UC-derived Tregs exhibiting markers of T-cell exhaustion (Data for reviewer only A).

In Figure 7 we measured IEC-driven chemotaxis and showed that epithelial NF-κB normally helps maintain a tolerogenic niche by producing Treg-attracting chemokines (e.g., CCL22, CCL28, CCL20), which engage CCR4/CCR10/CCR6 on effector Tregs. When epithelial NF-κB is suppressed in our mouse model, or dysregulated in UC epithelium, these niche-forming cues diminish, reducing the directed recruitment and retention of Tregs at the epithelial interface, even though global inflammation continues to drive high overall $CD3^+$ infiltration. This distinction aligns with the emerging view that immune tolerance is sustained within specialized micro-niches in the lamina propria rather than uniformly across the inflamed mucosa. As shown by Powrie and colleagues (*20*), effector Tregs require spatially organized interactions with $CD206^+$ macrophages and $CD103^+SIRP\alpha^+$ dendritic cells to maintain suppressive function; inflammation disrupts these neighbourhoods and compromises compartmentalized tolerance. Our findings therefore support the conclusion that loss of IEC-derived, NF-κB–dependent signals destabilize these Treg-supportive microniches. By defining the epithelial contribution to Treg positioning and programming, our results complement clinical observations that adoptive transfer of autologous Tregs can benefit refractory UC (*3*). We have now incorporated this clarification into the manuscript.

Minor Points

8. Labelling NF-kB-regulated transcriptome as "pro-inflammatory" is an oversimplification, as authors also show in the course of this study.

We agree with this statement, however we are not aware of a term which would better represent what the secretome does – promote recruitment of immune cells, which would normally result in inflammation. However, we now include this important point in the discussion in the last paragraph.

9. What percentage of cells in IEC-IkBa KO mice are EGFP+? Is the lack of an overt phenotype in these mice due to incomplete deletion?

In comparison to wild-type mice, in which fewer than 5% of IECs are $EGFP^+$ under homeostatic conditions, approximately 15% of IECs are $EGFP^+$ in $I\kappa B\alpha^{IEC\ KO}$ mice (new Extended Data Fig. 2d). Importantly, EGFP positivity reflects NF-κB activation status rather than deletion efficiency. The deletion of *Nfkbia* is near complete but restricted to the epithelial compartment; we have previously demonstrated both absence of IκBα protein and a reduction of *Nfkbia* transcripts to below 5% in IECs (*23*).

The absence of an overt spontaneous phenotype in $I\kappa B\alpha^{IEC\ KO}$ mice is therefore not due to incomplete deletion, but rather reflects the tightly regulated, context- and niche-dependent nature of NF-κB activation *in vivo*. Indeed, we have never observed uniform NF-κB activation across all epithelial cells *in vivo*, irrespective of stimulus or readout. Instead, NF-κB activation occurs in a spatially and temporally restricted manner, depending on cell type and microenvironment. This contrasts with *in vitro* systems, where NF-κB activation—assessed by EGFP expression or nuclear p65 translocation—often occurs synchronously in the majority of cells.

Reviewer 4:

This manuscript by Barleben et al. presents a compelling, yet potentially controversial, perspective on the role of NF-κB in colitis. While the pro-inflammatory role of NF-κB in immune cells during colitis development is well-established, the authors provide evidence for a protective function of NF-κB within intestinal epithelial cells (IECs) during the recovery phase, utilizing a combination of mouse models, single-cell RNA sequencing, and patient data. The data showing that IEC-specific NF-κB activation promotes Treg recruitment and "reprogramming" to resolve inflammation is particularly intriguing.
Although the study offers valuable insights, the presentation is hindered by several issues that compromise its clarity and rigor, just an example, The authors listed many experiments and results but did not adequately address them.

We thank the reviewer for the thoughtful summary and for recognizing the novelty and potential impact of defining a protective, epithelial-specific role for NF-κB during colitis resolution. We appreciate the concern regarding clarity and presentation. In response, we have reorganized the manuscript, and include data to further dissect the nature of epithelial NF-κB-driven reprogramming.
We have also revised the text to more clearly articulate how individual datasets, mouse models, scRNA-seq, and patient samples converge to support a coherent mechanistic model. We hope that these changes improve clarity and address the reviewer's concerns.

Major revision
- the authors highlight that "in contrast to the untreated mice, where activated NF-κB localised to Ki67+ stem cells, in recovery activated NF-κB was predominantly detected in CHGA+ enteroendocrine cells"; however, the function of this subset of epithelial cells was

completely overlooked in the manuscript. Does Chromogranin-A involve in the recruitment of Tregs? Or others hormones?

We thank the reviewer for highlighting this important aspect. Indeed, the shift of NF-κB activation toward Chromogranin A$^+$ enteroendocrine cells (EECs) during recovery is intriguing. EECs have the potential to modulate mucosal immune responses via the secretion of immunoactive hormones; however this functional role was not addressed in the present manuscript as it constitutes the focus of an ongoing, dedicated project pursued independently by another member of our group. We briefly discuss some of the findings below.

We found that activation of NF-κB promotes differentiation secretory precursors towards EEC. We analysed different stress responses including high fat diet, short and long-term DSS treatment and examined expression of genes corresponding to unique EEC types. In line with this *IκBα $^{IEC\ KO}$* mice have a higher number of EEC already at baseline. However instead of directing EEC precursors into a specific lineage, EEC cells were arrested at a more immature state, displaying pan EEC markers, such as ChgA and ChgB, but losing expression of differentiated EEC, specifically L cell markers. Although it is intriguing to speculate what kind of immuno-modulatory hormones these immature EEC would produce, because these cells still constitutive about 1% of IEC, we decided not to focus on them in this project.

- The study identifies different IEC subpopulations with distinct NF-κB activity. What are the specific functions of these subpopulations in colitis and recovery, and how do they coordinate their responses?

This is a very important point, and we apologize we did not make it more clear in the original version. We now include a reworked main Figure 1k (New Figure 1k) that specifies functions of NF-κB, derived as before from scRNA analysis, at distinct time points: in homeostasis, in colitis, and in recovery. Epithelial NF-κB switches between different gene sets – driving expression of genes that perform maintenance functions – "Response to Nutrient Levels" (like *Mt3*) and respond to microbiota (for example *Cxcl2* and *Pglyrp1*) in homeostasis. In colitis, on the contrary, the overarching function is defence – "inflammatory response" (via genes like *Ccl22* and *Ccl5*) and leucocyte chemotaxis (for example, *Pdgfb*), which attract leucocytes to the site of damage. Finally in recovery, another distinct NF-κB gene set is enriched for factors that help in epithelial regeneration (e.g., *Egfr* and *Tgm*).

We believe that these distinct gene sets lead to functionally and temporally distinct secretomes of NF-κB – forming its "lexicon". This way, this ancient(*24*) and pivotal transcription factor can coordinate numerous processes depending on stimulus. Although multicellular organisms evolved additional layers of complexity to refine the reaction to exogenous stimuli, we hypothesize that this shift between transcriptomes cemented NF-κB as a multifaceted factor in unicellular organisms that lacked a real immune system.

- The authors state that IEC NF-kB "reprogram" Tregs. However, the molecular mechanisms underlying this reprogramming are not fully elucidated. What specific signaling pathways or molecular interactions mediate this reprogramming of Tregs by IECs?

Our analysis of epithelial-specific secretome (Figure 6a) and also predicted interaction from CellChat (Figure 3c) predicted paracrine signalling as a critical driver of reprogramming. Although our study does not exclude juxtracrine or endocrine signalling, it shows that several paracrine factors produced by intestinal epithelium are both necessary and sufficient for reprogramming. We address the mechanism in depth by first testing select factors we have identified as secreted by epithelium in an NF-κB-dependent manner and then by sequencing Tregs by scRNA-seq to identify the transcriptional phenotype of reprogramming.

We address how select factors contribute to increased proliferation of Tregs in response to a question about reprogramming below.

- The authors emphasize the critical role on epithelial cells of activated NF-κB during the recovery phase; however, they only analysed NF-κB-driven genes during the active phase of UC, where NF-κB was deregulated. What happens to these genes during remission in patients? Is there an intrinsic or extrinsic deregulation of these genes also during the mucosal healing that impact the regeneration of tissue in these patients?

We thank the reviewer for this comment: although the protective effect of epithelial NF-κB manifests in recovery, it is its activation in the acute phase (in Wt mice) or even under homeostasis in the *IκBα* $^{IEC\ KO}$ model, that paves the way for tissue regeneration days later. We would argue NF-κB is deregulated in UC patients, likely through epithelial-intrinsic defects. Our *in vitro* experiments showed that epithelium from UC patients retain IEC-defects in survival and recruitment, even when taken out of the inflammatory context (Figure 7d-k).

It is challenging to recruit patients who are in remission for colonoscopies, however whether specific NF-κB gene signatures can be used as a predictor of response is an intriguing possibility. An indirect indication is the pathogenesis of IBD: IBD patients frequently have genetic predispositions (mutations in NF-κB target genes like *TNFAIP3* or *NOD2* or *NFKBIA*) and therefore these are likely to deregulate signalling even in epithelium (in case of NOD2). Since barrier defects often occur before disease manifests itself (*25, 26*), we speculate that NF-κB will remain deregulated. Successful remission could depend on the ability to target immune cells only and circumvent further deregulation of epithelial NF-κB.

- The authors claim that treatments such as corticosteroids could negatively suppress epithelial NF-κB, thereby impacting the regeneration of the epithelial compartment. As future prospective is it possible to differentiate the suppression of nFkB only on leucocytes where the authors found activation of pro-inflammatory genes? How do the authors reconcile the pro-inflammatory nature of NF-κB with its observed protective role in IECs?

We thank the reviewer for this insightful question. Indeed, our findings raise the important possibility that cell type–specific modulation of NF-κB activity, rather than global inhibition, could improve current IBD therapies. Conceptually, such strategies would aim to suppress pathological NF-κB activation in leukocytes while preserving—or even supporting—protective NF-κB functions in the intestinal epithelium. One potential avenue could involve targeting NF-κB–regulated receptors or effector pathways that are selectively expressed in immune cells but absent from epithelial cells.

With respect to the apparent paradox between the well-established pro-inflammatory role of NF-κB and its protective function in IECs, our data support a context- and cell type-dependent model: in leukocytes, NF-κB predominantly drives inflammatory gene expression, whereas in IECs it coordinates immune-modulatory and regenerative programmes required for mucosal repair. Broad systemic NF-κB suppression, for example by corticosteroids, may therefore inadvertently impair epithelial recovery despite dampening inflammation.

While highly selective or temporally restricted approaches to NF-κB modulation are conceptually attractive and are being explored experimentally, they remain beyond the scope of the current study and are discussed here only as future perspectives.

- While the authors acknowledge previous discrepancies regarding NF-κB's role in IEC apoptosis, a more in-depth discussion of the factors influencing NF-κB's context-dependent functions in IECs would be beneficial.

We fully agree with the reviewer and have expanded the Introduction to more explicitly discuss the factors underlying the context-dependent functions of NF-κB in IECs. A major

source of discrepancy in earlier studies has been the difficulty in disentangling the functions of NF-κB itself from those of its upstream regulators, including IKK and A20. These molecules act upstream of NF-κB but have since been shown to exert NF-κB–independent effects on gene regulation, for example through modulation of mRNA stability (*24*). Consequently, phenotypes observed in models targeting IKK or related signalling components cannot be unequivocally attributed to altered NF-κB transcriptional activity.

Indeed, many prior studies relied on knockout or knock-in strategies targeting IKK or other upstream mediators, and consistent with this, epithelial cells in IKK-deficient mice exhibit increased susceptibility to apoptosis (*27, 28*). In contrast, our approach selectively modulates NF-κB activity itself by deleting its direct inhibitor, IκBα, thereby avoiding interference with upstream signalling pathways (*23*). Similarly, expression of an IκBα super-repressor suppresses NF-κB transcriptional activity without disrupting IKK-dependent functions.

Moreover, we and others have previously shown that under specific cellular states such as senescence, NF-κB activity can become largely IKK-independent and drive a distinct transcriptional programme, including the senescence-associated secretory phenotype (SASP) (*29*). These observations raise the possibility that, within the intestinal epithelium, discrete cellular states—such as senescence—may likewise engage context-specific NF-κB programmes, contributing to the diverse and sometimes opposing outcomes attributed to NF-κB signalling in IECs.

- The authors in the figure Fig 3d report the semantic space analysis reporting in the text: GO analysis revealed that under all three conditions (homeostasis, colitis, and early recovery) genes positively regulating metabolism constituting processes including “lipid metabolism”, “sterol biosynthesis” and “cholesterol biosynthesis” that were suppressed in ΔNIEC mice; Are these pathways involved in the regeneration of the mucosa? What was their expression in IκBa IEC KO mice?

We thank the reviewer for this insightful question. Metabolic pathways in intestinal epithelial cells, particularly lipid and sterol biosynthesis, are indeed indirectly linked to mucosal regeneration, as they provide essential building blocks for membrane formation and energy supply during epithelial proliferation. However, these pathways are not the primary drivers of the regenerative phenotype that distinguishes the model; instead, their suppression likely reflects the altered homeostatic state and transcriptomic shift associated with chronic epithelial NF-κB suppression.

We therefore examined the expression of the individual genes contributing to the ‘lipid/sterol/cholesterol biosynthesis’ GO terms. In ΔN^{IEC} mice, this module was driven by canonical sterol regulators (*Srebf1, Mvd, Mvk, Dhcr24, Scap, Nr1h3, Rxra*), which were suppressed in epithelium. In contrast, these core genes were not differentially expressed in $I\kappa B\alpha^{IEC\ KO}$ mice, and we did not observe a systematic, inverse upregulation of the ΔN^{IEC} metabolic signature. Metabolic changes in the $I\kappa B\alpha^{IEC\ KO}$ model involved a different set of lipid and amino-acid–metabolizing genes (e.g. *Acaca, Nsdhl, Fads2/3/6, Acss2, Acsl1, Gys1, Pck1*), indicating that the sterol-biosynthetic program suppressed ΔN^{IEC} in mice is a model-specific consequence of chronic epithelial NF-κB activation rather than a simple mirror of the regenerative phenotype.

- The long list of downstream genes driven by NF-κB confirms its critical role in several cell compartments, making it quite difficult to identify a unique set responsible of its beneficial and pro-inflammatory effects. It should be relevant to better address this aspect in the manuscript.

We fully agree with the reviewer that multiple epithelial-derived factors contribute to the observed phenotype. In our view, the key conclusion of the study is not that a single cytokine or chemokine is responsible, but rather that epithelial NF-κB orchestrates a coordinated, multifactorial program that both recruits and reprograms immune cells in response to injury. As a proof of principle, we focused on those factors showing the strongest induction and with established roles in T-cell recruitment or functional modulation, to illustrate how epithelial NF-κB–dependent regulation of select molecules can drive colitis resolution. Because this point intersects with comments raised by other reviewers and closely relates to the reviewer's subsequent question, we provide a more detailed explanation in our response to the next comment.

- The authors identify a pro-inflammatory secretome, but the functional significance of individual components within this secretome remains unclear. Which specific cytokines, chemokines, or other secreted factors are most critical for Treg recruitment, activation, or reprogramming?

This is an important question also raised by the other two reviewers and here we separate the answer into two parts: epithelial chemokines for Treg recruitment and epithelial factors for Treg reprogramming.

In the original manuscript we identified several epithelial derived secreted factors that are regulated in an NF-κB-dependent manner (Figure 6a). Since we observed differential recruitment of T cells, and specifically Tregs - these were increased six-fold in mucosa of the untreated $I\kappa B\alpha^{IEC\ KO}$ mice, we focused on secreted factors capable of recruiting lymphocytes. Of the secreted factors, chemokines Ccl22, Ccl5, and Cxcl10 are known as direct targets of NF-κB (bold in the heatmap) and showed a significant increase (Figure 6a). Although all these could potentially recruit macrophages, they, and especially Ccl22, are recognized as central in the context of T cell recruitment (*11-13*).

Of these, Ccl22 showed the strongest induction in an NF-κB and in a colitis-dependent manner (Figure 6a). The only known receptor (so far) for Ccl22 is the C-C Chemokine Receptor 4 (CCR4), which is present on regulatory T cells (Tregs) under homeostasis(*12*) and can be induced on other cells types under stress. Ccl22 is one of the key chemokines for Treg trafficking(*12, 14*). It was recently shown to mediate protective functions via Treg-dendritic cell interactions in DSS-induced colitis by the landmark paper from the David Anz group(*12*). Ccl22 however is also secreted by intestinal epithelial cells (*15*). We observed expression of *Ccl22* in epithelial cells, exclusively in DSS treated mice (New Figure 6c). To determine if the chemokines we identified in the protein array, including Ccl22, can directly recruit Tregs, we performed a transwell assay with isolated primary Tregs from Wt mice. Tregs were stimulated with Ccl22 or Ccl5 (chemokine showing highest induction in DSS Wt and in $I\kappa B\alpha^{IEC\ KO}$ untreated mice) and Cxcl10 (control to recruit Tregs, however weakly induced in $I\kappa B\alpha^{IEC\ KO}$ untreated mice). In comparison to the negative control (medium only) all three potently led to Treg migration (new Figure 6b).

We then focused on factors secreted by epithelium in an NF-κB-dependent manner, which would affect Treg activation or reprogramming. Although there were several factors (New Figure 6a) which were previously shown to reprogram immune cells, including Tregs, we focused on those with the highest induction and that had been previously well characterized in the context of Treg activation. It was not our goal here to identify novel factors that can reprogram Tregs, but rather show as a proof of concept, that epithelium can act as a first responder in damage and through NF-κB activation can both recruit and reprogram Tregs relying on simultaneous activation of expression of a score of factors.

Here we focused on IL-33 and amphiregulin as potential reprogrammers of Tregs. We initially hypothesized that factors present in the medium of $I\kappa B\alpha^{IEC\ KO}$ organoids would

promote proliferation and addition of the responsible factor, would not further boost Treg proliferation (Figure 6d). However, we noticed that factors present in the medium from the Wt had also suppressive functions, which addition of IL-33 was not able to rescue. Although medium from *IκBα* $^{IEC\ KO}$ organoids boosted proliferation, we still could not rule out that factors present there also suppressing growth. Therefore, organoid medium could not be used as a control.

Nonetheless, the results clearly showed that both IL-33 and amphiregulin successfully increased proliferation of Tregs, their survival, as determined by presence of CCR4+ Tregs (New Figure 6d). IL-33 was previously shown to promote differentiation of Treg cells and also to provide a necessary signal for Treg-cell accumulation in inflamed tissues (*30*). More recent reports showed that it thwarts IL-17 production by Tregs and inhibits Th17 differentiation (*31*). Our work bridges previous studies, showing that induced activation of epithelial NF-κB mediates these protective reprogramming functions in Tregs.

Since we have established that Tregs derived from colon of *IκBα* $^{IEC\ KO}$ mice have an enhanced suppressive phenotype, we performed scRNA-seq on colonic versus splenic leucocytes to determine whether how Tregs are reprogrammed and also whether the reprogramming remains local or is systemic (New Figure 6g). Remarkably, epithelial NF-κB activation reprogrammed Tregs not only locally in the colon but also systemically. Among differentially expressed genes, *Gdpd3* (Glycerophosphodiester phosphodiesterase domain–containing 3) was almost exclusively detected in Tregs from *IκBα* $^{IEC\ KO}$ mice (New Fig. 6h and Extended data 6e). Although *Gdpd3* has been mentioned only rarely in the literature, previous work suggests it is associated with memory T-cell features (*9*) and is required for leukaemia stem cell survival (*10*). Based on this, we hypothesized that epithelial NF-κB confers enhanced activation and memory-like properties to Tregs, thereby supporting their regenerative functions. We confirmed presence of Gdpd3 also on protein level by immunofluorescence (New Figure 6j and k). Here we detected a significant increase in Gdpd3+ CD4+ cells in *IκBα* $^{IEC\ KO}$ mice, confirming metabolic rewiring in Tregs driven through NF-κB.

In summary, we show clear reprogramming of Tregs via epithelial NF-κB using the three methods: Tregs from *IκBα* $^{IEC\ KO}$ mice show increased activation, evidenced by increased CCR4 levels and increased IL-10 secretion. We furthermore demonstrated that they possess a distinct transcriptional signature with increased expression and production of Gdpd3+, previously proposed a marker of memory T cells.

- Although the authors focused on epithelial-immune interactions. What is the role of mesenchymal cells in modulating IEC NF-κB signaling and its functions in colitis?

Mesenchymal cells are increasingly recognized as key modulators of epithelial NF-κB activity and epithelial function during colitis. Fibroblasts provide essential trophic cues,such as niche factors (WNTs, RSPOs), BMP antagonists, and EGFR ligands that influence both baseline epithelial turnover and the ability of IECs to engage NF-κB–driven repair programs. During inflammation, stromal cells also produce cytokines and chemokines (e.g., IL-6, CXCL12) that can either potentiate epithelial NF-κB activation or reshape its downstream outputs. Disruption of this axis, either stromal or epithelial, has been shown to impair barrier repair and exacerbate colitis, underscoring the importance of this compartment in shaping IEC NF-κB functions.

We performed scRNA sequencing on epithelium and fibroblasts from Wt mice and *IκBα* $^{IEC\ KO}$ mice. Differential analysis yielded the following genes are differentially upregulated in colonic fibroblasts of *IκBα* $^{IEC\ KO}$ mice: *Zc2hc1a, Clu, Kcnt2, Colgalt2, Pbx1, Pkhd1l1, Myo16, Zfp521, Ptpn14, Mmp16, Sema3e, Asic2, Gpc6, Zfpm2, C7, Astn2.* However further analysis

by Reactome Pathways to identify top GO terms, only yielded one significant GO term "Terminal Pathway of Complement" ($p = 0.001$) with two genes *Clu* and *C7*.

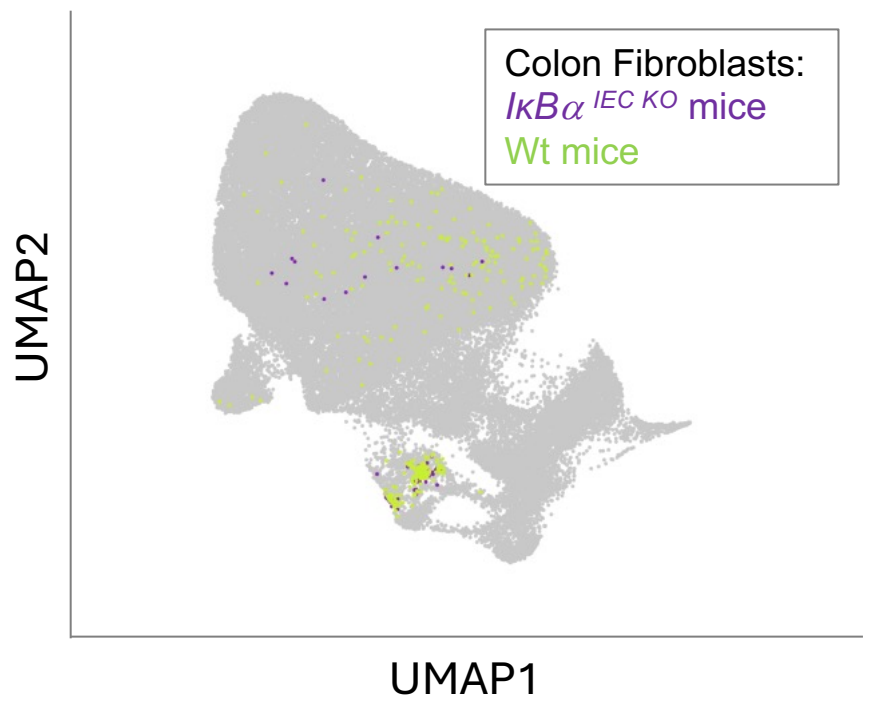

It is intriguing to speculate that epithelium likewise changes the phenotype of the stroma and potentially reprograms fibroblasts toward a complement-associated, damage-responsive state. Although the transcriptional changes we observed were modest, the selective upregulation of *Clu* and *C7* suggests that epithelial NF-κB activation may prime mesenchymal cells to participate in terminal complement activation, a pathway increasingly recognized as a modulator of tissue inflammation and repair.

Minor revisions

Generally, The study presents interesting findings, but the manuscript could benefit from greater focus and conciseness. The extensive list of genes, while informative, appears to divert attention from the main theme of the research. A more streamlined approach would enhance the clarity and impact of the paper, ensuring that the core message is not lost amidst the details.

We thank the reviewer for this feedback and attempted to make the manuscript more concise.

- The results related to Extended figure1 needs to be more integrated into the text as well as the EGFP construct more explained. The authors showed the activation of NF-κB in different tissues, but finally they focused on the colon. Is it a control to demonstrate that system works or what?

That is correct. We would like to show that this construct can be used for other organs. We explain the construct more fully in the new legend.

- The effects of NF-κB suppression and activation in colitis should be reported in comparison in the same graph to better appreciate its role on epithelial cells.

Unfortunately, this is not possible because after discovering the hypersensitivity of mice with suppressed NF-KB we switched to using 1.5% DSS with those mice and appropriate controls. The experiments performed with *IκBα* $^{IEC\ KO}$ mice were with full dosage DSS.

- How was calculated the "colitis score"? Is it based exclusively on the histology?

This is a well-established method previously published by our collaborators, Erben et al., 2014. We include in this now in the methods and apologize for the initial omission.

- In Extended figure1 d there is presented the body weight loss as % starting weight, whereas in figure 2 as weight in grams. Why this different representation? Additionally, looking at DSS impact on colits, DSS at 3% appears less strong that 1.5 % in WT, why? This variability could impact on the gene expression profiling.

We generally show weight as % increase or decrease, however with mice where the initial weight between groups is different, we showed weight in grams, so that that difference too would be apparent.

The effects for the Wt 1.5 % vs 3% at day 5 showed a large distribution of weight and was not significant. We agree that this variability could create many false negatives, however

this large variability was observed many times, especially in recovery. We currently cannot ascribe it to any specific factor.

- Why for quantification of isolate lymphoid follicles reported in Extended data 2b-c was used DAPI staining of colon samples and not Haematoxylin & Eosin staining or a CD45 staining in immunohistochemistry?

We have now use H&E staining to quantitate ILFs. These data support our previous conclusion, however H&E staining makes it more challenging to accurately identify ILF in colon. DAPI, on the other hand, makes ILF stand out (due to density of the nuclei immune cells) and therefore was chosen here for the figure. Below however we now quantification also from H&E in the figure For Reviewers only – C.

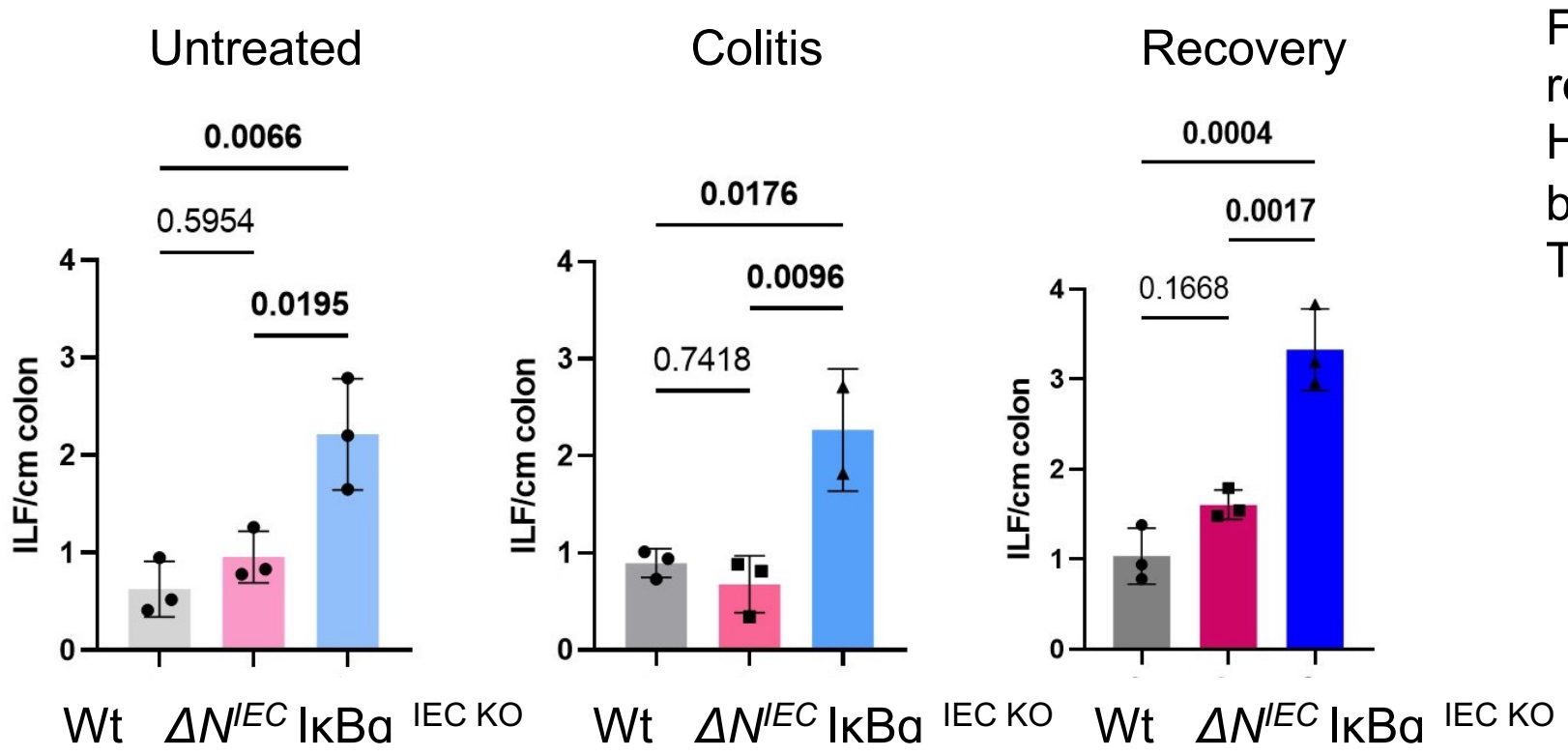

For Reviewers only C: requantification of ILF using H&E. Significance determined by one way ANOVA, including Tukey's correction.

- Figure 1a: To improve the clearness of scheme which is confounding.

We have changed the diagram.

- Figure 1e: it is suggested to link with line or reported in the low magnification which one the correspondent ROI

We have added a better visible line.

- Figure 1j: To reduce the font size because there is a partial overlapping the row labels (gene names).

We have changed that.

- Figure 1k: the author should consider the idea to change the type of graph for the semantic space analysis because may be confusing.

We have replaced this graph and simplified it.

- Extended data 1d: To check the correctness of days reported in text and which reported in the graph. In the text is reported that there is a delay in the body weight loss from day 4 but in the graph is reported the day 5.

Thank you for spotting it. We have changed the text.

- Extended data 1g-h-i: The authors report the p value no clearly indicating which is the comparison considered. Lack a proper description in the text and in the footnote of the figure.

We have added this data to the figure legend. We now show individual p values for multiple comparisons.

- Extended data 1k: To add the label of the scale bar that is missing.

We added the label.

- Figure 2m-n: To reduce the size font of X axis label.

We have changed the figure to avoid the text overlap.

- Extended data 2c-e: To reduce the size font of X axis label.

We have changed the width of the X axis to improve the layout.

- Figure 3d: To modify the graph size to avoid the overlapping.

We have fixed this issue.

- Figure 4(a-d): To reduce the size font of X axis label.

We have now performed flow cytometry and replaced these panels (New Figures 4a-d).

- Figure 4g: To reduce the row labels to improve the readiness

We reduce it. This is now figure 6a.

- Extended data 4: The authors may better report the correspondence between cellular type and marker used to immunodetected.

We now used immunophenotyping by flow cytometry and removed these panels.

- Figure 5b: The author may add the untreated DSS and healthy mice to the graph, as already reported for the colitis score (Fig. 5d) and ki67% of epithelial cells (Fig. 5f).

We have added it.

- Fig 6d-h: To reduce the row labels to improve the readiness

We have improved it.

The author should be consistent in the use of American English or UK English. For example:
- the use of “leukocyte” and “leucocyte” across the paper

- the use of “visualization” and “visualisation”.

- The use of signaling and signalling

The style was changed to English UK.

Other comments:
- Line 151: "Furthermore, we demonstrated that in IEC distinct NF-KB gene sets are activated during initiation and progression of colitis and recovery." The sentence would read better as: "Furthermore, we demonstrated that distinct NF-κB gene sets in IEC are activated during initiation, progression, and recovery of colitis."

Thank you for this suggestion. We have changed it.

- Line 186: "After DSS treatment, the epithelial barrier collapse was evidenced by a decrease in markers of both tight junctions and adherens junctions, Zonula occludens (ZO1) and of CDH1 respectively." The sentence is a bit convoluted.

We have modified the sentence. The new sentences reads "After DSS treatment, collapse of the epithelial barrier was reflected by a reduction in markers of both tight junctions and adherens junctions, namely zonula occludens-1 (ZO-1) and E-cadherin (CDH1)."

- Line 288: "Strikingly, in IkB IEC KO mice under homeostasis, we observed over six-fold increase in mucosal FoxP3+ Tregs, however no further induction occurred in colitis (Fig. 4e and f), suggesting these are likely recruited via the same NF-KB pathway." the sentence structure can be improved for clarity.

We modified this sentence for clarity. The new sentence is "Strikingly, *IκBα* $^{IEC\ KO}$ mice showed a more than sixfold increase in mucosal $FoxP3^{+}$ Tregs under homeostasis, but no additional induction during colitis (Fig. 4e,f), consistent with recruitment through a shared NF-κB–dependent mechanism."

- Line 452: "Histology and immunofluorescent staining, including Multiplex IF: H&E, immunofluorescence, and multiplex stainings were performed as described previously 10, 24, 41 Colonic tissues for multiplex staining were incubated in 10% donkey serum (Sigma-Aldrich Cat. D9663 or S30-M or Abcam Cat. ab7475) and stained with DAPI (Sigma-Aldrich Cat. D9542) together with secondary antibodies (Supplemental Table 8)." This is a run-on sentence and should be broken down for clarity. Also, "immunofluorescent" should be "immunofluorescence"

Thank you. We changed this.

We thank the reviewer for the very helpful and thorough reading. The changes recommended improved the manuscript and we hope made the main message stronger and more clear.

Bibliography for Point by Point

1. Y. Hu *et al.*, Th17/Treg imbalance in inflammatory bowel disease: immunological mechanisms and microbiota-driven regulation. *Front Immunol* **16**, 1651063 (2025).
2. J. N. Clough, O. S. Omer, S. Tasker, G. M. Lord, P. M. Irving, Regulatory T-cell therapy in Crohn's disease: challenges and advances. *Gut* **69**, 942-952 (2020).
3. C. Voskens *et al.*, Autologous regulatory T-cell transfer in refractory ulcerative colitis with concomitant primary sclerosing cholangitis. *Gut* **72**, 49-53 (2023).
4. C. Mottet, H. H. Uhlig, F. Powrie, Cutting edge: cure of colitis by CD4+CD25+ regulatory T cells. *J Immunol* **170**, 3939-3943 (2003).
5. E. Elinav, T. Waks, Z. Eshhar, Redirection of regulatory T cells with predetermined specificity for the treatment of experimental colitis in mice. *Gastroenterology* **134**, 2014-2024 (2008).
6. S. Bank *et al.*, Associations between functional polymorphisms in the NFkappaB signaling pathway and response to anti-TNF treatment in Danish patients with inflammatory bowel disease. *Pharmacogenomics J* **14**, 526-534 (2014).

7. A. Chaudhry *et al.*, Interleukin-10 signaling in regulatory T cells is required for suppression of Th17 cell-mediated inflammation. *Immunity* **34**, 566-578 (2011).
8. D. Sugiyama *et al.*, Anti-CCR4 mAb selectively depletes effector-type FoxP3+CD4+ regulatory T cells, evoking antitumor immune responses in humans. *Proc Natl Acad Sci U S A* **110**, 17945-17950 (2013).
9. T. Kaji *et al.*, CD4 memory T cells develop and acquire functional competence by sequential cognate interactions and stepwise gene regulation. *Int Immunol* **28**, 267-282 (2016).
10. K. Naka *et al.*, The lysophospholipase D enzyme Gdpd3 is required to maintain chronic myelogenous leukaemia stem cells. *Nat Commun* **11**, 4681 (2020).
11. J. Montane *et al.*, Prevention of murine autoimmune diabetes by CCL22-mediated Treg recruitment to the pancreatic islets. *J Clin Invest* **121**, 3024-3028 (2011).
12. M. Rapp *et al.*, CCL22 controls immunity by promoting regulatory T cell communication with dendritic cells in lymph nodes. *J Exp Med* **216**, 1170-1181 (2019).
13. A. W. Mailloux, M. R. Young, NK-dependent increases in CCL22 secretion selectively recruits regulatory T cells to the tumor microenvironment. *J Immunol* **182**, 2753-2765 (2009).
14. A. Iellem *et al.*, Unique chemotactic response profile and specific expression of chemokine receptors CCR4 and CCR8 by CD4(+)CD25(+) regulatory T cells. *J Exp Med* **194**, 847-853 (2001).
15. M. C. Berin, M. B. Dwinell, L. Eckmann, M. F. Kagnoff, Production of MDC/CCL22 by human intestinal epithelial cells. *Am J Physiol Gastrointest Liver Physiol* **280**, G1217-1226 (2001).
16. B. Li *et al.*, Impaired Wnt/beta-catenin pathway leads to dysfunction of intestinal regeneration during necrotizing enterocolitis. *Cell Death Dis* **10**, 743 (2019).
17. D. Zhao *et al.*, T helper cell 17/regulatory T cell balance regulates ulcerative colitis and the therapeutic role of natural plant components: a review. *Front Med (Lausanne)* **11**, 1502849 (2024).
18. D. Y. Honing, R. M. Luiten, T. R. Matos, Regulatory T Cell Dysfunction in Autoimmune Diseases. *Int J Mol Sci* **25**, (2024).
19. L. Yang, G. Wang, H. Xia, Molecular mechanism for impaired suppressive function of Tregs in autoimmune diseases: A summary of cell-intrinsic and cell-extrinsic factors. *J Cell Mol Med* **24**, 11056-11063 (2020).
20. Y. Gu *et al.*, Immune microniches shape intestinal T(reg) function. *Nature* **628**, 854-862 (2024).
21. J. Xu *et al.*, New Insights Into the Epigenetic Regulation of Inflammatory Bowel Disease. *Front Pharmacol* **13**, 813659 (2022).
22. D. Wang *et al.*, Macrophage Subset Expressing CD169 in Peritoneal Cavity-Regulated Mucosal Inflammation Together with Lower Levels of CCL22. *Inflammation* **40**, 1191-1203 (2017).
23. N. Mikuda *et al.*, Deficiency in IkappaBalpha in the intestinal epithelium leads to spontaneous inflammation and mediates apoptosis in the gut. *J Pathol* **251**, 160-174 (2020).
24. T. D. Gilmore, F. S. Wolenski, NF-kappaB: where did it come from and why? *Immunol Rev* **246**, 14-35 (2012).

25. E. Martini, S. M. Krug, B. Siegmund, M. F. Neurath, C. Becker, Mend Your Fences: The Epithelial Barrier and its Relationship With Mucosal Immunity in Inflammatory Bowel Disease. *Cell Mol Gastroenterol Hepatol* **4**, 33-46 (2017).
26. I. Atreya, R. Atreya, M. F. Neurath, NF-kappaB in inflammatory bowel disease. *J Intern Med* **263**, 591-596 (2008).
27. A. Nenci *et al.*, Epithelial NEMO links innate immunity to chronic intestinal inflammation. *Nature* **446**, 557-561 (2007).
28. F. R. Greten *et al.*, IKKbeta links inflammation and tumorigenesis in a mouse model of colitis-associated cancer. *Cell* **118**, 285-296 (2004).
29. M. Kolesnichenko *et al.*, Transcriptional repression of NFKBIA triggers constitutive IKK- and proteasome-independent p65/RelA activation in senescence. *EMBO J* **40**, e104296 (2021).
30. C. Schiering *et al.*, The alarmin IL-33 promotes regulatory T-cell function in the intestine. *Nature* **513**, 564-568 (2014).
31. E. Pastille *et al.*, The IL-33/ST2 pathway shapes the regulatory T cell phenotype to promote intestinal cancer. *Mucosal Immunol* **12**, 990-1003 (2019).

Point by Point Response

REVIEWER COMMENTS

Reviewer #1 (Remarks to the Author):

The authors are greatly appreciated for undertaking an extensive series of additional experiments and for making a genuine effort to address all of the concerns. It is well recognized that analyzing NF-κB signaling—whose activity is highly dynamic—as well as complex intercellular interactions and the multifactorial functions of the secretome presents substantial technical and conceptual challenges.

Nevertheless, it remains difficult to conclude that all concerns have been fully and satisfactorily addressed. The remaining critical issues can be distilled into the following three major categories, excluding those that have been resolved through the additional experiments.

We thank the reviewer for the thoughtful and constructive assessment of our revised manuscript and for recognizing the substantial experimental and analytical effort invested in addressing the prior concerns. We fully agree that dissecting the dynamics of NF-κB signalling and the functional complexity of epithelial–immune secretome interactions is technically and conceptually challenging, and we appreciate the reviewer's acknowledgement of this.
With the present revision, we have performed additional analyses, expanded the mechanistic clarification, and further strengthened the functional validation in the key areas highlighted. We hope that these new data and clarifications now adequately address the remaining concerns outlined below and bring the manuscript to a level that meets the reviewer's expectations. We have responded point-by-point and clearly indicated all new experiments, analyses, and text changes in the revised version.

1. Treg reprogramming by the epithelial NFκB–dependent secretome
Major 1: The central premise of this manuscript is that chemokines contained within the epithelial NFκB–dependent secretome recruit Tregs to the epithelial niche, where they are subsequently reprogrammed. However, there is insufficient direct evidence to support the notion that downstream factors of NFκB are responsible for reprogramming Tregs. In the experiment shown in Figure 6d, does the culture supernatant from IκBαKO IEC cells increase the suppressive capacity, IL-10 production capacity and Gdpd3 expression, in addition to the expression of CCR4 and the proliferative capacity of Tregs?

We thank the reviewer for this important comment. We agree that distinguishing between NF-κB - dependent epithelial secretome effects on Treg recruitment versus functional reprogramming is essential. Our central conclusion is that the epithelial NF-κB - dependent secretome promotes recruitment and phenotypic adaptation of Tregs; in the revised manuscript we have taken care to more precisely define the level of recruitment via CCL22 and also of functional reprogramming. Additional experiments pertaining to CCL22 are described as part of Major Point 5 below.

In response to the point about reprogramming, we have extended and clarified the analysis of Tregs exposed to culture supernatant from *IκBα* $^{IEC\ KO}$ cells (Fig. 6d and new data in the

revised version). Specifically, we now include additional functional and molecular readouts. We show that, beyond increased Ccr4 expression and proliferative capacity, exposure to *IκBα* $^{IEC\ KO}$ supernatant leads to increased expression of Gdpd3 in Tregs (new main Figure 6m).

We have previously shown that Tregs derived directly from the *IκBα* $^{IEC\ KO}$ animals show dramatically increased IL-10 secretion (Fig. 6g). In this experiment, freshly isolated Tregs were cultured for only 6 hours prior to ELISA analysis, thereby minimizing potential *ex vivo* reprogramming effects.

To test whether Wt Tregs could similarly acquire enhanced IL-10 production following exposure to epithelial-derived factors, we cultured Wt Tregs in the presence of IL-2 and subsequently added conditioned medium from *IκBα* $^{IEC\ KO}$ or Wt colonoids. After four days, IL-10 secretion was assessed by ELISA. Under these conditions, we did not detect differences in IL-10 production (Fig. A). Similar to our observations in the *in vitro* suppression assay, which also requires extended *ex vivo* culture, we found that prolonged culture conditions reduced the suppressive phenotype of Tregs. We therefore interpret these results to indicate that extended *ex vivo* culture and cytokine exposure can override the endogenous Treg programming present in freshly isolated cells, limiting the suitability of long-term co-culture assays for capturing this phenotype.

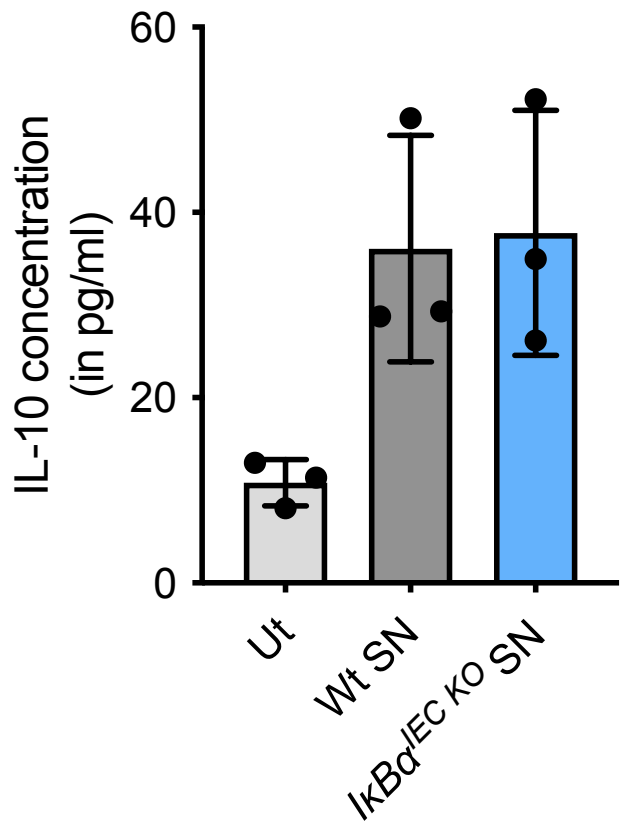

Figure A: ELISA performed On primary Tregs isolated from Wt mice. Tregs cultured as before (Extended Data 6d – in Treg baseline medium + 6 ng/ml IL-2 over four days in the presence of organoid conditioned medium (SN) from Wt or IκBα $^{IEC\ KO}$ organoids.

Importantly, the only genetic alteration in the *IκBα* $^{IEC\ KO}$ mice is intestinal epithelial-restricted activation of NF-κB. We therefore propose that the observed Treg phenotypes arise downstream of epithelial NF-κB signaling. Consistent with this model, epithelial-derived factors alone are sufficient *ex vivo* to induce aspects of Treg phenotypic adaptation, including increased Ccr4 and Gdpd3 expression.

At the same time, we cannot exclude that part of the transcriptional reprogramming observed *in vivo* occurs through indirect mechanisms. IEC-derived factors may first reshape the local immune microenvironment—for example by activating dendritic cells or macrophages that subsequently provide secondary signals to Tregs. In addition, epithelial chemokines may alter Treg localization within specific tissue niches, where exposure to stromal cues, cytokines (e.g., IL-33, TGF-β, or IL-2), or metabolic signals could further influence Treg function. Finally, systemic immune adaptations resulting from sustained epithelial NF-κB activation could contribute to transcriptional differences observed in splenic Tregs. Thus, while our data support a model in which the epithelial NF-κB–dependent secretome plays a key role in Treg recruitment and conditioning, both direct epithelial signals and secondary microenvironmental interactions may contribute to the full extent of Treg reprogramming observed *in vivo*.

We have revised the Results section to explicitly distinguish (i) chemokine-mediated recruitment signals from (ii) secretome-driven functional conditioning effects, and we now more clearly define the scope of the mechanistic conclusions. Together, we hope these additional data and clarifications address the reviewer’s concern regarding evidence for NF-κB - dependent secretome-mediated Treg reprogramming.

Major 2: Although the authors emphasize the importance of Treg reprogramming within the epithelial niche, there appears to be a significant discrepancy between this and the observation that Tregs from the spleen and mesenteric lymph nodes (MLNs) exhibit strong suppressive capacity and elevated Gdpd3 expression. A clear, mechanistic explanation of this apparent contradiction is needed.

We thank the reviewer for raising this important point. We agree that the observation of enhanced suppressive capacity and elevated Gdpd3 expression in Tregs isolated from spleen and mesenteric lymph nodes requires clarification in the context of epithelial niche–dependent reprogramming.

Our current interpretation is that epithelial NF-κB activation initiates Treg conditioning within the intestinal microenvironment, but that aspects of this phenotype can subsequently be maintained or disseminated systemically. Several mechanisms could explain this observation.

First, Tregs that are primed or reprogrammed within the intestinal niche may recirculate and populate secondary lymphoid organs such as the spleen and MLNs while retaining aspects of their transcriptional and functional program. Such migration of tissue-experienced Tregs to lymphoid compartments has been described in multiple contexts. A recent study using photoconversion to study colon egress, showed that after 24h hours less than 40% of original Treg population remained in the gut (*1*). We therefore analysed Gdpd3+ versus the Gdpd3- Tregs in spleen (regardless of genotype) (New Extended Data 6f). *Ccr9,* a key gut homing receptor (*2*), was enriched in Tregs in the *IκBα* $^{IEC\ KO}$ mice and importantly also in Gdpd3+ but not in Gdpd3- Tregs. A similar trend could be observed for another gut homing marker, *Gpr15*. These observations are consistent with the presence of intestinally conditioned Tregs that have recirculated from the gut to secondary lymphoid organs while retaining elements of their tissue-imprinted transcriptional program.

Second, epithelial NF-κB activation alters the intestinal immune microenvironment and cytokine milieu, which may indirectly influence Treg programming in draining lymphoid organs. For example, antigen-presenting cells conditioned in the intestine may migrate to MLNs and spleen and provide secondary signals that reinforce Treg activation or differentiation.

Third, systemic factors induced downstream of epithelial NF-κB signaling, including circulating cytokines or metabolites, could contribute to broader immune modulation and influence Treg states beyond the intestinal tissue itself.

Consistent with these possibilities, our data support a model in which epithelial NF-κB signaling acts as the initiating driver of Treg recruitment and conditioning, while the resulting Treg phenotype can be maintained and detected in both tissue and lymphoid compartments.

We have clarified this point in the revised discussion under limitations of the study, and now explicitly distinguish between the site of initial conditioning and the distribution of the resulting Treg phenotype across immune compartments.

Major 3: Functional assays continue to rely on MLN-derived Tregs. Although the recovery of T cells from the colonic lamina propria of non-colitic mice is recognized as technically challenging, there are precedents for the use of whole CD4$^+$ T cells in this context (Makita S et al., J Immunol. 2007;178(8):4937–4946. doi:10.4049/jimmunol.178.8.4937). At a minimum, the limitations of the current experimental system should be explicitly discussed in the manuscript.

We thank the reviewer for highlighting this important point and for drawing our attention to the study by Makita et al. We agree that functional assays using colonic lamina propria–derived Tregs would represent an ideal experimental system to directly assess tissue-conditioned Treg function.
In response to this suggestion, we attempted twice to isolate Tregs from the colonic lamina propria using the method described in the cited study. However, the final yield was low (approximately 20,000–40,000 cells per preparation) and cell viability was variable. Because several of our functional assays require substantially higher cell numbers (up to 10^6 cells), these yields were insufficient to reliably perform the required experiments. As the original publication does not report the expected T cell yield using this protocol, we contacted the corresponding author on 17.02.2026 to inquire about typical recovery numbers but have not yet received a response.
In the current study, we therefore relied on spleen - derived Tregs, which are functionally linked to the intestinal immune compartment and can be recovered in sufficient numbers for functional assays. Importantly, our data show that spleen - derived Tregs from the *IκBα* $^{IEC\ KO}$ mice retain enhanced suppressive capacity when analysed without prolonged *ex vivo* culture, supporting the conclusion that epithelial NF-κB activation induces a Treg program that is maintained beyond the intestinal tissue itself. In this context, the adoptive transfer experiments provide proof-of-principle that epithelial NF-κB activation initiates Treg conditioning that can subsequently be detected systemically.
Nevertheless, we agree with the reviewer that functional assays using colonic lamina propria - derived Tregs would provide an even more direct assessment of tissue-conditioned Treg function. We have therefore explicitly acknowledged this limitation in both the Results and Discussion sections of the revised manuscript.

Major 4: The absence of detectable differences in trafficking, as determined by FACS analysis, is a major concern. While the authors attribute this to contamination from isolated lymphoid follicles (ILFs), if the number of ILF-associated Tregs is comparable between IkBaIEC KO and WT mice, a difference should still be observable in the presence of such contamination. Are ILF Tregs increased in WT mice? If so, what evidence supports this claim?

We thank the reviewer for raising this important concern. To address this point, we reanalysed the flow cytometry data using *total* cell numbers normalized to tissue weight (cells per gram of colon), as was also suggested by the Reviewer 3 (Supplemental Figure 2). This analysis also revealed shifts in the composition of other immune populations. In particular, we observe a reduction in TCRβ$^+$ T cells and Ly6G$^+$ neutrophils in *IκBα* $^{IEC\ KO}$ mice. When Tregs were previously quantified as FoxP3$^+$ cells within the CD4$^+$ T cell compartment, these compositional changes influenced the apparent frequency of Tregs because the total CD4$^+$ population is reduced in the *IκBα* $^{IEC\ KO}$ mice.
Consistent with this interpretation, our scRNA-seq analysis of CD45$^+$ immune cells shows a similar trend: while the T cells are reduced in *IκBα* $^{IEC\ KO}$ animals, the abundance of Tregs remains largely stable, accompanied by a pronounced reduction in neutrophils and an increase in B cells (new Extended data 4j). These shifts in immune composition explain why Treg frequencies calculated within the CD4$^+$ compartment appear elevated despite more modest changes in absolute Treg numbers. These data strengthen our original hypothesis that epithelial NF-κB shapes the immune cell compartment.
Thus, our revised analysis indicates that epithelial NF-κB activation is associated with both a *relative* enrichment of Tregs within the immune compartment and a broader remodelling of intestinal immune populations, particularly affecting neutrophils and conventional T cells. Importantly, the functional assays demonstrate that Tregs from *IκBα* $^{IEC\ KO}$ mice display enhanced suppressive capacity, indicating that changes in Treg function, in addition to changes in immune composition, contribute to the phenotype.
We have removed the section about ILF Tregs from the manuscript.

We have now incorporated these revised analyses and clarifications into the manuscript and updated the Results section accordingly.

Major 5: Although the importance of CCL22 among the chemokines in the secretome is emphasized, Figure 6A does not show significant differences between WT-col, IkBaIEC KO-ut and -col. Furthermore, Figure 6C suggests that CCL22 is also expressed in WT-ut samples. More rigorous and quantitative analyses are therefore required.

We thank the reviewer for raising this point and agree that the interpretation of the CCL22 data required clarification.
In Figure 6A, CCL22 secretion is very low in the WT untreated condition, where NF-κB activity is at basal levels. In contrast, CCL22 secretion increases more than ten-fold in all three conditions in which NF-κB is activated (WT-colitis, $I\kappa B\alpha^{IEC\ KO}$ untreated, and $I\kappa B\alpha^{IEC\ KO}$ colitis). These results are consistent with the NF-κB activation kinetics shown in Figure 1 and indicate that CCL22 is robustly induced under conditions of epithelial NF-κB activation. We therefore focused on CCL22 because it was among the chemokines most strongly and consistently upregulated across all NF-κB - activated conditions.
Regarding Figure 6C, we apologize for the confusion. The CCL22+ cells visible in the WT untreated sections are immune and not epithelial cells. Leukocytes are known to produce CCL22 under steady-state conditions, and the presence of these uniformly stained immune cells confirms that the staining procedure worked as expected. In contrast, epithelial CCL22 expression is observed specifically in Wt colitis and in $I\kappa B\alpha^{IEC\ KO}$ tissues (both untreated and colitis conditions). To clarify this point, we have revised the figure and now mark immune cells with black arrows to distinguish them from epithelial cells (larger purple arrows) – (modified Figure 6c).

To further strengthen our conclusion, we now provide additional functional evidence. Specifically, we cultured colonoids derived from $CCL22^{-}/^{-}$ mice and stimulated them with LPS, which we and others have previously shown to robustly activate NF-κB. We then performed transwell migration assays using primary Tregs from Wt mice, exposed to supernatants from Wt or $CCL22^{-}/^{-}$ colonoids, either untreated or stimulated with LPS (New Figure 6d). Importantly, while LPS stimulation of Wt colonoids strongly promoted Treg migration, this effect was completely absent when Tregs were exposed to supernatants from $CCL22^{-}/^{-}$ colonoids, demonstrating that epithelial-derived CCL22 is important NF-κB–dependent Treg recruitment.

3. Relevance of the mouse model to ulcerative colitis

Major 6: With regard to the number and function of Tregs, the claim of 'exhaustion' appears to be inconsistent with the findings in the mouse model. What is the status of Gdpd3 and CCR4 expression, which were observed in the mouse studies? Alternatively, do Tregs from ΔNIEC mice exhibit an exhaustion-like phenotype? Clarification on this point is essential.

We thank the reviewer for raising this important point. In our point-by-point response we speculated that Treg exhaustion might contribute to the observed phenotype. However, we agree with the reviewer that we currently do not have sufficient experimental evidence to support this hypothesis. Importantly, this concept was not included in the manuscript itself, and we therefore consider this question beyond the scope of the present study and more suitable for future investigation.

To address the reviewer's question regarding the status of CCR4 and GDPD3 expression in human disease, we analysed publicly available scRNA-seq datasets from patients with ulcerative colitis (UC) and compared them with non-IBD controls (GSE116222).
In our mouse model, we show that Gdpd3 is induced in Tregs under conditions of epithelial NF-κB activation. We therefore asked whether a similar induction occurs in UC. However, in contrast to the controlled NF-κB activation in our mouse model, UC is characterized by dysregulated rather than sustained epithelial NF-κB activation, as discussed in the manuscript. Consistent with this distinction, despite equivalent number of Tregs, GDPD3 expression was decreased in UC samples (Figure B).

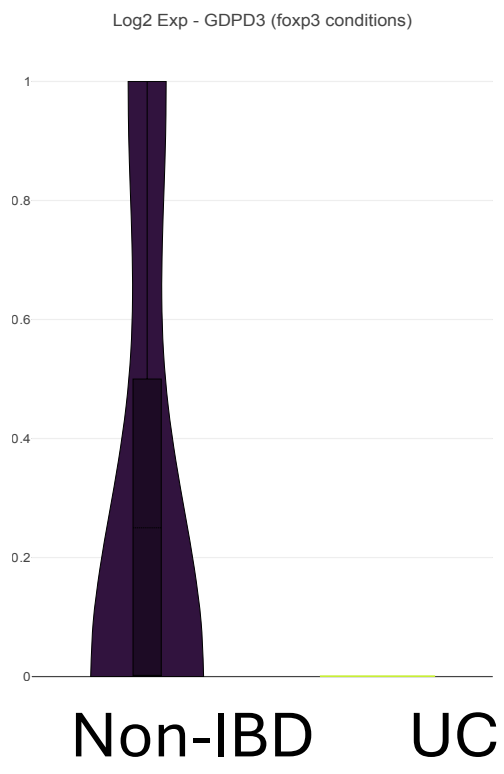

Figure for Reviewer B: Analysis of GDPD3 in FOXP3+ cells in non-IBD vs UC patient samples -- inflamed colon. (from GSE116222). Log2 Exp shown.

It should be noted, however, that GDPD3$^+$ Tregs are relatively rare even in WT samples, which limits the strength of conclusions that can be drawn from currently available datasets. A more definitive assessment of GDPD3 regulation in human disease would likely require a setting with well-defined epithelial NF-κB activation as a positive control, such as in acute infectious colitis, where epithelial NF-κB signalling is robustly induced.

Staining of GDPD3 in UC versus non-IBD biopsies revealed a decrease in a number of GDPD3+ CD4+ cells in UC (Figure C), however as mentioned above, a positive control would be required to definitely determine the expected number.

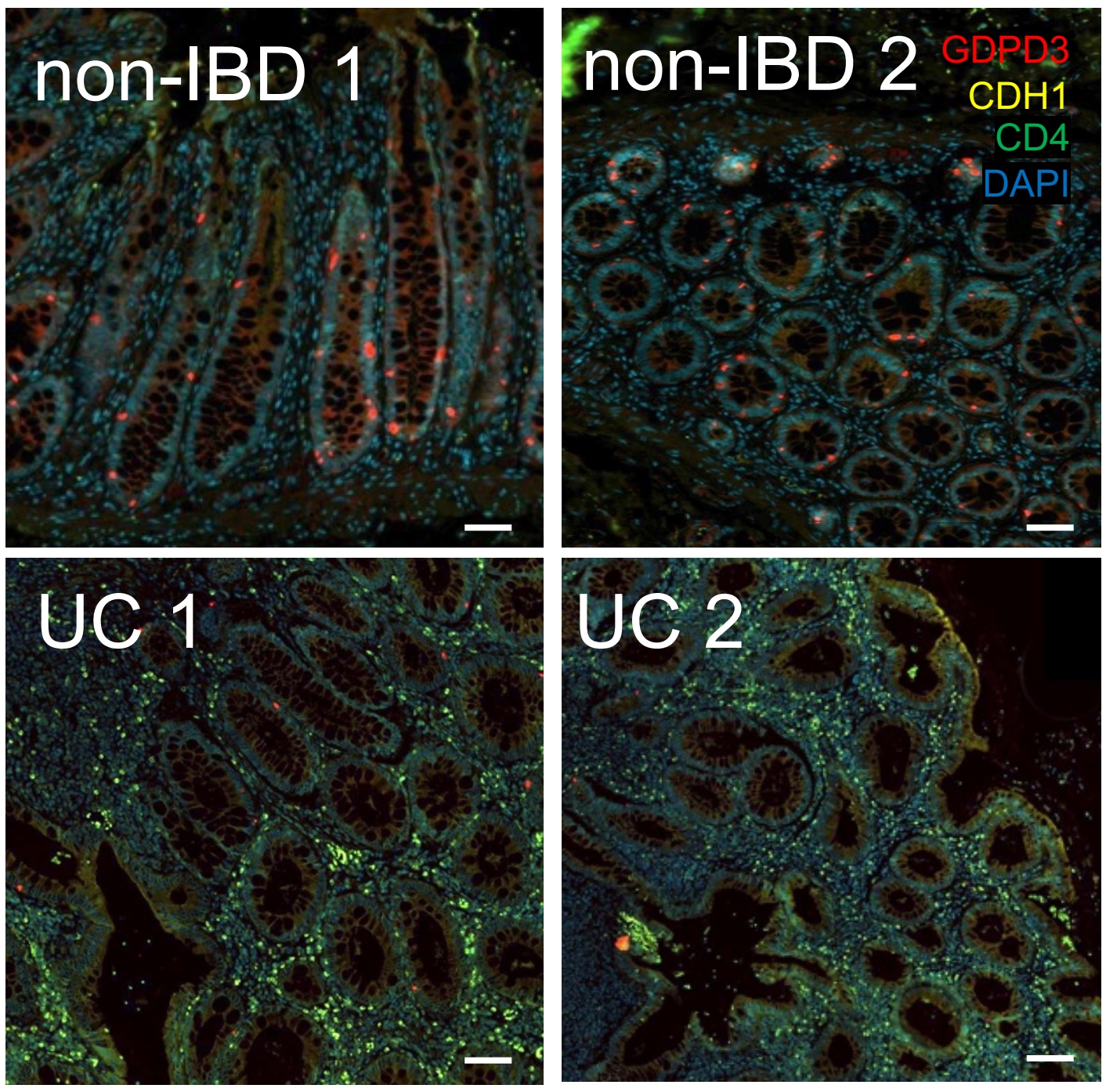

Figure C: IF of GDPD3 in UC (patient 1 and 2) versus "healthy" – non- IBD patient samples 1 and 2. Representative images shown. Scale bar: 50 μm.

With respect to CCR4, several studies have examined its expression in UC and reported reduced CCR4 expression in CD4$^+$ T cells, including CD4$^+$CD25$^+$ populations(*3*). Similarly, another study reported no increase in CCR4$^+$ cells in UC patient colon biopsies compared with healthy controls(*4*). In the scRNA-seq datasets available to us, CCR4 expression could not be robustly analysed due to the very low number of CCR4$^+$ cells detected (typically fewer than ~10 cells per dataset).
Taken together, these analyses suggest that the CCR4/Gdpd3 program observed in our mouse model of epithelial NF-κB activation is not recapitulated in UC, consistent with the notion that UC represents a state of dysregulated inflammation rather than controlled epithelial NF-κB activation.

We thank the reviewer for raising these points and hope we now have sufficiently addressed and clarified them.

Reviewer #2 (Remarks to the Author):

I co-reviewed this manuscript with one of the reviewers who provided the listed reports. This is part of the Nature Communications initiative to facilitate training in peer review and to provide appropriate recognition for Early Career Researchers who co-review manuscripts.

We thank the reviewer for their contribution.

Reviewer #3 (Remarks to the Author):

The manuscript is improved after revisions. It presents interesting findings but still comes across as unfocussed, with limited mechanistic insights.

We thank the reviewer for acknowledging the improvements in the manuscript and hope that we have now sufficiently improved the focus and clarified the mechanisms.

1. Do UC-associated Tregs have reduced GDPD3? This would help draw a parallel with mouse models of perturbed epithelial NF-κB.

We very much appreciate this helpful suggestion. Indeed, Tregs in UC have reduced GDPD3 (Figure B). A similar question was raised by reviewer 1, who inquired about GDPD3 and CCR4.
We therefore share the response to both questions below:
We analysed publicly available scRNA-seq datasets from patients with ulcerative colitis (UC) and compared them with non-IBD controls (GSE116222). In our mouse model, we show that Gdpd3 is induced in Tregs under conditions of epithelial NF-κB activation. We therefore asked whether a similar induction occurs in UC. However, in contrast to the controlled NF-κB activation in our mouse model, UC is characterized by dysregulated rather than sustained epithelial NF-κB activation, as discussed in the manuscript. Consistent with this distinction, despite equivalent number of Tregs, GDPD3 expression was decreased in UC samples (Figure B). It should be noted, however, that GDPD3^{+} Tregs are relatively rare even in healthy samples, which limits the strength of conclusions that can be drawn from currently available datasets. A more definitive assessment of GDPD3 regulation in human disease would likely require a setting with well-defined epithelial NF-κB activation as a positive control, such as in acute infectious colitis, where epithelial NF-κB signalling is robustly induced.

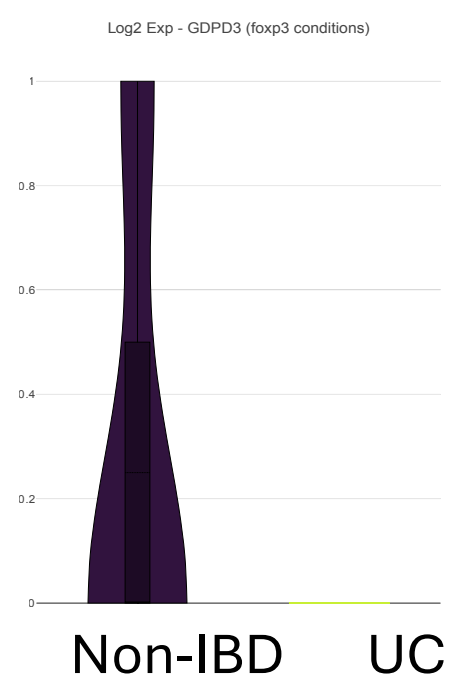

Figure B: Analysis of GDPD3 in FOXP3+ cells in non-IBD vs UC patient samples -- inflamed colon.
(from GSE116222).
Log2 Exp shown.

Staining of GDPD3 in UC versus non-IBD biopsies revealed a decrease in a number of GDPD3+ CD4+ cells in UC (Figure C), however as mentioned above, a positive control would be required to definitely determine the expected number.

With respect to CCR4, several studies have examined its expression in UC and reported reduced CCR4 expression in $CD4^+$ T cells, including $CD4^+CD25^+$ populations(*3*). Similarly, another study reported no increase in $CCR4^+$ cells in UC patient colon biopsies compared with healthy controls(*4*). In the scRNA-seq datasets available to us, CCR4 expression could not be robustly analysed due to the very low number of $CCR4^+$ cells detected (typically fewer than ~10 cells per dataset).
Taken together, these analyses suggest that the CCR4/Gdpd3 program observed in our mouse model of epithelial NF-κB activation is not recapitulated in UC, consistent with the notion that UC represents a state of dysregulated inflammation rather than controlled epithelial NF-κB activation.

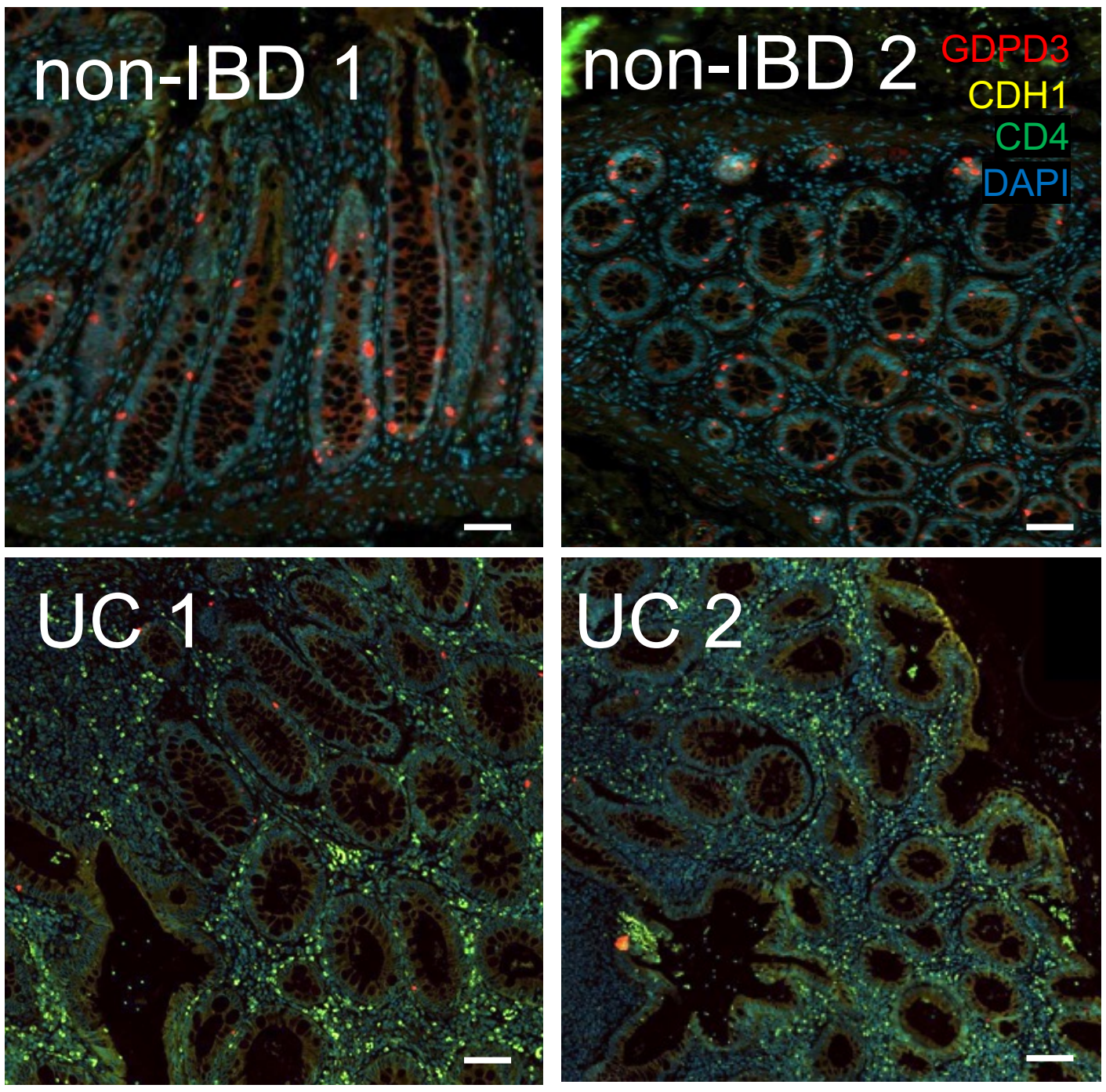

Figure C: IF of GDPD3 in UC (patient 1 and 2) versus "healthy" – non- IBD patient samples 1 and 2. Representative images shown. Scale bar: 50 μm.

2. Abstract "While the role of immune cells in colitis is well established" is an overstatement that has not been edited.

We have now rephrased this sentence in the abstract.

3. Cellular populations such as IE-T cells, PMNs and macrophages (Fig 4) should be presented as total #s. Percentages can be misleading as they can be due to a significant change in other cells, not just the numerator. For example, Fig 4A, there seems to be no change in neutrophils between WT Ut and Col groups.

We thank the reviewer for this important comment, which allowed us to better characterize the immune phenotype observed in the mice. We agree that reporting absolute cell numbers provides a more accurate representation of immune composition in this context.
In response, we have reanalysed these data and now also present the relevant immune populations as absolute cell numbers normalized to tissue weight (cells per gram tissue) in the revised manuscript (Supplemental Figure 2). This analysis confirms that the overall conclusions of the study remain unchanged, while providing a more precise representation of immune cell composition. Specifically, we observe a marked reduction in $TCR\beta^+$ T cells and $Ly6G^+$ neutrophils in *IκBα* $^{IEC\ KO}$ mice. Independent analysis of immune cell composition using scRNA-seq data (expressed as percentages of $CD45^+$ cells) supports these findings: both T cells and neutrophils are reduced in *IκBα* $^{IEC\ KO}$ mice, whereas Tregs show a modest increase (new Extended Data 4j).
Importantly, our previous analyses estimated Tregs as $FoxP3^+$ cells within the $CD4^+$ T cell compartment. Because the total number of $CD4^+$ T cells is reduced in *IκBα* $^{IEC\ KO}$ mice,

expressing Tregs as a percentage of CD4$^+$ cells introduced a denominator effect. The revised analysis therefore clarifies the magnitude of the change while leaving the overall biological interpretation unchanged.
With respect to the reviewer's specific example, we agree that in the original percentage-based presentation of Fig. 4A, neutrophils appeared not to differ substantially between WT untreated and WT colitis. However, when analysed as absolute cell numbers, the differences become clearer and are no longer influenced by changes in relative population frequencies (Supplemental Figure 2).
Notably, previous studies have shown that exogenous administration of Tregs can lead to reductions in neutrophils and conventional T cells(*5*). Our findings are consistent with this model, as the presence of activated Tregs in *IκBα* $^{IEC\ KO}$ mice is associated with a reduction in Ly6G$^+$ neutrophils and TCRβ$^+$ T lymphocytes, suggesting that Treg-mediated immune regulation contributes to the remodelling of the intestinal immune compartment.
Overall, these revised analyses further reinforce our conclusion that epithelial NF-κB activation leads to a broader remodelling of the colonic immune landscape, rather than isolated changes in individual immune populations. We have updated the Results to clarify this point.

4. While of interest, the comparison between normal and UC human biopsies seems irrelevant to the rest of the story

We thank the reviewer for this comment. The comparison between normal and UC human biopsies was included to provide translational context for the epithelial–immune mechanisms identified in our experimental models. Specifically, these analyses illustrate that NF-κB signalling in UC is not uniformly activated within intestinal epithelial cells, but rather appears dysregulated and heterogeneous. As a result, the coordinated epithelial NF-κB–driven programs observed in our mouse model may not operate in the same way in human UC. We therefore included the human data primarily to highlight this distinction and to place our mechanistic findings in a broader clinical context. To avoid potential confusion regarding the scope of these analyses, we have clarified this point in the revised manuscript and now more explicitly state that the human biopsy comparison serves as contextual support rather than a central mechanistic component of the study.

Reviewer #4 (Remarks to the Author):

The revision has improved the clarity and highlighted the strengths of the manuscript, I have no further comments.

We appreciate the work and the helpful suggestion the reviewer has provided and are happy that the reviewer is satisfied with the revision.

Bibliography

1. S. Galvan-Pena, Y. Zhu, B. S. Hanna, D. Mathis, C. Benoist, A dynamic atlas of immunocyte migration from the gut. *Sci Immunol* **9**, eadi0672 (2024).
2. M. Iwata *et al.*, Retinoic acid imprints gut-homing specificity on T cells. *Immunity* **21**, 527-538 (2004).
3. Y. Jo *et al.*, CCR4 is an up-regulated chemokine receptor of peripheral blood memory CD4+ T cells in Crohn's disease. *Clin Exp Immunol* **132**, 332-338 (2003).
4. M. Oki *et al.*, Accumulation of CCR5+ T cells around RANTES+ granulomas in Crohn's disease: a pivotal site of Th1-shifted immune response? *Lab Invest* **85**, 137-145 (2005).
5. B. Nayer *et al.*, Local administration of regulatory T cells promotes tissue healing. *Nat Commun* **15**, 7863 (2024).

REVIEWER COMMENTS

Reviewer #1 (Remarks to the Author):

Although the authors have made efforts to revise the manuscript, several of the major concerns raised in the previous round of review remain inadequately addressed. As a result, the current data are still insufficient to fully support the main conclusions of the study.

1. While the manuscript emphasizes the importance of niche-specific reprogramming, it does not sufficiently account for the observed systemic changes in Treg function. The authors propose several possible mechanisms, including humoral factor–mediated effects, indirect modulation via antigen-presenting cells (APCs), and Treg recirculation. However, these remain speculative and are not experimentally validated in the present study. Such mechanisms could be addressed through approaches including parabiosis, serum transfer, ex vivo antigen presentation assays using APCs, or trafficking analyses.

We thank the reviewer for this important comment and for suggesting additional experiments. In response, we have: (i) revised the title, Abstract, Results and Discussion to distinguish conclusions supported by *in vivo* evidence from associations supported only by in vitro experiments; (ii) expanded our *in vivo* immunophenotyping to include additional animals and Treg markers; and (iii) performed the suggested APC co-culture experiments to examine whether CD11c+ cells contribute to the observed Treg phenotype. These changes are highlighted in blue in the revised manuscript.
Our revised model is that the epithelial NF-κB–dependent secretome promotes Treg recruitment and is associated with the accumulation of Tregs with enhanced suppressive capacity, thereby contributing to protection from colitis. The following *in vivo* findings support this model:
a. Epithelial NF-κB–dependent secretome:
Using complementary mouse models permitting IEC-specific NF-κB activation, suppression or visualisation, we characterized epithelial NF-κB activity and its downstream secretome by scRNA-seq, epithelial RNA sequencing, multiplex immunofluorescence and secretome analysis of freshly isolated intestinal crypts.
b. Treg recruitment: *In vivo* immunophenotyping (new Figure 4, Extended data 4, Supplemental Figure 1 and 2) and scRNA-seq demonstrated increased accumulation of colonic Tregs in the *IκBα* $^{IEC\ KO}$ mice. CCL22 KO mice are sensitised to colitis and do show compromised Treg recruitment to the gut[1]. Although this phenotype was originally attributed solely to CD11c+ dendritic cells, we now showed that IEC in *IκBα* $^{IEC\ KO}$ mice also produce CCL22. Consistent with an epithelial contribution to Treg recruitment, conditioned medium from NF-κB–activated organoids promoted Treg migration, whereas epithelial CCL22 deficiency abolished this effect. Together, these findings support a role for epithelial NF-κB–dependent CCL22 in Treg recruitment, although we do not claim to have demonstrated the complete recruitment mechanism *in vivo*.
c. Accumulation of Tregs with enhanced suppressive capacity:
Tregs isolated from *IκBα* $^{IEC\ KO}$ mice showed increased suppressive activity in an *in vivo* adoptive-transfer experiment. Additional immunophenotyping demonstrated an enhanced activation-associated phenotype, while freshly isolated, non-expanded Tregs from these mice produced markedly more IL-10. These experiments establish an association between epithelial NF-κB activation and the accumulation of functionally enhanced Tregs. They do not, however, establish that epithelial NF-κB actively reprograms Tregs *in vivo*. We have therefore removed or qualified statements referring to Treg reprogramming throughout the manuscript.

d. Protection from colitis:
The protective effect of epithelial NF-κB was demonstrated using reciprocal genetic models. IEC-specific NF-κB suppression in ΔN^{IEC} mice increased susceptibility to DSS-induced colitis, whereas constitutive IEC NF-κB activation in $I\kappa B\alpha^{IEC\ KO}$ mice conferred protection, as assessed by clinical parameters, histology, multiplex immunofluorescence and scRNA-seq.

As the reviewer 1 pointed out, our study shows that changes in the Treg phenotype are in part systemic. To investigate whether these systemic observations could be related to gut-associated trafficking, we expanded the immunophenotyping analysis to six mice per group and examined CCR9 expression on splenic CD4+FoxP3+ Tregs (New Extended data 4). CCR9 is associated with intestinal homing, and its expression on splenic Tregs is consistent with a relationship between the systemic population and the intestinal immune response. However, this analysis does not establish the direction of trafficking or determine whether the cells acquire their phenotype within the intestine before recirculating.
To address the potential contribution of APCs experimentally, as suggested by the reviewer, we co-cultured primary Tregs with CD11c+ cells in the presence or absence of conditioned medium from WT or $I\kappa B\alpha^{IEC\ KO}$ mouse organoids (New Extended data 6g-i). Because prolonged CD3/CD28-driven expansion can bias Treg phenotype towards classical lymphoid state and diminish tissue-associated Treg phenotypes[2, 3], these experiments were performed without direct CD3/CD28 stimulation.
Co-culture with CD11c+ cells increased Treg proliferation and the expression of CCR4 and ICOS. However, conditioned medium from either WT or $I\kappa B\alpha^{IEC\ KO}$ organoids did not independently promote proliferation or further enhance CD11c+-dependent expansion (New Extended data 6g and h). Thus, although CD11c+ cells can support Treg proliferation and activation *in vitro*, this effect did not account for the genotype-specific Treg phenotype.
We next measured IL-10 to distinguish effects on proliferation from effects on suppressive function. IL-10 was undetectable in the organoid-conditioned media before their addition to the cultures. CD11c+ cells did not increase IL-10 production, whereas conditioned medium from $I\kappa B\alpha^{IEC\ KO}$ organoids induced substantially greater IL-10 production by primary Tregs than conditioned medium from WT organoids (New Extended data 6i). These findings indicate that, under our *in vitro* conditions, factor or factors within the epithelial NF-κB–dependent secretome can act directly on Tregs to enhance IL-10 production without requiring CD11c+ cells.
The responsible epithelial factor or combination of factors remains to be identified. The secretome analysis identified several plausible candidates, including IL-33, which can promote Treg expansion and activation through ST2. We now present this only as a potential mechanism. Establishing its contribution would require dedicated *in vivo* genetic experiments and is beyond the scope of the present study.
We agree that parabiosis, serum-transfer and dedicated trafficking or fate-mapping experiments would provide valuable information about the relationship between intestinal and systemic Treg populations. However, these would represent substantial new *in vivo* studies. The precise epithelial factor or combination of factors responsible for establishing this phenotype, and the extent to which intestinal Tregs subsequently recirculate to systemic compartments, remain important questions for future investigation.

2. The observed reduction of Gdpd3 expression in Tregs from UC patients is potentially an important finding and should be presented in the figures. In addition, the quality of the

immunostaining requires improvement, as the co-localization of CD4 and Gdpd3 is not clearly demonstrated.

We thank the reviewer for this comment. We replaced the immunofluorescence images and added magnified regions to more clearly identify CD4+GDPD3+ cells. We also adjusted the channel colours to improve visualization of CD4 and GDPD3 co-localization, which appears yellow in the merged images (new Fig. 6k).
We agree that the relationship between GDPD3 expression in Tregs and human disease warrants further investigation. We therefore extended our analysis to publicly available scRNA-seq datasets comprising patients with UC and healthy controls (GSE157477, GSE125527, GSE231993 and GSE166555). *GDPD3*+ Tregs were extremely rare in both healthy and IBD samples, with numerically lower frequencies in the disease groups. Given the very small number of *GDPD3*+ cells detected, these datasets do not permit a robust assessment of whether *GDPD3*+ Tregs are reduced in UC. Larger datasets containing substantially greater numbers of Tregs, particularly from patients with UC, will be required to resolve this question (Figure for Reviewer A).
Because CRC is associated with high epithelial NF-κB activity[4], we additionally analysed the publicly available scRNA-seq datasets GSE166555 and GSE178341. In contrast to the IBD datasets, *GDPD3*+ Tregs were significantly enriched in colorectal tumours compared with healthy colorectal tissue (Figure for Reviewer A, lower panels). We next asked whether *GDPD3*+ Tregs were enriched for the markers identified in our mouse models or for genes associated with enhanced suppressive function. However, the small number of *GDPD3*+ Tregs limited the statistical power of this differential-expression analysis, and no significant differences were detected.

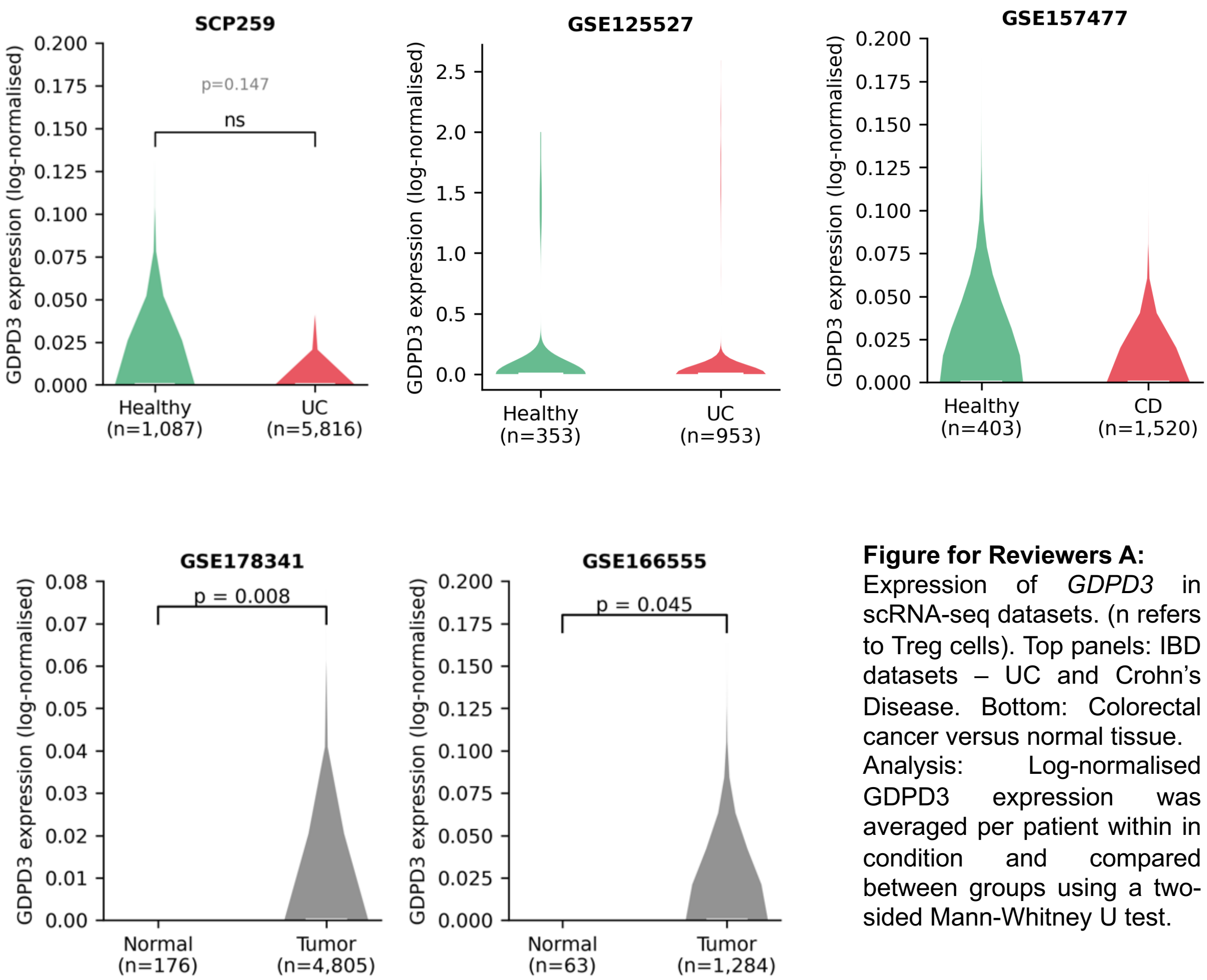

Figure for Reviewers A: Expression of *GDPD3* in scRNA-seq datasets. (n refers to Treg cells). Top panels: IBD datasets – UC and Crohn's Disease. Bottom: Colorectal cancer versus normal tissue. Analysis: Log-normalised GDPD3 expression was averaged per patient within in condition and compared between groups using a two-sided Mann-Whitney U test.

Although detailed functional and transcriptional characterization of GDPD3+ Tregs in UC and CRC is beyond the scope of the present study, these observations identify their relationship with human intestinal disease as an important direction for future investigation. We thank the reviewer for prompting this additional analysis.

3. The presentation and interpretation of cell numbers and statistical analyses require greater rigor and clarity. It appears that the p-value on line 281 may be incorrect. A sample size of n = 3 appears insufficient. Moreover, terms such as "decreased" should not be used in the absence of statistical significance.

We agree with the reviewer that a sample size of n = 3 was insufficient for robust immunophenotyping analysis. We therefore repeated the experiment using six mice per group, thereby increasing the statistical power of the analysis. We also expanded the panel to include ICOS, CCR4 and CCR9 (new Fig. 4, Extended Data Fig. 4 and Supplementary Figs. 1–2). In addition to presenting absolute cell numbers in the main figures, we now report the corresponding frequencies among CD45+ cells in Supplementary Fig. 2. The p value reported on line 281 was removed.
Finally, we revised the text throughout to ensure that terms such as "increased" or "decreased" are used only for statistically significant differences; non-significant trends are described as such.

Reviewer #2 (Remarks to the Author):

I co-reviewed this manuscript with one of the reviewers who provided the listed reports. This is part of the Nature Communications initiative to facilitate training in peer review and to provide appropriate recognition for Early Career Researchers who co-review manuscripts.

We thank the reviewer for continues help and feedback.

Reviewer #3 (Remarks to the Author):

Authors have provided additional data in response to previous comments. They have also better acknowledged limitations of their experiments and associated analysis.

However, as also stated by R1, the central claim that epithelial NF-kB promotes Treg recruitment and differentiation is still largely unsupported by the data:

1. There is no discernible difference in overall #s of Tregs in IEC-IkBa KO colons by FACS, and sample size in these experiments is quite small.

We thank the reviewer for this comment and agree that the original sample size was too small to draw robust conclusions regarding Treg numbers. We therefore repeated the immunophenotyping analysis in an independent cohort with six mice per experimental group. In this expanded analysis, the total number of colonic CD4+FoxP3+CD25+ Tregs was significantly increased in $I\kappa B\alpha^{IEC\ KO}$ mice compared with WT controls, consistent with our previous immunofluorescence analysis of the distal colon. These new data are presented in new Fig. 4, Extended Data Fig. 4 and Supplementary Figs. 1–2.

2. There is no in vivo support for the claim that epithelial NF-kB induced Ccl22, or any other factor, drives increased Treg infiltration or differentiation.

We agree that the original wording did not sufficiently distinguish between induction of epithelial CCL22, Treg recruitment and Treg differentiation. We have therefore revised the manuscript throughout to clarify the level of evidence supporting each conclusion. Our *in vivo* data demonstrate that epithelial NF-κB activation is associated with increased epithelial CCL22 production and increased accumulation of colonic Tregs. Specifically, epithelial CCL22 induction was detected by scRNA-seq, immunofluorescence and secretome analysis of freshly isolated intestinal crypts from *IκBα* $^{IEC\ KO}$ mice. However, we have not genetically isolated the epithelial contribution of CCL22 *in vivo* and therefore do not claim that epithelial-derived CCL22 is the sole factor responsible for the increased Treg accumulation.
The functional contribution of epithelial CCL22 is supported by our organoid migration experiments. Conditioned medium from NF-κB–activated organoids promoted Treg migration, whereas loss of epithelial CCL22 markedly impaired this effect. These findings demonstrate that epithelial-derived CCL22 is one functional component of the NF-κB–dependent secretory program capable of promoting Treg recruitment.
Previous studies showing increased susceptibility of global CCL22-deficient mice to DSS colitis provide additional *in vivo* evidence for a protective role of the CCL22 axis, although they do not distinguish epithelial from hematopoietic sources[1].
Finally, we agree that our data do not demonstrate Treg differentiation *in vivo*. We have therefore removed or moderated statements referring to Treg differentiation or reprogramming and instead describe the accumulation of Tregs with enhanced suppressive capacity.

3. Functional analysis rely on MLN-derived Tregs

We agree with the reviewer and clearly state this limitation in the discussion.
Although the functional assays were performed with MLN-derived Tregs, our analysis of Tregs from spleen and colon via scRNA, phenotyping, and ELISA, demonstrated a broad transcriptional alteration in Tregs from *IκBα* $^{IEC\ KO}$ mice, including enrichment of genes associated with gut homing and suppressive function. This supports the interpretation that the enhanced phenotype is not restricted to a rare Treg subset. However, we do not infer that every individual Treg is equivalently reprogrammed.
MLNs were selected because they yielded sufficient cell numbers for functional analysis and constitute the principal lymphoid compartment draining the intestine. We interpret these findings together with the increased accumulation and activated phenotype of Tregs observed in the colon *in vivo* and have clarified this distinction in the revised manuscript.

In my opinion, manuscript can be accepted if authors limit the conclusion to epithelial NFkB signaling being protective in colitis, and this protection being associated with Tregs, and leave the exact mechanisms of how epithelial NFkB protect from colitis as yet-undetermined.

We appreciate the reviewer's suggestion and agree that the precise molecular and cellular pathways through which epithelial NF-κB signaling protects against colitis are not yet fully resolved. We have therefore revised the title, Abstract, Results and Discussion to distinguish conclusions supported directly by *in vivo* evidence from mechanisms supported by complementary *ex vivo* and *in vitro* experiments. Our revised conclusion is that epithelial NF-κB signaling protects against colitis and is associated with the accumulation of Tregs with enhanced suppressive capacity. In addition, our data

implicate epithelial-derived CCL22 as one component of the NF-κB–dependent secretory program capable of promoting Treg recruitment. We do not claim that CCL22 is the sole mediator of Treg recruitment and acknowledge that the full combination of mediators and cellular interactions underlying this protection remains to be defined.

1. Rapp, M. *et al.* CCL22 controls immunity by promoting regulatory T cell communication with dendritic cells in lymph nodes. *J Exp Med* **216**, 1170-1181 (2019).

2. Arpaia, N. *et al.* A Distinct Function of Regulatory T Cells in Tissue Protection. *Cell* **162**, 1078-1089 (2015).

3. Mensink, M. *et al.* Tregs from human blood differentiate into nonlymphoid tissue-resident effector cells upon TNFR2 costimulation. *JCI Insight* **9** (2024).

4. Vaiopoulos, A.G., Athanasoula, K. & Papavassiliou, A.G. NF-kappaB in colorectal cancer. *J Mol Med (Berl)* **91**, 1029-1037 (2013).

REVIEWERS' COMMENTS

Reviewer #1 (Remarks to the Author):

The new Extended data provide meaningful functional support for the authors' central model. In particular, conditioned medium from NF-κB-activated epithelial organoids enhanced IL-10 production by primary Tregs independently of CD11c+ cells, supporting a direct effect of one or more epithelial secreted factors on Treg function.

However, the molecular basis of this effect remains unresolved. Although the secretome analysis identified plausible candidates, including IL-33, the responsible factor or combination of factors was not experimentally defined. Thus, the revised study establishes a functional epithelial secretome–Treg link in vitro, but does not yet provide a molecular mechanism for this interaction or demonstrate its causal contribution in vivo.

The human GDPD3 analysis was less informative. GDPD3+ Tregs were too rare in the UC datasets to permit a robust conclusion, and the enrichment observed in colorectal cancer does not directly establish their relevance to UC. This reduces the translational impact of this aspect of the study, although it does not invalidate the principal findings.

We thank the reviewer for this thoughtful assessment and for recognizing that the new data provide meaningful functional support for our central model. We agree that the specific epithelial factor(s) mediating the direct effect on Tregs remain to be identified, and that the current GDPD3 analysis does not establish its relevance to UC. We appreciate the reviewer's recognition that these limitations do not affect the principal conclusions of the study.

Reviewer #2 (Remarks to the Author):

I co-reviewed this manuscript with one of the reviewers who provided the listed reports. This is part of the Nature Communications initiative to facilitate training in peer review and to provide appropriate recognition for Early Career Researchers who co-review manuscripts.

We thank the reviewer for their contribution.

Reviewer #3 (Remarks to the Author):

Authors have provided new data or modified their conclusions to sufficiently assuage my concerns.

We thank the reviewer for their contribution and help.
