# Supplementary material for: Epithelial NF-κB shapes protective Treg cell responses to restrain intestinal inflammation
Source: Nat Commun. 2026 Oct 20;17:10956. doi: 10.1038/s41467-026-78796-z (PMC13683377; doi:10.1038/s41467-026-78796-z)
Supplement: Supplementary file 3 [file 41467_2026_78796_MOESM1_ESM.pdf]

Figure S1: Activated NF-κB in homeostasis, colitis, and recovery

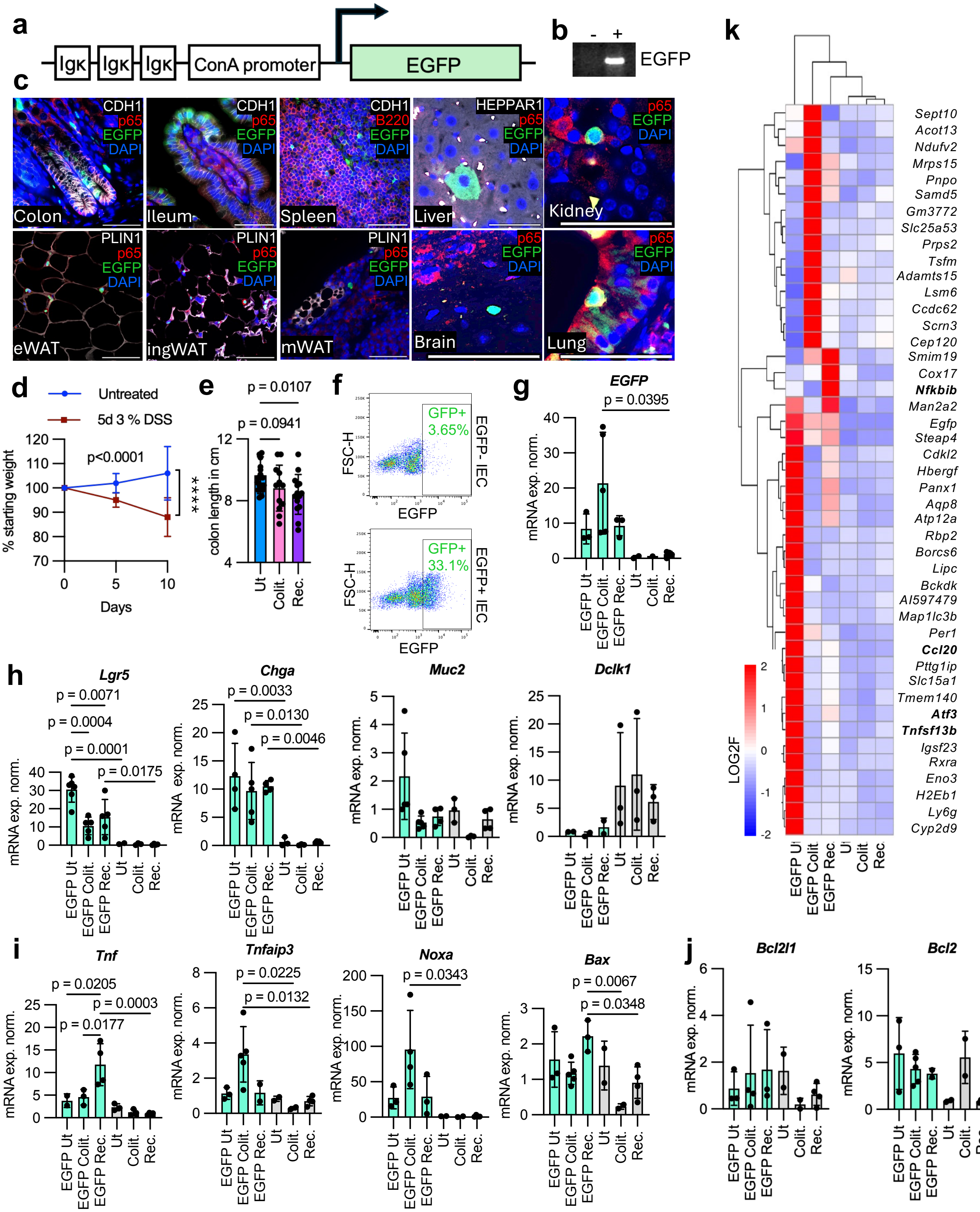

Supplementary Figure 1: a. Reporter construct for NF-κB activity with three Ikappa binding sites fused to the EGFP sequence – previously described in *(20)*. **b.** Presence of *EGFP* detectable by PCR in mouse biopsies. **c.** Representative immunofluorescence, showing presence and localisation of activated NF-κB versus presence of the dominant subunit p65. Tissue specific markers are shown. Ing, e, mWAT: inguinal, epididymal, mesenteric White Adipose Tissue. Scale: 50µm. **d.** Weight of untreated mice or mice on 3% DSS treatment (day 1-5) and recovery (day 6-10). n = 10. $p<0.0001$. **e.** Colon of mice as in d. Analysis: One-way ANOVA with Tukey's correction. **f.** FACS isolation of EGFP+ epithelial cells. Gating shown. **g.** RNA from EGFP+ epithelial cells analysed by qPCR. EGFP sorted IEC in green. Unsorted IEC in grey. Analysis: One-way ANOVA with Tukey's correction. **h.** As in g. with cell type specific markers. p value shown only when significant. **i.** As in g. with pro-inflammatory targets of NF-κB. **j.** as in g. with anti-apoptotic targets of NF-κB. **k.** scRNA heatmap of significantly enriched genes in EGFP+ versus negative IEC. Previously validated NF-κB target genes shown in bold.

Figure S2: Suppression of IEC NF-κB prevents recovery

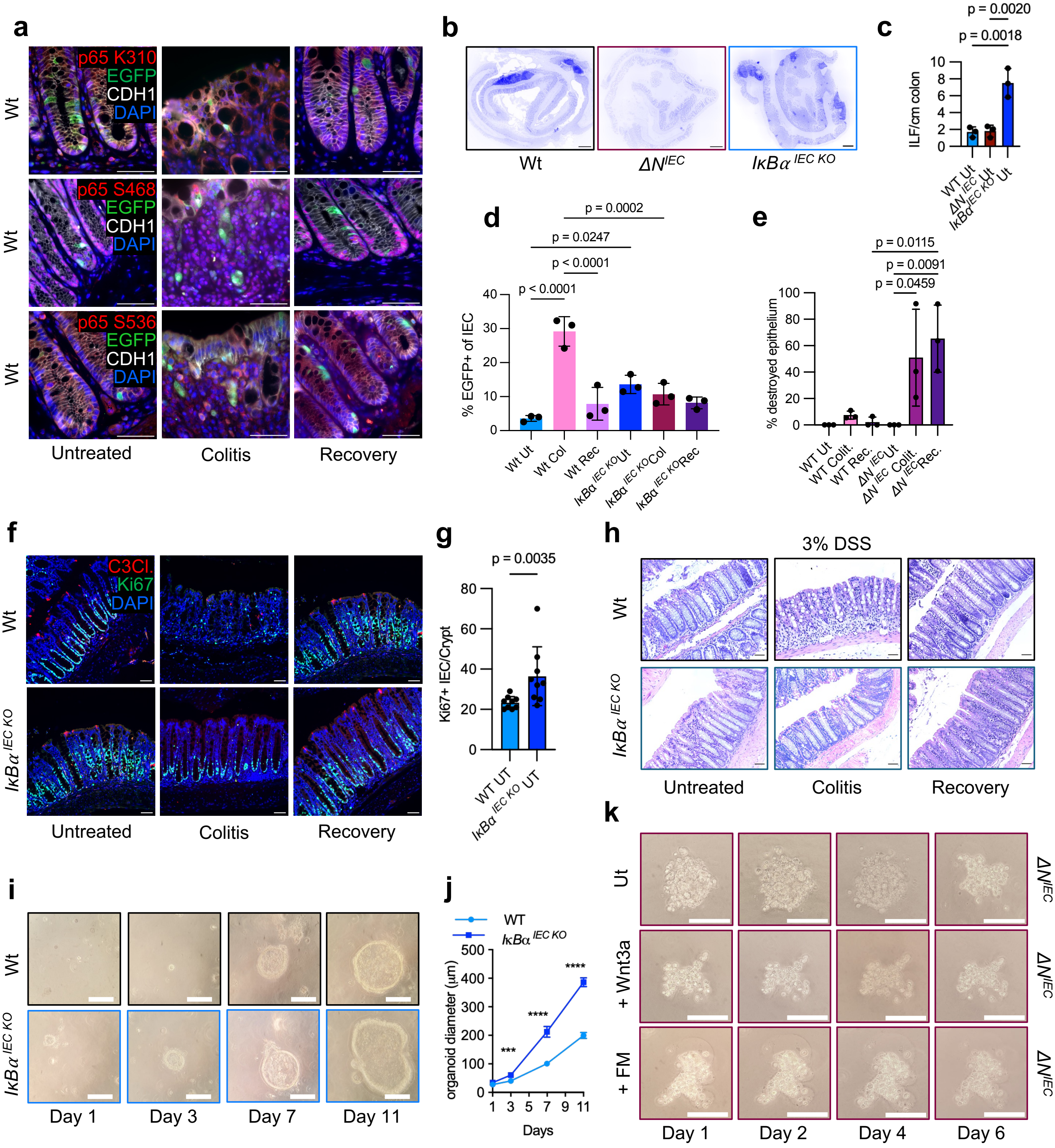

Supplementary Figure 2: a. NF-κB activation (EGFP+ cells) versus post-translational modifications previously used as bona-fide markers of activity, including K310, S468, and S536. from at least 5 mice. **b.** Isolated lymphoid follicles (ILF) stained with DAPI. Scale: 50 µm. **c.** Quantitation from b. of ILF per cm of colon. n = 3. Analysis: one-way ANOVA with Tukey's correction. **d.** EGFP+ CDH1+ cells were counted from at least 5 colonic sections per mouse (n = 3 mice per group). Analysis as in c. **e**. Entire colon was stained by H&E and percentage of destroyed epithelium (regions with colitis score > 3) quantitated. Analysis as in c. **f.** IF as in Fig. 2g with 3% DSS and mice with activated NF-κB. **g.** Quantitation of Ki67+ cells in untreated mice. Analysis: Student *t*-test. **h**. H&E conditions as in f. **i.** Colon derived organoids from Wt mice or mice with activated NF-κB. At least n = 6 mice per group, pooled. **j.** Quantitation of growth from i. (mean and SD). n = 6 mice per group. **k.** Crypts derived from Wt mice or those with suppressed IEC NF-κB (*ΔN^{IEC}*) were cultured in standard medium (top row), or with Wnt3a or with FM (full medium from primary autologous fibroblasts). n = 6. Scale: 50 µm.

Figure S3: NF-κB balances expression of genes mediating innate vs adaptive immune cell recruitment

a

Group	Treatment
Wt Ut	-
Wt Colitis	5d 1.5% DSS
Wt Recovery	5d 1.5% DSS & 5d water
ΔN^{IEC} Ut	-
ΔN^{IEC} Colitis	5d 1.5% DSS
ΔN^{IEC} Recovery	5d 1.5% DSS & 5d water

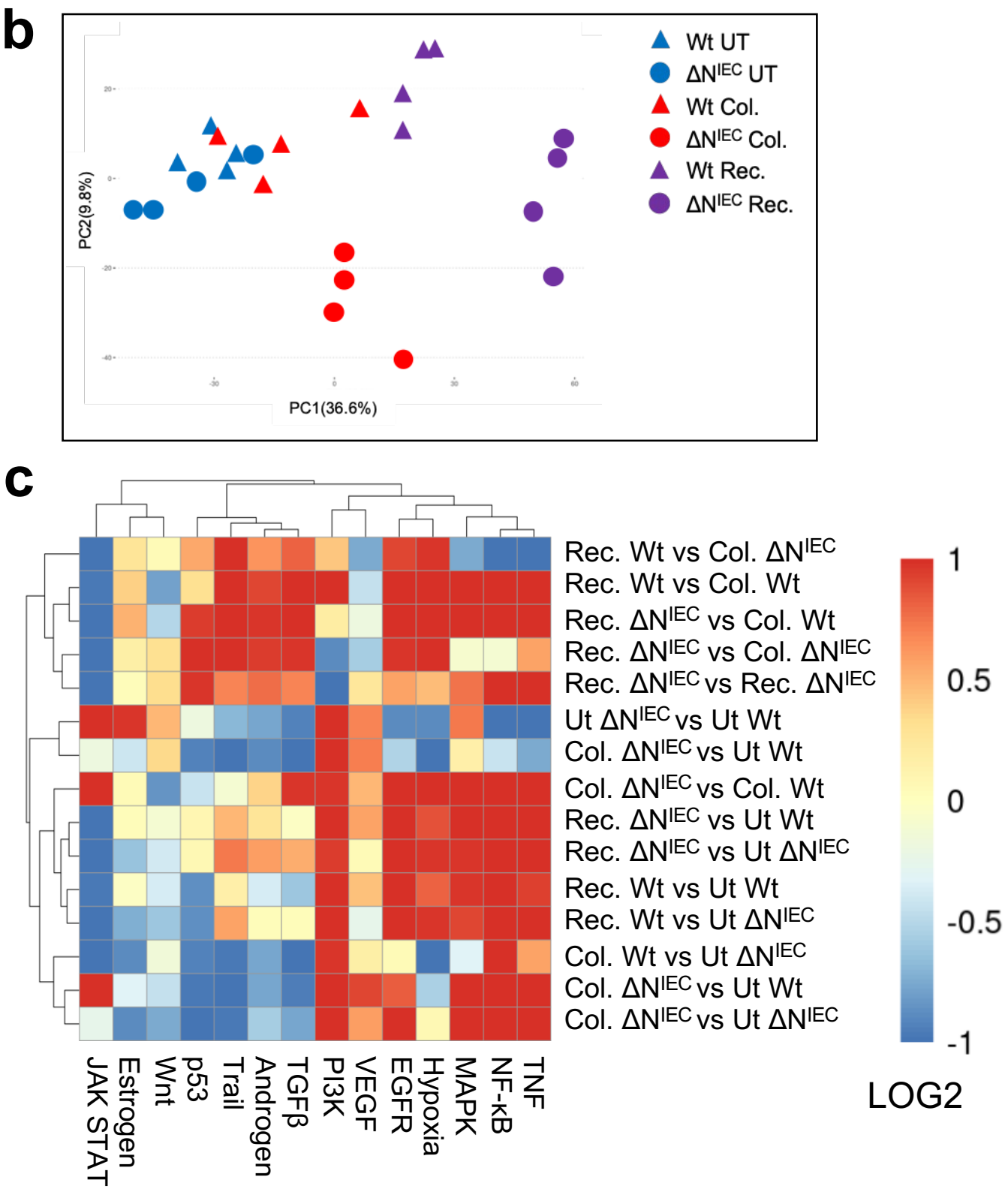

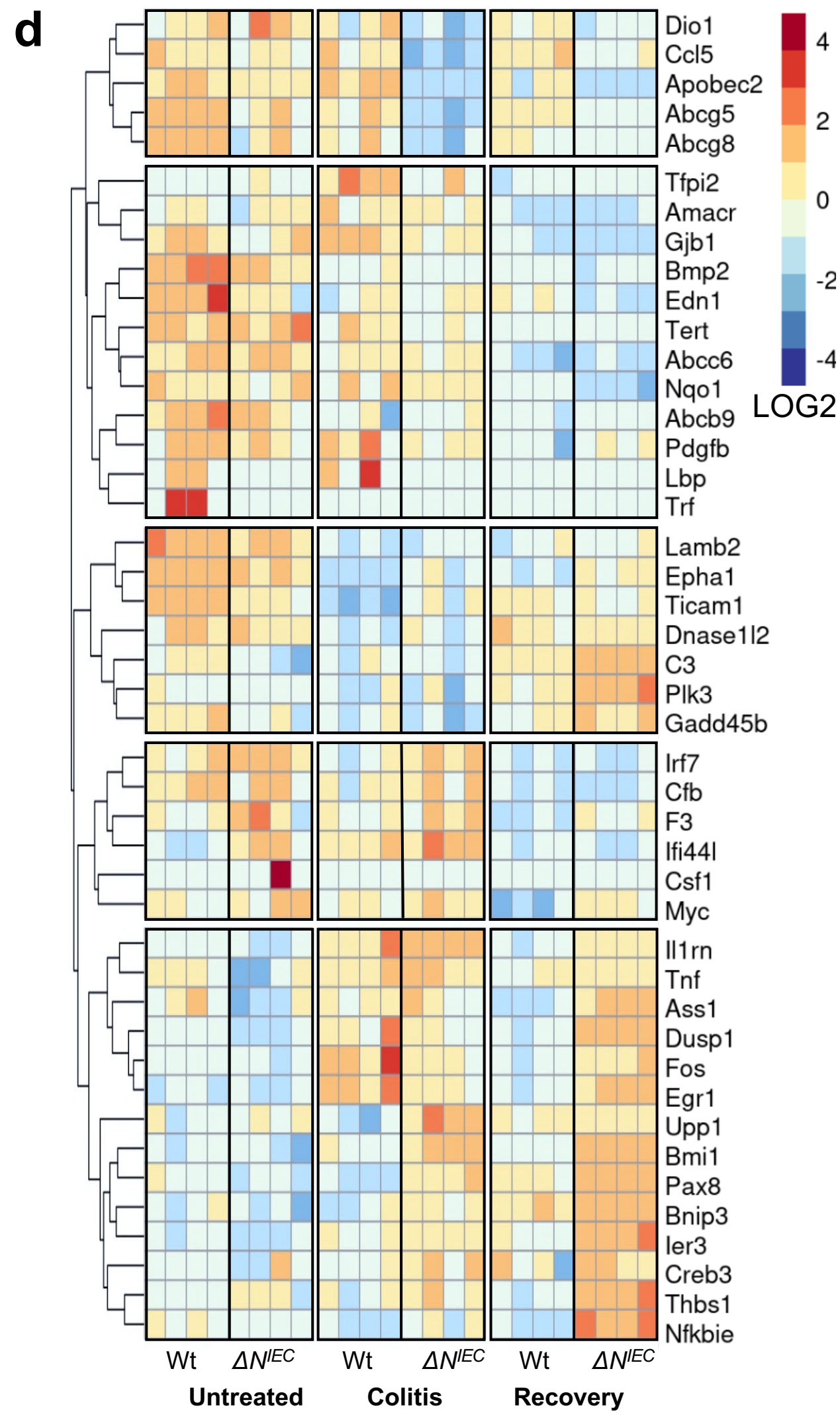

Supplementary Figure 3: a. Experimental groups and treatments. **b.** PCA plot of samples based on RNA-seq counts normalised using RUVSeq R package (used RUVs function with k = 10) **c.** Pathway perturbation signature scores computed using PROGENy R package based on the differential expression significance statistics (from DESeq2) of the compared pairs of sample groups. LOG2 Fold shown. **d.** Visualisation of known NF-κB target genes (from RNAseq in groups as in a.) which were found expressed in at least 1% of the epithelial cells and NOT expressed in more than 5% of the non-epithelial cells based on the scRNA data.

Figure S4: Epithelial secretome shapes mucosal immune compartment

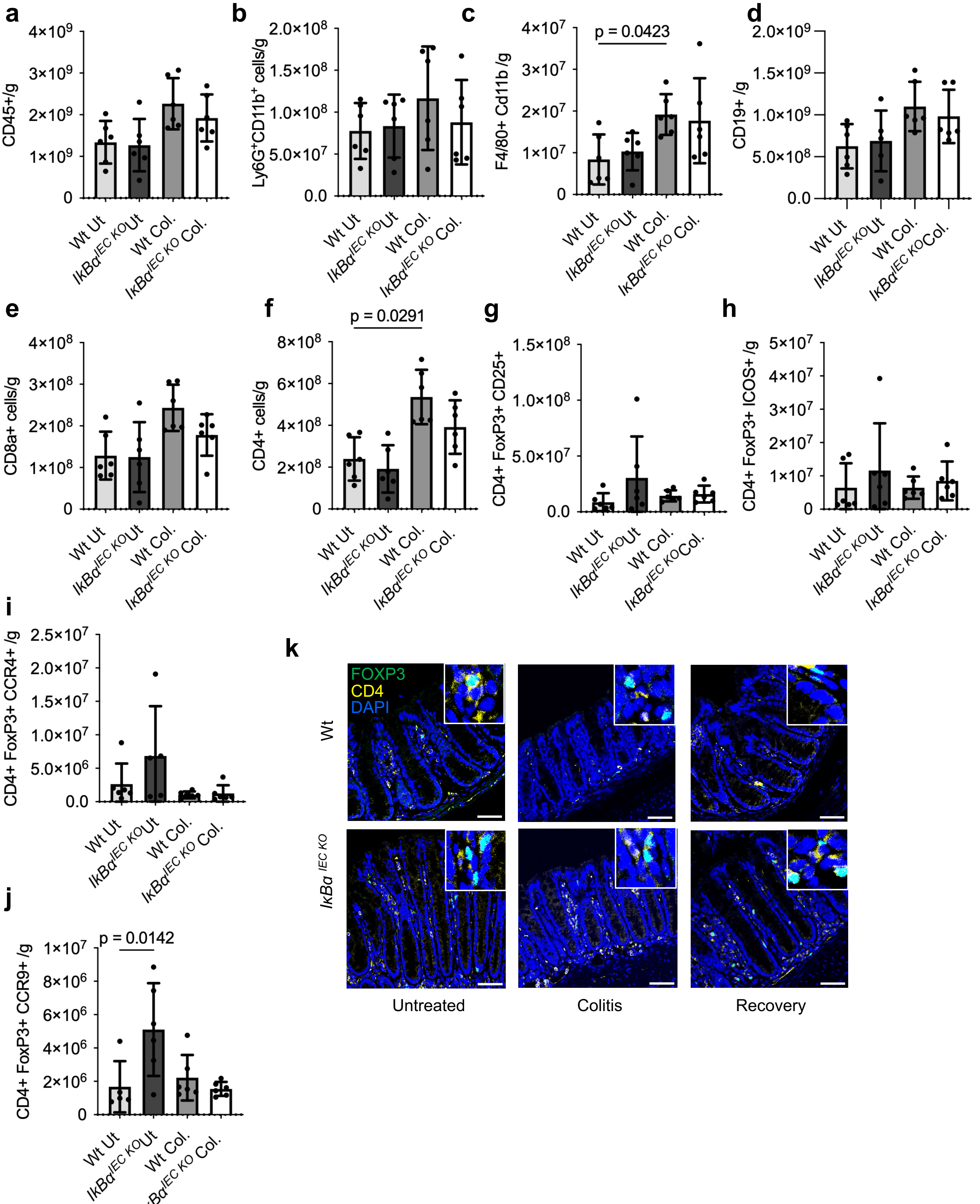

Supplementary Figure 4: a.-j. Quantification of immune phenotyping by flow cytometry on leucocytes from isolated splenic cells from $I\kappa B\alpha^{IEC\ KO}$ and Wt mice (n = 6 per group). Analysis as in Fig 4a-j. **k.** Representative stainings of Treg cells with FoxP3 and CD4. Zoom shows representative Treg cells. Scale: 50 µm.

Figure S5: Gating strategy for flow cytometry

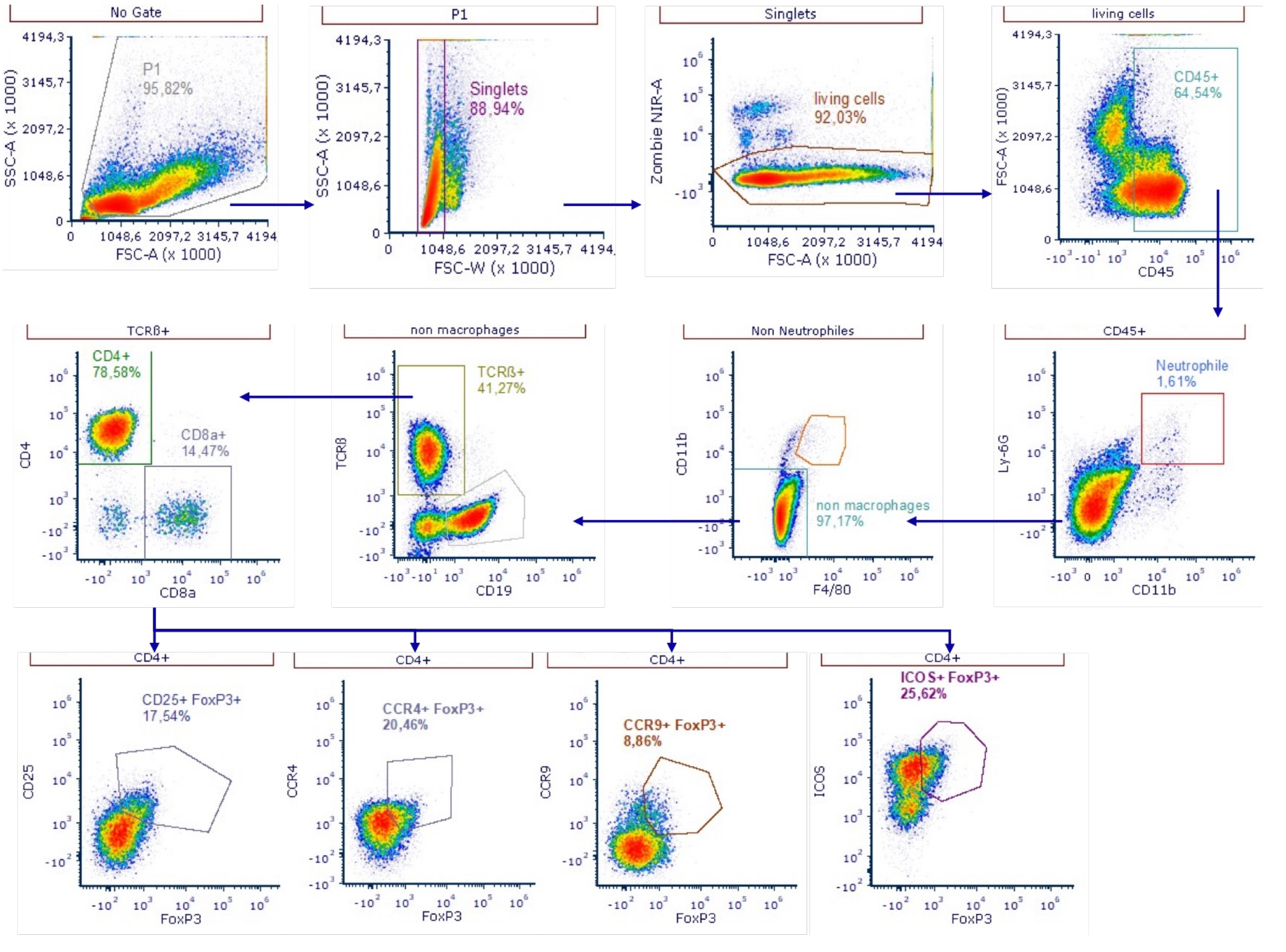

Supplementary Figure 5: Gating strategy for immunophenotyping (Figure 4).

Figure S6: Epithelial NF-κB shapes Treg phenotype that limits inflammation

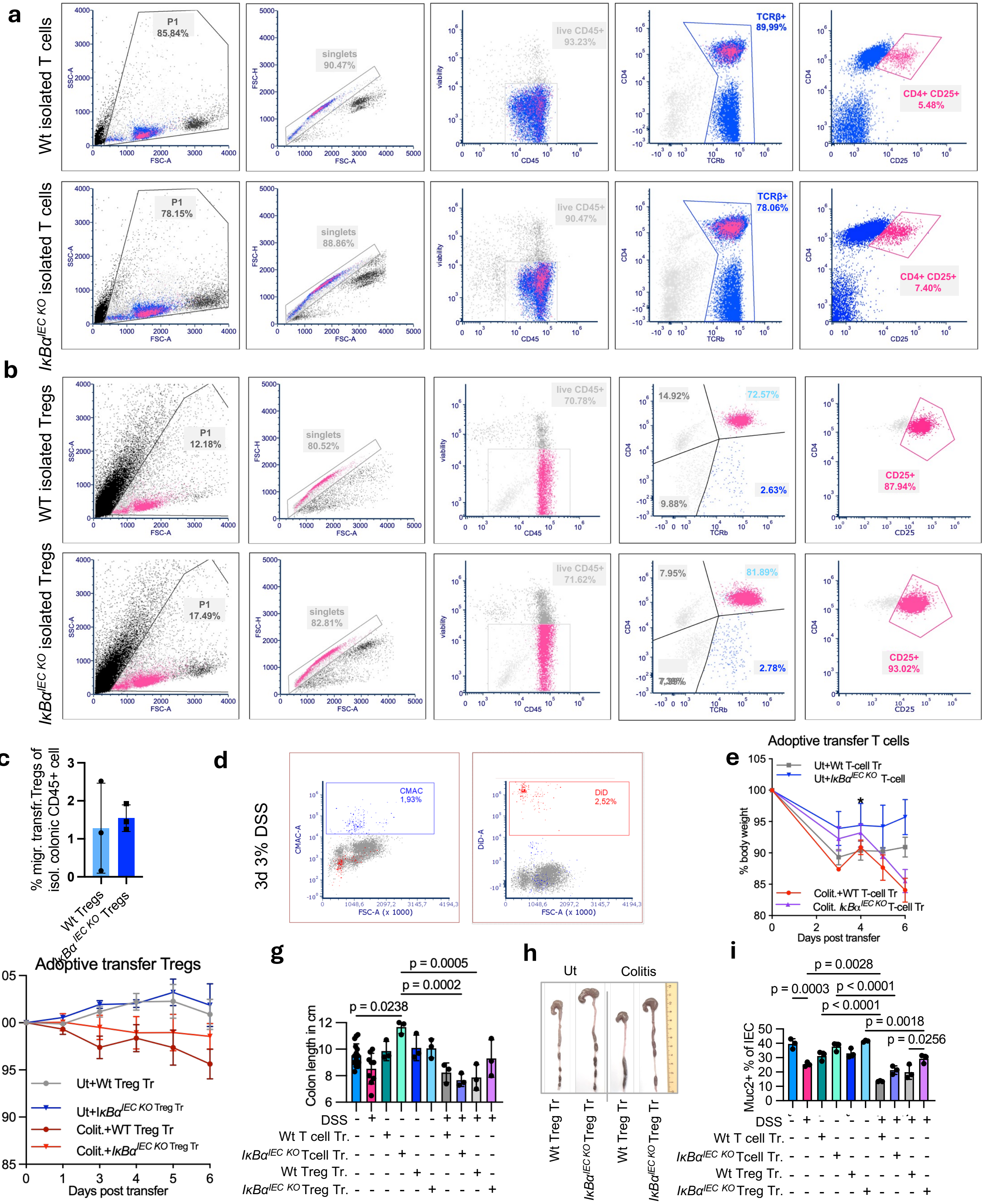

Supplementary Figure 6: a. and b. Gating for isolation of T lymphocytes and Treg cells. **a.** CD25+ cells analysed from CD4+ TCRβ+ quadrant. **c.** Isolated Treg cells from Wt or *IκBα$^{IEC\ KO}$* mice adoptively transferred into n = 3 Wt mice, plus 1 control (NaCl). All received 3%DSS. *IκBα$^{IEC\ KO}$* Treg cells stained with DiD and Wt Treg cells stained with CMAC. Quanti by flow cytometry. Analysis: Paired *t* test. **d.** Representative flow cytometry plots of migrated transferred Treg cells as percentage of isolated colonic lamina propria immune cells. **e.** % body weight after adoptive T cell transfer. Micetreated with DSS (Colit.) or untreated. Donor genotype indicated. **f.** as in c. with Treg cells transfer. **g.** Colon length from mice treated as in c and d. Analysis: one-way ANOVA with Tukey's correction. **h.** as in e. representative images. **i.** Quantitation of goblet cells (Muc2+), quantitation as in 2g.

Figure S7: Epithelial secretome promotes metabolic rewiring of Tregs

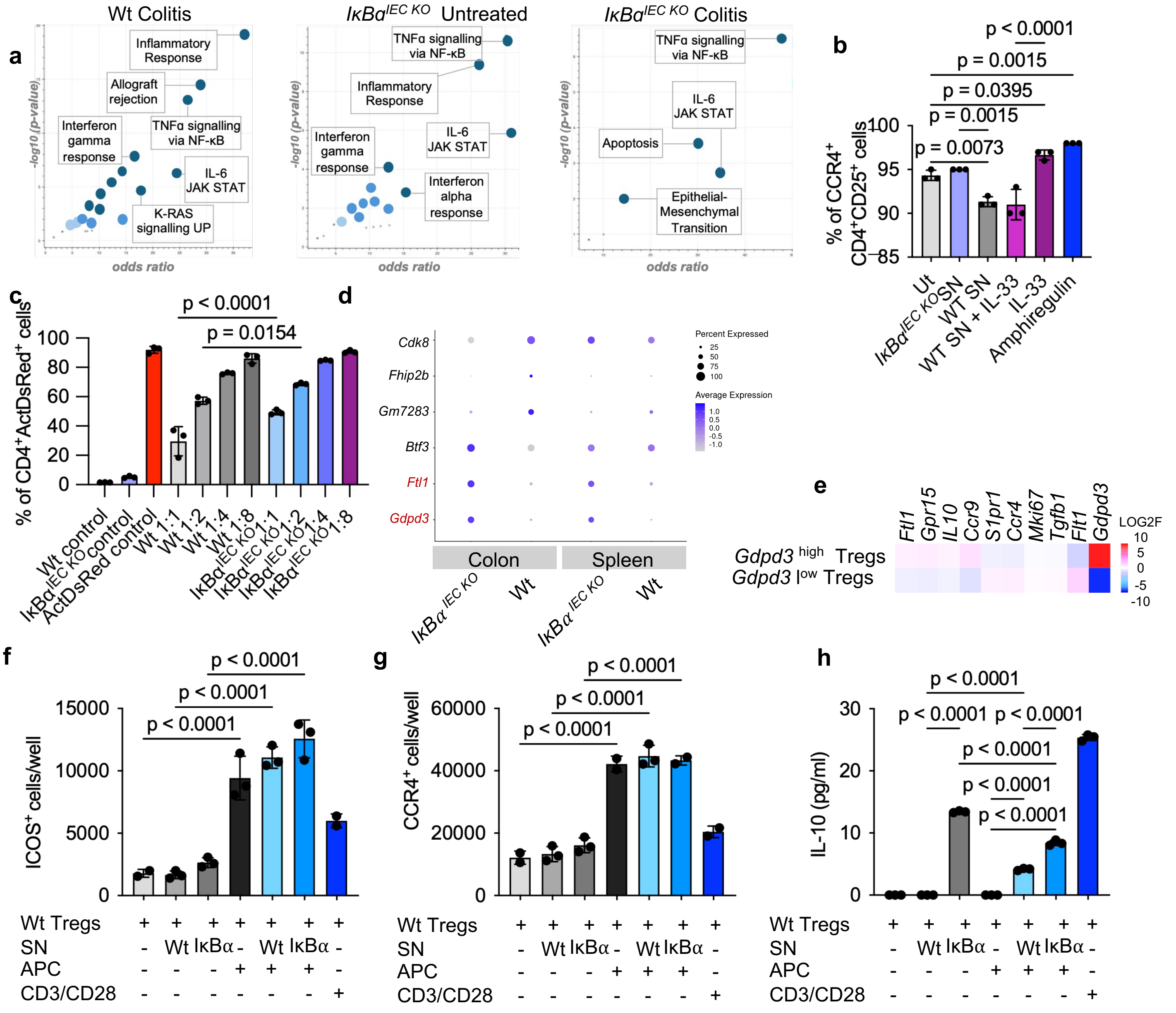

Supplementary Figure 7: a. Volcano plots from the MSigDB_Hallmark_2020 gene set based on Fig. 6a: point represents a single term, colour and size of bubble represents significance. Top terms indicated. **b.** CCR4 expression in expanded Treg cells. Isolated Treg cells treated with the media from Fig. 6e were analysed for CCR4 expression on day 3 via flow-cytometry. Relative CCR4 expression displayed in relation to total number of live CD4 cells. n = 3. Analysis: one-way ANOVA with Tukey's correction. **c.** Relative population of ActDsRed+ CD4+ T-cells (n = 2) after 4-day co-culture with Wt or *IκBα$^{IEC\ KO}$* Treg cells (n = 4 per group) in Treg cell baseline medium + 6 ng/ml IL-2 in the depicted cell to cell ratios. Control samples show non-mixed cell populations of Treg cells and ActDsRed CD4+ cells respectively. Analysis as in b. **d.** Genes with significant changes in expression between in colonic or splenic Treg cells in Wt versus *IκBα$^{IEC\ KO}$* mice based on Figure 6j. Marker of *IκBα$^{IEC\ KO}$* Tregs in red. **e.** Differential analysis from scRNA data: spleen Treg cells enriched for *Gdpd3* vs *Gdpd3 low* (no Gdpd3 detected). **f.** Wt Treg cells isolated as in Fig.6 and cultured with SN (supernatant from either Wt or *IκBα$^{IEC\ KO}$* organoids) and/or isolated primary CD11c+ cells (APC) or with CD3/CD28. Analysis as in b. Flow cytometry analysis of ICOS (left) and **g.** CCR4. Total number of cells shown. n = 6 mice, pool. Technical replicate n = 3. Organoids pulled from n = 3 mice. Analysis as in b. **h.** as in f. Treg cell SN collected and analysed by Il10 ELISA. Analysis as in b.
