# Supplementary material for: Epithelial NF-κB shapes protective Treg cell responses to restrain intestinal inflammation
Source: Nat Commun. 2026 Oct 20;17:10956. doi: 10.1038/s41467-026-78796-z (PMC13683377; doi:10.1038/s41467-026-78796-z)
Supplement: Supplementary file 4 [file 41467_2026_78796_MOESM2_ESM.pdf]

Description of Additional Supplementary Files

Title: Supplementary Data 1. scRNAseq

Description: Differential expression analysis of genes

Title: Supplementary Data 2. scRNAseq GO terms

Description: NF-κB target genes showing significant increase in expression in stroma, epithelium and immune cells

Title: Supplementary Data 3. NFKB target genes in bulk RNAseq

Description: analysis of NFKB target genes in intestinal epithelial crypts

Title: Supplementary Data 4. Secretome analysis of intestinal epithelial cells by proteome array

Description: ELISA-based analysis of secreted proteins

Title: Supplementary Data 5. Secreted targets coded by NF-κB genes

Description: analysis of secreted proteins in murine intestinal organoids by antibody array

Title: Supplementary Data 6. Secreted targets coded by NF-κB genes

Description: analysis of secreted proteins in human UC organoids by antibody array

Title: Supplementary Data 7. Patient data used for analysis

Description: age and sex of biopsy donors

Title: Supplementary Data 8. Antibody lot and catalogue number and primer sequence

Description: additional information about reagents
